# 6,*N*^2^-Diaryl-1,3,5-triazine-2,4-diamines: synthesis, antiproliferative activity and 3D-QSAR modeling†

**DOI:** 10.1039/d0ra00643b

**Published:** 2020-03-25

**Authors:** Ahmad Junaid, Felicia Phei Lin Lim, Lay Hong Chuah, Anton V. Dolzhenko

**Affiliations:** School of Pharmacy, Monash University Malaysia Jalan Lagoon Selatan Bandar Sunway Selangor Darul Ehsan 47500 Malaysia anton.dolzhenko@monash.edu; School of Pharmacy and Biomedical Sciences, Curtin Health Innovation Research Institute, Faculty of Health Sciences, Curtin University GPO Box U1987 Perth Western Australia 6845 Australia

## Abstract

A library of 126 compounds with a 6,*N*^2^-diaryl-1,3,5-triazine-2,4-diamine scaffold was prepared using a one-pot, microwave-assisted method from readily available cyanoguanidine, aromatic aldehydes and arylamines. The three-component condensation of these reagents in the presence of hydrochloric acid was followed by the treatment with a base, which promoted a rearrangement of the dihydrotriazine ring and its dehydrogenative aromatization. The antiproliferative properties of the prepared compounds were evaluated using three breast cancer cell lines. The most promising results were obtained in the growth inhibition of the triple negative MDA-MB231 breast cancer cells. The active compounds were also selective against cancer cells and did not affect growth of the non-cancerous MCF-10A breast cell line. Analyzing the structure–activity relationship within the series, we built a 3D-QSAR model for the further design of more potent anticancer compounds.

## Abstract 2

New compounds selectively targeting the triple negative MDA-MB231 breast cancer cells were used to build a 3D-QSAR model.

## Graphical abstract

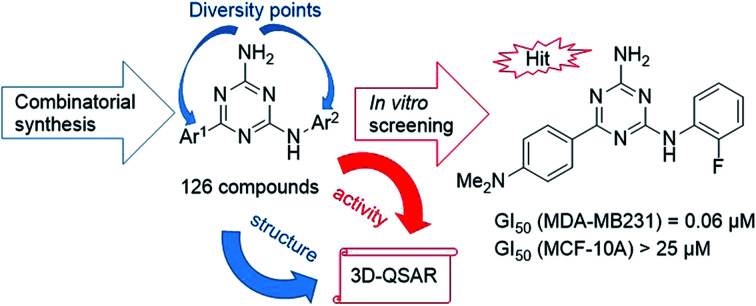

## Introduction

**Fig. 1:**
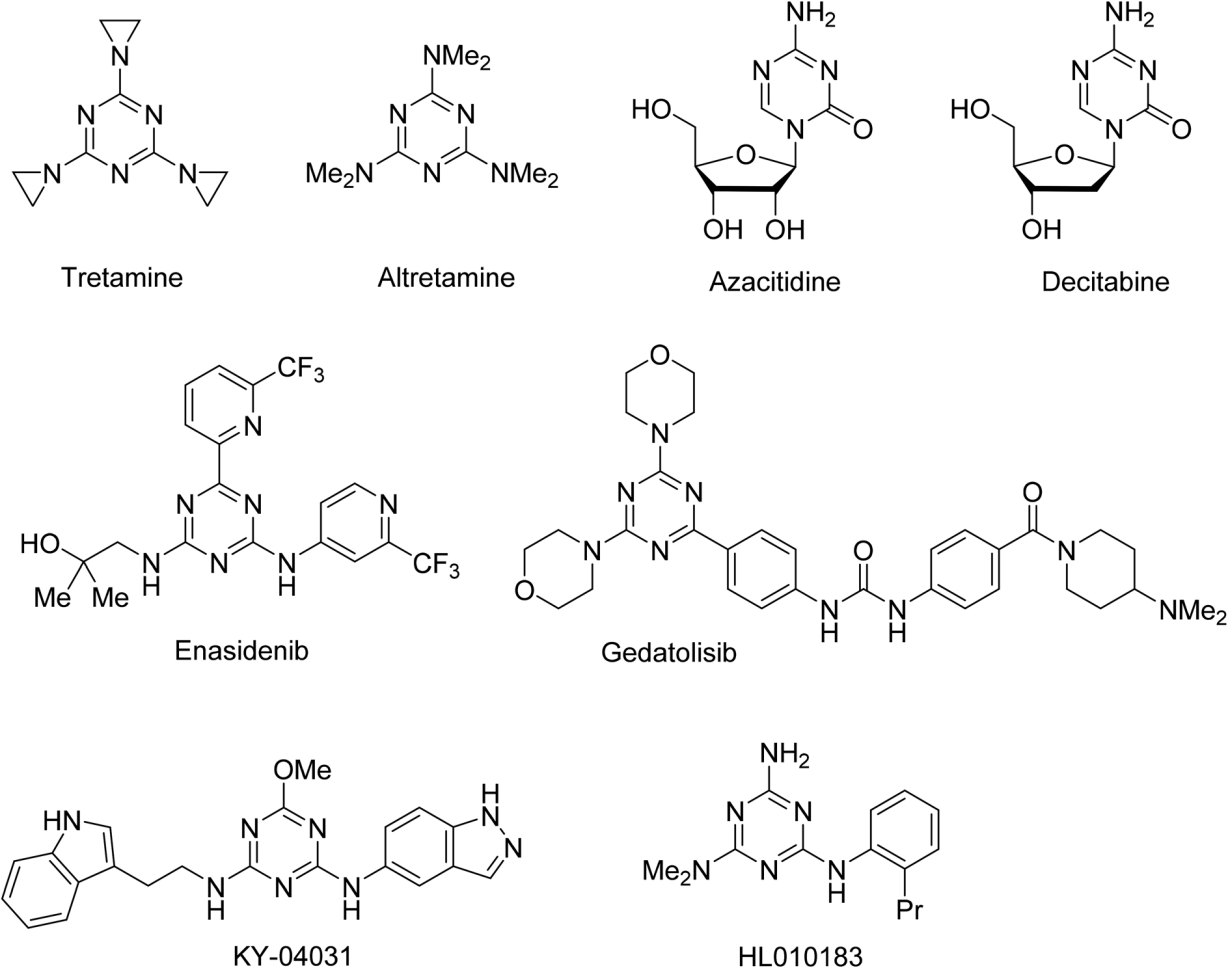
Selected anticancer 1,3,5-triazines.

The 1,3,5-triazine ring has been extensively explored as a scaffold for the design and construction of molecules with therapeutically useful properties.^1^ Particular advancements were made in the area of the anticancer agent development.^2^ Several 1,3,5-triazine based agents have been used for the treatment of various types of cancer, from earlier developed alkylating agents tretamine^3,4^ and altretamine^5^ to nucleic acid targeting nucleosides azacitidine^6^ and decitabine,^7,8^ and a recently approved inhibitor of isocitrate dehydrogenase 2, enasidenib^9,10^ (Fig. 1). An intensive exploration of the 1,3,5-triazine scaffold has continued and a number of notable anticancer 1,3,5-triazines have been recently developed, including those undergoing clinical trials gedatolisib inhibiting PI3K and mTOR kinases,^11,12^ PAK4 inhibitor KY-04031 effective against prostate cancer,^13^ and HL010183 particularly effective in the inhibition of proliferation and invasion of triple-negative breast cancer cells.^14,15^

Recently we reported a new effective synthesis of a library of 6,*N*^2^-diaryl-1,3,5-triazine-2,4-diamines, some of which demonstrated promising anticancer properties in preliminary assessment on the DU145 prostate cancer cell line.^16^ Inspired by these results, we further expanded the initial library to 126 compounds and performed antiproliferative screening of these compounds on three types of breast cancer cell lines. Herein, we report result of this work and attempt to build a QSAR model for the further design of more active compounds.

## Results and discussion

### Synthesis

**Scheme 1:**
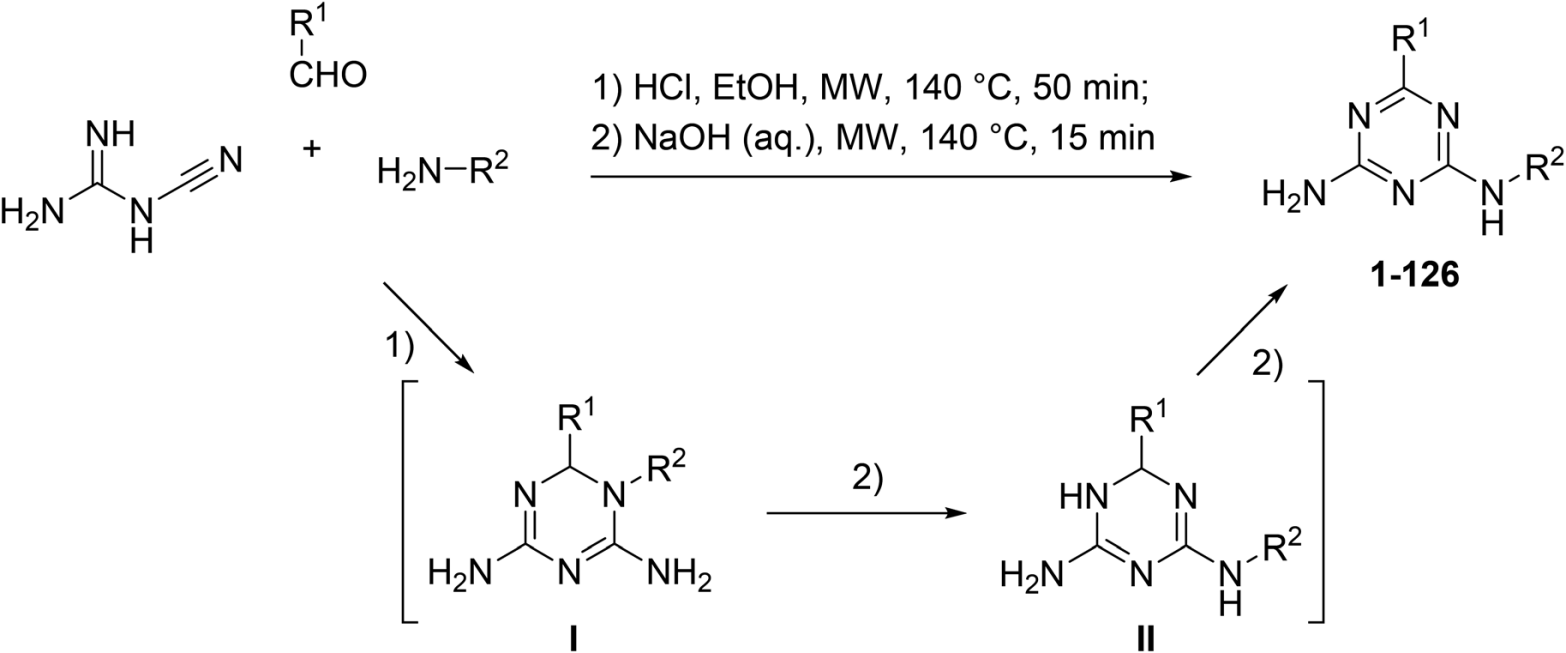
Synthesis of 6,*N*^2^-diaryl-1,3,5-triazine-2,4-diamines (1–126).

The microwave-assisted synthesis has been recognized as a highly valuable approach for the synthesis of 1,3,5-triazines.^17^ We applied focused microwave irradiation for the synthesis of 6,*N*^2^-diaryl-1,3,5-triazine-2,4-diamines using a recently developed one-pot method.^16^ Initially, a three-component reaction of cyanoguanidine, aromatic aldehydes and arylamines was carried out in the presence of hydrochloric acid under microwave irradiation. Without isolation, intermediates I were treated with a base to give products of the Dimroth rearrangement II, which, at the reaction conditions, underwent a spontaneous dehydrogenation and aromatization affording desired 6,*N*^2^-diaryl-1,3,5-triazine-2,4-diamines (1–126) (Scheme 1).

The developed protocol for the synthesis of 6,*N*^2^-diaryl-1,3,5-triazine-2,4-diamines (1–126) was rather general and convenient for the generation of libraries covering a sufficiently broad chemical space for the biological screening.

### Cytotoxic evaluation

The prepared compounds 1–126 were tested on three breast cancer cell lines namely, MDA-MB231, SKBR-3 and MCF-7 using MTT assay. SKBR-3 and MCF-7 cell lines are estrogen and progesterone hormone positive cell lines often used as a model for the hormone therapy.^18^ MDA-MB231 is triple negative breast cancer cell line, which is negative to estrogen and progesterone receptors and human epidermal growth factor receptor 2, a perfect model for chemotherapy.^18^ Initially, all the prepared compounds 1–126 were tested on these three cancer cell lines at the screening concentration (10 μM) and percentage of cell viability was calculated 72 h after the treatment (Table 1).

**Table 1:** Antiproliferative screening of 6,*N*^2^-diaryl-1,3,5-triazine-2,4-diamines (1–126) at 10 μM

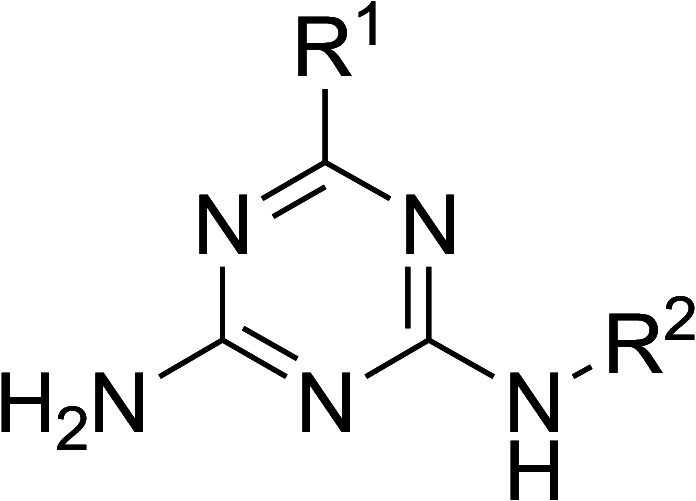					
Compd	R^1^	R^2^	Percentage of cell viability^a^		
			MDA-MB231	SKBR-3	MCF-7
1	Ph	Ph	87	81	90
2	Ph	2-FC_6_H_4_	82	83	99
3	Ph	4-FC_6_H_4_	81	84	100
4	Ph	2-ClC_6_H_4_	72	88	81
5	Ph	4-ClC_6_H_4_	65	94	100
6	Ph	4-BrC_6_H_4_	65	97	100
7	Ph	4-MeC_6_H_4_	98	87	87
8	Ph	2-MeOC_6_H_4_	78	97	91
9	Ph	4-MeOC_6_H_4_	98	96	84
10	Ph	4-CF_3_OC_6_H_4_	96	86	92
11	Ph	4-iPrC_6_H_4_	99	86	99
12	Ph	3-Pyridyl	59	78	87
13	3-FC_6_H_4_	Ph	100	100	89
14	3-FC_6_H_4_	4-FC_6_H_4_	50	100	78
15	3-FC_6_H_4_	4-ClC_6_H_4_	100	100	89
16	3-FC_6_H_4_	4-BrC_6_H_4_	43	89	80
17	3-FC_6_H_4_	4-MeC_6_H_4_	29	90	71
18	3-FC_6_H_4_	4-MeOC_6_H_4_	46	74	76
19	3-FC_6_H_4_	4-CF_3_OC_6_H_4_	50	100	76
20	3-FC_6_H_4_	4-iPrC_6_H_4_	100	100	92
21	4-FC_6_H_4_	Ph	83	94	100
22	4-FC_6_H_4_	2-FC_6_H_4_	83	85	100
23	4-FC_6_H_4_	4-FC_6_H_4_	81	100	93
24	4-FC_6_H_4_	2-ClC_6_H_4_	75	75	91
25	4-FC_6_H_4_	3-ClC_6_H_4_	73	88	95
26	4-FC_6_H_4_	4-ClC_6_H_4_	67	98	87
27	4-FC_6_H_4_	4-BrC_6_H_4_	96	100	94
28	4-FC_6_H_4_	4-MeC_6_H_4_	99	90	88
29	4-FC_6_H_4_	2-MeOC_6_H_4_	60	81	96
30	4-FC_6_H_4_	4-MeOC_6_H_4_	74	90	92
31	4-FC_6_H_4_	4-CF_3_OC_6_H_4_	88	92	87
32	4-FC_6_H_4_	4-iPrC_6_H_4_	94	100	84
33	4-FC_6_H_4_	3-Pyridyl	74	87	85
34	4-ClC_6_H_4_	Ph	81	100	100
35	4-ClC_6_H_4_	2-FC_6_H_4_	82	99	93
36	4-ClC_6_H_4_	4-FC_6_H_4_	49	98	100
37	4-ClC_6_H_4_	2-ClC_6_H_4_	99	89	99
38	4-ClC_6_H_4_	3-ClC_6_H_4_	99	92	84
39	4-ClC_6_H_4_	4-ClC_6_H_4_	97	100	100
40	4-ClC_6_H_4_	4-BrC_6_H_4_	80	100	92
41	4-ClC_6_H_4_	2-MeOC_6_H_4_	83	89	100
42	4-ClC_6_H_4_	4-MeOC_6_H_4_	61	90	85
43	4-ClC_6_H_4_	4-CF_3_OC_6_H_4_	93	83	100
44	4-ClC_6_H_4_	4-iPrC_6_H_4_	100	97	96
45	4-ClC_6_H_4_	3-Pyridyl	99	74	89
46	4-BrC_6_H_4_	2-FC_6_H_4_	75	91	100
47	4-BrC_6_H_4_	4-FC_6_H_4_	58	98	100
48	4-BrC_6_H_4_	2-ClC_6_H_4_	90	93	100
49	4-BrC_6_H_4_	4-ClC_6_H_4_	96	100	100
50	4-BrC_6_H_4_	4-MeC_6_H_4_	55	93	88
51	4-BrC_6_H_4_	2-MeOC_6_H_4_	88	89	96
52	4-BrC_6_H_4_	4-MeOC_6_H_4_	56	91	100
53	3-MeC_6_H_4_	4-FC_6_H_4_	100	100	78
54	3-MeC_6_H_4_	4-ClC_6_H_4_	100	93	76
55	3-MeC_6_H_4_	4-BrC_6_H_4_	83	96	75
56	3-MeC_6_H_4_	4-MeC_6_H_4_	12	62	67
57	3-MeC_6_H_4_	4-MeOC_6_H_4_	91	90	75
58	3-MeC_6_H_4_	4-CF_3_OC_6_H_4_	50	100	73
59	3-MeC_6_H_4_	4-iPrC_6_H_4_	87	93	76
60	4-MeC_6_H_4_	Ph	84	100	100
61	4-MeC_6_H_4_	2-FC_6_H_4_	50	79	73
62	4-MeC_6_H_4_	4-FC_6_H_4_	48	78	100
63	4-MeC_6_H_4_	4-BrC_6_H_4_	87	100	93
64	4-MeC_6_H_4_	4-MeC_6_H_4_	59	92	86
65	4-MeC_6_H_4_	2-MeOC_6_H_4_	97	88	87
66	4-MeC_6_H_4_	4-MeOC_6_H_4_	83	89	100
67	4-MeC_6_H_4_	4-CF_3_OC_6_H_4_	92	84	93
68	4-MeC_6_H_4_	4-iPrC_6_H_4_	76	91	100
69	4-MeC_6_H_4_	3-Pyridyl	74	79	88
70	4-MeOC_6_H_4_	2-FC_6_H_4_	9	46	63
71	4-MeOC_6_H_4_	4-MeOC_6_H_4_	56	93	95
72	4-MeOC_6_H_4_	4-FC_6_H_4_	68	100	100
73	4-MeOC_6_H_4_	2-ClC_6_H_4_	10	47	84
74	4-MeOC_6_H_4_	3-ClC_6_H_4_	22	45	86
75	4-MeOC_6_H_4_	4-ClC_6_H_4_	99	100	100
76	4-MeOC_6_H_4_	4-BrC_6_H_4_	96	100	91
77	4-MeOC_6_H_4_	3-MeC_6_H_4_	22	40	79
78	4-MeOC_6_H_4_	2-MeOC_6_H_4_	50	66	57
79	4-MeOC_6_H_4_	4-CF_3_OC_6_H_4_	74	84	95
80	4-MeOC_6_H_4_	4-iPrC_6_H_4_	92	86	100
81	4-MeOC_6_H_4_	3-Pyridyl	46	77	91
82	4-CF_3_C_6_H_4_	Ph	55	97	96
83	4-CF_3_C_6_H_4_	2-FC_6_H_4_	55	71	93
84	4-CF_3_C_6_H_4_	4-FC_6_H_4_	95	98	100
85	4-CF_3_C_6_H_4_	4-ClC_6_H_4_	90	77	99
86	4-CF_3_C_6_H_4_	4-MeC_6_H_4_	54	95	83
87	4-CF_3_C_6_H_4_	2-MeOC_6_H_4_	63	85	100
88	4-CF_3_C_6_H_4_	4-MeOC_6_H_4_	81	86	100
89	4-CF_3_OC_6_H_4_	Ph	59	90	95
90	4-CF_3_OC_6_H_4_	2-FC_6_H_4_	60	80	90
91	4-CF_3_OC_6_H_4_	4-FC_6_H_4_	53	86	100
92	4-CF_3_OC_6_H_4_	2-ClC_6_H_4_	74	86	96
93	4-CF_3_OC_6_H_4_	4-BrC_6_H_4_	83	96	90
94	4-CF_3_OC_6_H_4_	4-MeC_6_H_4_	79	87	97
95	4-CF_3_OC_6_H_4_	2-MeOC_6_H_4_	46	81	88
96	4-CF_3_OC_6_H_4_	4-MeOC_6_H_4_	69	88	87
97	4-CF_3_OC_6_H_4_	4-CF_3_OC_6_H_4_	92	80	100
98	4-CF_3_OC_6_H_4_	4-iPrC_6_H_4_	100	90	95
99	4-CF_3_OC_6_H_4_	3-Pyridyl	33	81	85
100	4-Me_2_NC_6_H_4_	Ph	21	61	80
101	4-Me_2_NC_6_H_4_	2-FC_6_H_4_	28	51	88
102	4-Me_2_NC_6_H_4_	4-FC_6_H_4_	32	88	90
103	4-Me_2_NC_6_H_4_	2-MeOC_6_H_4_	28	48	88
104	4-Me_2_NC_6_H_4_	4-iPrC_6_H_4_	70	87	100
105	4-*t*BuC_6_H_4_	Ph	74	100	96
106	4-*t*BuC_6_H_4_	4-FC_6_H_4_	97	84	88
107	4-*t*BuC_6_H_4_	4-ClC_6_H_4_	100	92	98
108	4-*t*BuC_6_H_4_	4-BrC_6_H_4_	59	98	99
109	4-*t*BuC_6_H_4_	4-MeOC_6_H_4_	91	85	93
110	4-BnOC_6_H_4_	Ph	51	75	100
111	4-BnOC_6_H_4_	4-FC_6_H_4_	69	84	89
112	4-BnOC_6_H_4_	4-BrC_6_H_4_	82	93	97
113	4-BnOC_6_H_4_	4-MeC_6_H_4_	82	94	100
114	4-BnOC_6_H_4_	4-CF_3_OC_6_H_4_	71	87	100
115	2-Thienyl	Ph	100	87	91
116	2-Thienyl	2-FC_6_H_4_	98	78	90
117	2-Thienyl	4-FC_6_H_4_	100	86	92
118	2-Thienyl	2-ClC_6_H_4_	100	78	91
119	2-Thienyl	4-ClC_6_H_4_	100	89	98
120	2-Thienyl	4-BrC_6_H_4_	46	87	88
121	2-Thienyl	4-MeC_6_H_4_	49	95	97
122	2-Thienyl	2-MeOC_6_H_4_	78	97	91
123	2-Thienyl	4-MeOC_6_H_4_	93	88	80
124	2-Thienyl	4-CF_3_OC_6_H_4_	100	86	85
125	2-Thienyl	4-iPrC_6_H_4_	100	86	89
126	2-Thienyl	3-Pyridyl	100	91	86

a Mean of three independent experiments.

The prepared 6,*N*^2^-diaryl-1,3,5-triazine-2,4-diamines selectively inhibited the triple negative breast cancer cells. It was observed that hormone independent cell line (MDA-MB231) was generally more sensitive to the treatment with 6,*N*^2^-diaryl-1,3,5-triazine-2,4-diamines, whereas hormone dependent cancer cell lines (SKBR-3 and MCF-7) were more resistant to the treatment with these compounds.

Compounds reducing the growth of the MDA-MB231 cancer cells (at concentration 10 μM) to 50% or less were selected for the evaluation of their 50% growth inhibitory concentrations (GI_50_). The growth inhibitory effect of the compounds was determined on breast tumor cell line (MDA-MB231, SKBR-3 and MCF-7) at different concentrations with methotrexate and nilotinib as positive controls (Table 2). All the tested compounds were more effective towards the MDA-MB231 cancer cell line than SKBR-3 and MCF-7 cells.

**Table 2:** Inhibition of cell growth by compounds selected after the initial screening

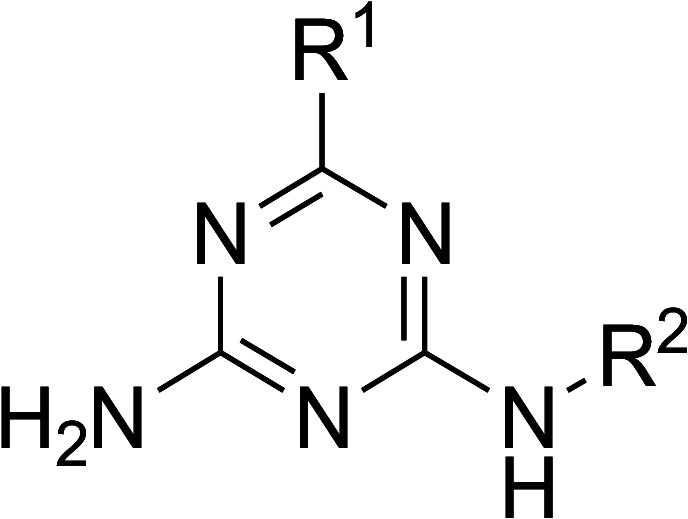						
Compound	R^1^	R^2^	GI_50_^a^ (μM) ± SEM^b^			
			MDA-MB231	SKBR-3	MCF-7	MCF-10A
14	3-FC_6_H_4_	4-FC_6_H_4_	9.21 ± 0.38	>20	>20	>25
16	3-FC_6_H_4_	4-BrC_6_H_4_	13.13 ± 0.91	17.16 ± 1.07	>20	>25
17	3-FC_6_H_4_	4-MeC_6_H_4_	3.96 ± 0.17	>20	>20	>25
18	3-FC_6_H_4_	4-MeOC_6_H_4_	6.18 ± 0.43	16.63 ± 1.38	>20	>25
19	3-FC_6_H_4_	4-CF_3_OC_6_H_4_	8.56 ± 0.59	>20	18.22 ± 1.28	>25
36	4-ClC_6_H_4_	4-FC_6_H_4_	14.14 ± 1.52	19.40 ± 2.39	>20	>25
56	3-MeC_6_H_4_	4-MeC_6_H_4_	0.17 ± 0.02	1.26 ± 0.18	>20	>25
58	3-MeC_6_H_4_	4-CF_3_OC_6_H_4_	15.24 ± 1.01	>20	>20	>25
61	4-MeC_6_H_4_	2-FC_6_H_4_	12.25 ± 1.15	>20	>20	>25
62	4-MeC_6_H_4_	4-FC_6_H_4_	10.68 ± 0.73	>20	20.15 ± 1.95	>25
70	4-MeOC_6_H_4_	2-FC_6_H_4_	0.32 ± 0.04	>20	>20	>25
73	4-MeOC_6_H_4_	2-ClC_6_H_4_	0.23 ± 0.03	1.10 ± 0.01	>20	>25
74	4-MeOC_6_H_4_	3-ClC_6_H_4_	1.33 ± 0.17	0.18 ± 0.04	18.30 ± 1.11	>25
77	4-MeOC_6_H_4_	3-MeC_6_H_4_	0.95 ± 0.04	3.38 ± 0.36	>20	>25
78	4-MeOC_6_H_4_	2-MeOC_6_H_4_	13.25 ± 0.93	2.15 ± 0.12	>20	>25
81	4-MeOC_6_H_4_	3-Pyridyl	14.01 ± 1.33	15.58 ± 1.06	>20	>25
95	4-CF_3_OC_6_H_4_	2-MeOC_6_H_4_	12.77 ± 0.76	>20	>20	>25
99	4-CF_3_OC_6_H_4_	3-Pyridyl	11.52 ± 1.51	>20	>20	>25
100	4-Me_2_NC_6_H_4_	Ph	0.36 ± 0.07	4.19 ± 0.37	>20	>25
101	4-Me_2_NC_6_H_4_	2-FC_6_H_4_	0.06 ± 0.001	0.29 ± 0.04	>20	>25
102	4-Me_2_NC_6_H_4_	4-FC_6_H_4_	7.20 ± 0.94	>20	>20	>25
103	4-Me_2_NC_6_H_4_	2-MeOC_6_H_4_	4.17 ± 0.33	3.63 ± 0.23	>20	>25
110	4-BnOC_6_H_4_	Ph	13.44 ± 1.18	>20	>20	>25
120	2-Thienyl	4-BrC_6_H_4_	11.73 ± 1.39	>20	>20	>25
121	2-Thienyl	4-MeC_6_H_4_	13.88 ± 1.74	>20	>20	>25
Methotrexate^c^			0.01 ± 0.001	ND	5.79 ± 0.47	ND
Nilotinib^c^			0.04 ± 0.001	9.60 ± 0.51	ND	ND

a Concentration (μM) required to inhibit tumor cell growth by 50%, values are mean of three independent experiments.

b Standard error of the mean.

c Positive controls, ND = not determined.

Analysis of the structure–activity relationship identified a pattern in types and combinations of R^1^ and R^2^ groups associated with the antiproliferative effect. In general, replacement of a phenyl at R^1^ with the 2-thienyl moiety or a phenyl at R^2^ with the 3-pyridyl ring demonstrated only slight or no improvement in the cell growth inhibition. Analyzing effect of the substituent at the phenyl rings, we found that antiproliferative properties typically required +M electron-donating groups in *para*-position of the R^1^ phenyl ring or a *meta*-substitution in the same ring. The tolerance of the activity to the R^2^ substituents depended on the type of the R^1^ substitution. The combination of a *para*-substituted phenyl as R^1^ with the *para*-substituted phenyl as R^2^ was detrimental for the activity (except R^2^ = 4-FC_6_H_4_). However, the antiproliferative effect of compounds with a *meta*-substituted phenyl as R^1^ was less sensitive to the position of substituents in the R^2^ moiety. These compounds retained good antiproliferative effect with the *para*-substituted phenyl as the R^2^ group. The most efficient for the antiproliferative activity against MDA-MB231 cells were combinations of *para*-methoxy or *para*-dimethylamino groups in the R^1^ phenyl ring and *ortho*-fluoro- or *ortho*-chlorophenyl group as R^2^ (compounds 70, 73, and 101). These compounds were also very active against SKBR-3 cells. Moreover, compound 73 was equipotent in the inhibition of MDA-MB231 and SKBR-3 cell growth. Compound 101 was identified as the most active among the tested compounds in its cytotoxic effect against MDA-MB231 cells with GI_50_ value of 0.06 μM.

All the compounds, selected for determination of GI_50_ values against MDA-MB231 cells, were also used for the experiments with MCF-10A normal breast cells. None of the tested compounds applied at concentration of 25 μM showed significant inhibition of the normal breast cell growth. These results indicated that the tested compounds were selectively active towards the breast cancer cells without any substantial effects on the normal breast cells.

### 3D-QSAR study

To comprehend the structural requirement controlling the antiproliferative activity, a 3D-QSAR model was built applying 3D-QSAR protocol of Discovery Studio v18 (ref. 19) to the experimentally obtained biological data. Twenty-five compounds, having GI_50_ values against MDA-MB231 breast cancer cell line in the range of 0.06 μM to 15.24 μM, were selected as model data set. The GI_50_ values of the compounds were converted into corresponding pGI_50_ values (−log[GI_50_]) using ‘Prepare dependent values’ protocol in Discovery Studio. The compounds were initially aligned to the minimum energy and then randomly divided into training set (∼80%) and test set (∼20%) by ‘Diverse molecule’ method in Discovery Studio.

**Fig. 2:**
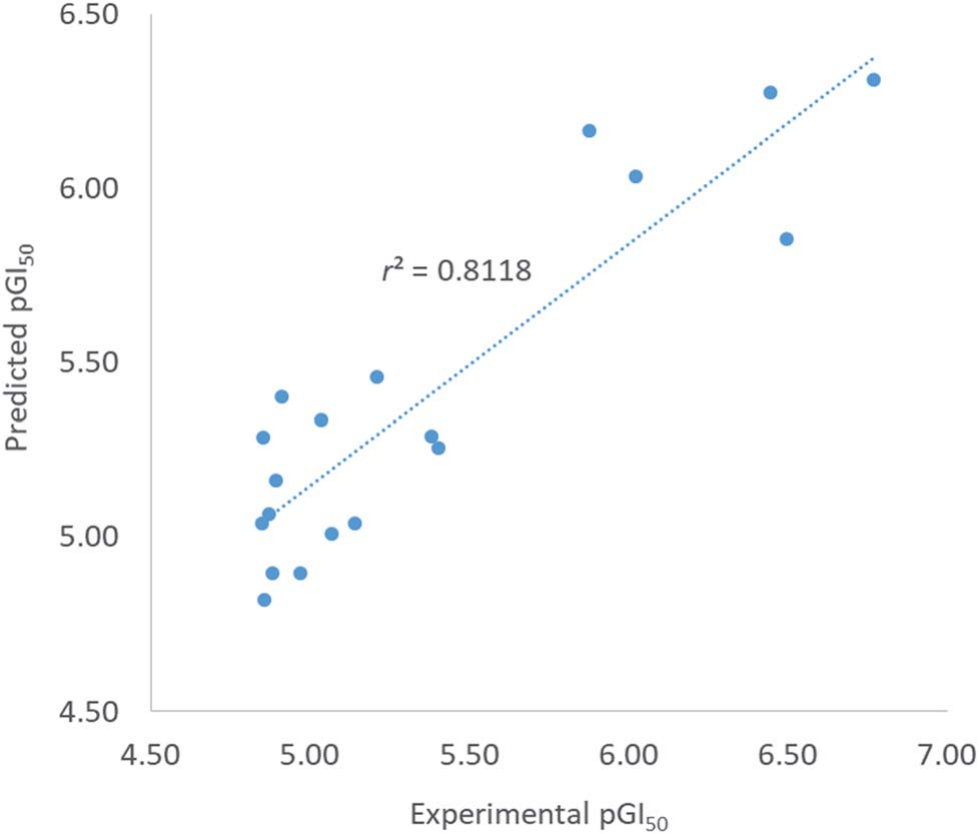
Plot of experimental *versus* predicted pGI_50_ activity of training set.

The QSAR model was built using ‘Create 3D-QSAR model’ protocol in Discovery Studio. The correlation coefficient *r*^2^ between the observed and predicted pGI_50_ values of the training set was found to be 0.81 proving acceptability of the built QSAR model. RMSE residual error was found to be 0.31 indicating a good ability of the built model to predict the GI_50_ values. The *r*^2^ value > 0.5 and RMSE residual error < 0.5 were considered to represent good model.^20,21^ Graphically, the model predictive potential is represented by the plot of the experimental pGI_50_*versus* predicted pGI_50_ values (Fig. 2). The pGI_50_ values predicted by this QSAR model and the residual errors for all 25 compounds are presented in Table 3.

**Table 3:** Experimental and predicted by 3D-QSAR model inhibitory activities of compounds^a^

Compound	Experimental pGI_50_	Predicted pGI_50_	Residual error
14	5.04	5.33	−0.30
16	4.88	4.90	−0.01
17	5.40	5.26	0.15
18	5.21	5.46	−0.25
19	5.07	5.01	0.06
36	4.85	5.04	−0.19
56	6.77	6.31	0.46
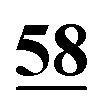	4.82	5.23	−0.41
61	4.91	5.40	−0.49
62	4.97	4.89	0.08
70	6.49	5.85	0.64
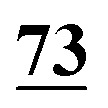	6.64	6.10	0.54
74	5.88	6.16	−0.29
77	6.02	6.03	−0.01
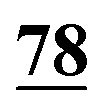	4.88	5.78	−0.90
71	4.85	5.29	−0.43
95	4.89	5.16	−0.27
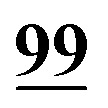	4.94	4.28	0.66
100	6.44	6.27	0.17
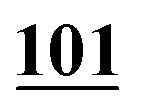	7.22	6.55	0.67
102	5.14	5.04	0.10
103	5.38	5.29	0.09
110	4.87	5.07	−0.20
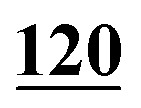	4.93	4.46	0.47
121	4.86	4.82	0.04

a Underlined compounds were randomly selected for test set.

**Fig. 3:**
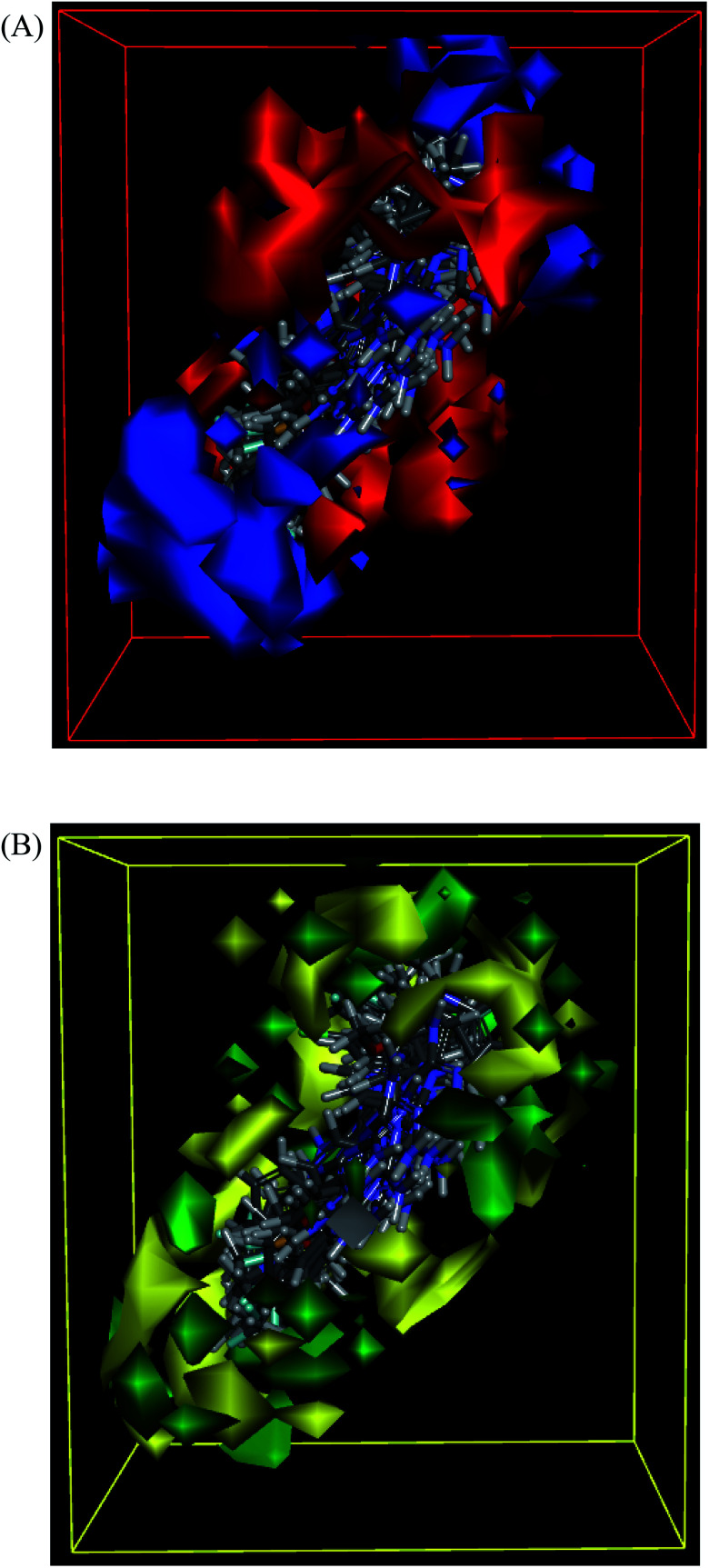
3D-QSAR model coefficients on electrostatic grid (A) and van der Waals grid (B).

The molecules aligned to the iso-surface of 3D-QSAR model on electrostatic potential grid and van der Waals grid are shown in Fig. 3. Red colour in the electrostatic grid (Fig. 3A) symbolizes that an increase in electron density in this region should increase the activity, while blue colour represents area where lower electron density is expected to be beneficial for the activity. Likewise, green contour in steric map (Fig. 3B) indicates a potential increase in the activity with sterically bulky groups in these regions, while yellow contour shows areas where an increase in the steric bulk would result in a lower activity.^21^

**Fig. 4:**
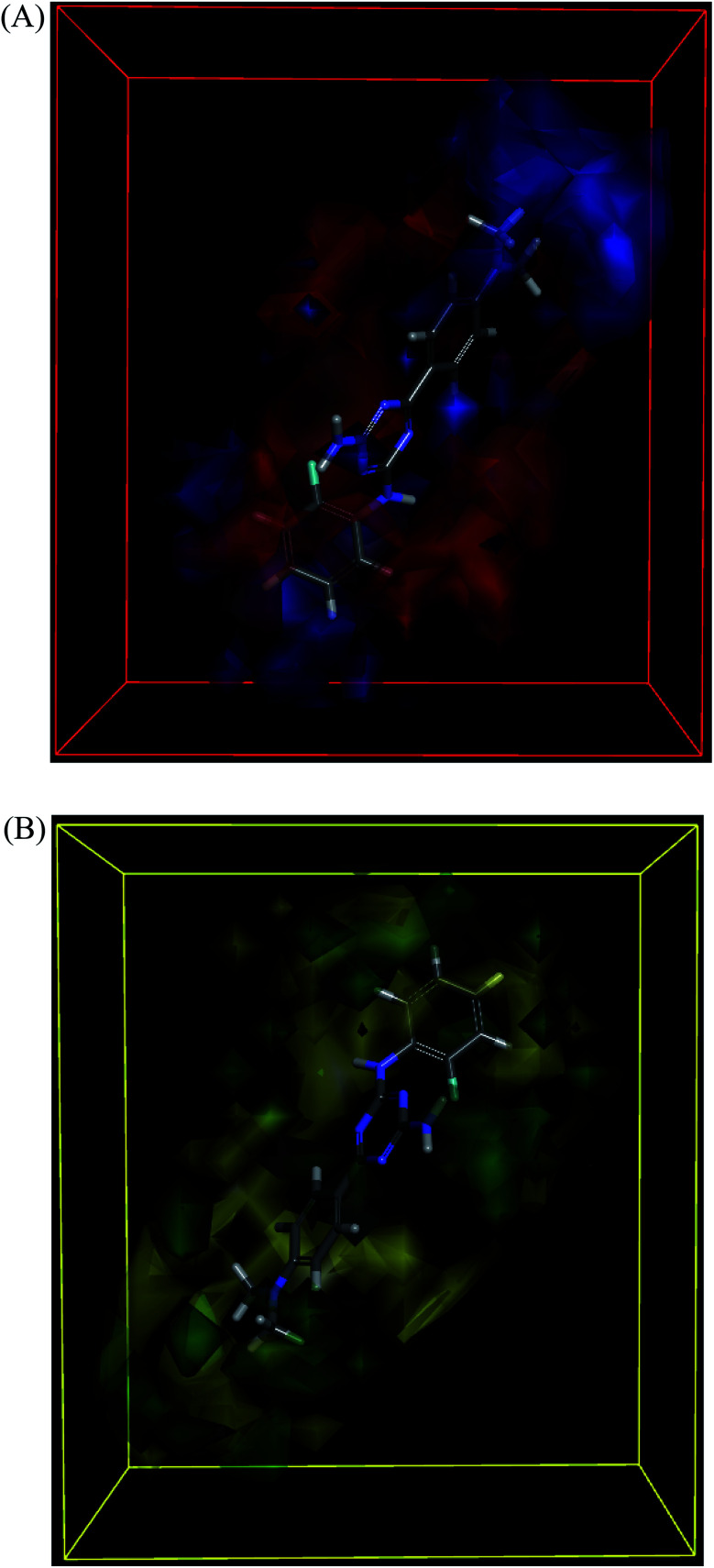
3D-QSAR model coefficients of compound 101 on electrostatic grid (A) and van der Waals grid (B).

The 3D-QSAR map suggested that compounds bearing a high electron density and bulky group at R^1^ position would show higher activity. Despite the high residual error (0.69) observed for compound 101, this 3D-QSAR map validated its highest activity as the dimethylamino group at the R^1^ phenyl ring clearly met the above description, particularly on the electrostatic grid. The good antiproliferative activity of compounds bearing a *para*-methoxy group at the R^1^ phenyl was also well aligned with the model. The positioning of the most active in the series compound 101 (GI_50_ = 0.06 μM) in the electrostatic and van der Waals grids is shown in Fig. 4.

## Conclusions

We synthesized a library of 6,*N*^2^-diaryl-1,3,5-triazine-2,4-diamines and evaluated their antiproliferative properties against three breast cancer cell lines. It was found that MDA-MB231 triple negative breast cancer cells were more sensitive to the prepared compounds than SKBR-3 and MCF-7 cells. The active compounds also demonstrated no inhibition on the growth of non-cancerous MCF-10A breast cells. The 3D-QSAR model constructed using the obtained data could be used for the further design of compoundstargeting triple negative breast cancer cells.

## Experimental

### General

Melting points (uncorrected) were determined on a Stuart™ SMP40 automatic melting point apparatus. ^1^H and ^13^C NMR spectra were recorded on a Bruker Fourier NMR spectrometer (300 MHz) using DMSO-*d*_6_ as a solvent and TMS as an internal reference. Microwave-assisted reactions were carried out in the closed vessel focused single mode using a Discover SP microwave synthesizer (CEM, USA) monitoring reaction temperature by the equipped IR sensor. The synthesis of compounds 1, 5–29, 31–33, 35–52, 54–56, 58, 60–64, 66–70, 72–83, 85–90, 92–110, 112–115, 117–121, 124–126 and their characterization were described earlier.^16^

### General method for the synthesis of 6,*N*^2^-diaryl-1,3,5-triazine-2,4-diamines (1–126)

To a solution of cyanoguanidine (0.21 g, 2.5 mmol), an aromatic aldehyde (2.5 mmol) and an arylamine (2.5 mmol) in EtOH (2 mL) in a 10 mL seamless pressure vial, conc. HCl (0.21 mL, 2.5 mmol) was added. The reaction mixture was heated at 140 °C for 50 min by irradiation in the Discover SP (CEM) microwave reactor operating at maximal microwave power up to 150 W. Then, an aq. solution of NaOH (5 N, 1 mL) was added to the reaction mixture and heating was continued for another 15 min at 140 °C. After cooling, the precipitated product was filtered, washed with water and recrystallized from a suitable solvent.

### *N* ^2^-(2-Fluorophenyl)-6-phenyl-1,3,5-triazine-2,4-diamine (2)

Yield 0.12 g, 30%. Mp 156–158 °C (EtOH). ^1^H NMR (300 MHz, DMSO-*d*_6_): *δ* 7.08 (2H, brs, NH_2_), 7.17–7.30 (3H, m, H-3′′, H-4′′ and H-5′′), 7.46–7.54 (3H, m, H-3′, H-4′ and H-5′), 7.75–7.81 (1H, m, H-6′′), 8.28 (2H, dd, *J* = 1.6 Hz, *J* = 8.1 Hz, H-2′ and H-6′), 9.02 (1H, s, NH); ^13^C NMR (75 MHz, DMSO-*d*_6_): *δ* 115.5 (d, ^2^*J*_CF_ = 19.4 Hz, C-3′′), 124.0 (d, ^3^*J*_CF_ = 3.7 Hz, C-6′′), 125.5 (d, ^3^*J*_CF_ = 7.5 Hz, C-4′′), 126.6 (d, ^4^*J*_CF_ = 1.5 Hz, C-5′′), 126.7 (d, ^2^*J*_CF_ = 12.0 Hz, C-1′′), 127.7 (C-3′ and C-5′), 128.2 (C-2′ and C-6′), 131.3 (C-4′), 136.6 (C-1′), 155.4 (d, ^1^*J*_CF_ = 245.9 Hz, C-2′′), 165.2 (C-4), 167.3 (C-6), 170.2 (C-2). Anal. calcd for C_15_H_12_FN_5_: C, 64.05; H, 4.30; N, 24.90. Found: C, 63.96; H, 4.41; N, 24.77.

### *N* ^2^-(4-Fluorophenyl)-6-phenyl-1,3,5-triazine-2,4-diamine (3)

Yield 0.25 g, 37%. Mp 172–174 °C (EtOH). ^1^H NMR (300 MHz, DMSO-*d*_6_): *δ* 7.13 (2H, brs, NH_2_), 7.15 (2H, dd, ^3^*J*_HF_ = 8.8 Hz, ^3^*J*_HH_ = 9.2 Hz, H-3′′ and H-5′′), 7.48–7.56 (3H, m, H-3′, H-4′ and H-5′), 7.85 (2H, dd, ^4^*J*_HF_ = 5.0 Hz, ^3^*J*_HH_ = 9.2 Hz, H-2′′ and H-6′′), 8.32 (2H, dd, *J* = 1.7 Hz, *J* = 8.1 Hz, H-2′ and H-6′), 9.57 (1H, s, NH); ^13^C NMR (75 MHz, DMSO-*d*_6_): *δ* 114.9 (d, ^2^*J*_CF_ = 22.0 Hz, C-3′′ and C-5′′), 121.6 (d, ^3^*J*_CF_ = 8.2 Hz, C-2′′ and C-6′′), 127.7 (C-3′ and C-5′), 128.2 (C-2′ and C-6′), 131.3 (C-4′), 136.2 (d, ^4^*J*_CF_ = 2.2 Hz, C-1′′), 136.7 (C-1′), 157.4 (d, ^1^*J*_CF_ = 238.6 Hz, C-4′′), 164.5 (C-4), 167.1 (C-6), 170.2 (C-2). Anal. calcd for C_15_H_12_FN_5_: C, 64.05; H, 4.30; N, 24.90. Found: C, 63.97; H, 4.38; N, 24.81.

### *N* ^2^-(2-Chlorophenyl)-6-phenyl-1,3,5-triazine-2,4-diamine (4)

Yield 0.26 g, 35%. Mp 82–84 °C (EtOH/H_2_O). ^1^H NMR (300 MHz, DMSO-*d*_6_): *δ* 7.10 (2H, brs, NH_2_), 7.20 (1H, ddd, *J* = 1.6 Hz, *J* = 7.5 Hz, *J* = 7.9 Hz, H-4′′), 7.37 (1H, ddd, *J* = 1.5 Hz, *J* = 7.5 Hz, *J* = 8.0 Hz, H-5′′), 7.46–7.54 (4H, m, H-3′, H-5′, H-4′ and H-3′′), 7.84 (1H, dd, *J* = 1.6 Hz, *J* = 8.0 Hz, H-6′′), 8.26 (2H, dd, *J* = 1.6 Hz, *J* = 6.7 Hz, H-2′ and H-6′), 8.75 (1H, s, NH); ^13^C NMR (75 MHz, DMSO-*d*_6_): *δ* 125.9 (C-6′′), 127.1 (C-4′′), 127.3 (C-2′′), 127.7 (C-3′ and C-5′), 128.0 (C-5′′), 128.2 (C-2′ and C-6′), 129.3 (C-3′′), 131.3 (C-4′), 135.7 (C-1′′), 136.5 (C-1′), 165.1 (C-4), 167.2 (C-6), 170.2 (C-2). Anal. calcd for C_15_H_12_ClN_5_: C, 60.51; H, 4.06; N, 23.52. Found: C, 60.39; H, 4.15; N, 23.44.

### 6-(4-Fluorophenyl)-*N*^2^-(4-methoxyphenyl)-1,3,5-triazine-2,4-diamine (30)

Yield 0.20 g, 30%. Mp 158–160 °C (MeCN). ^1^H NMR (300 MHz, DMSO-*d*_6_): *δ* 3.75 (3H, s, OCH_3_), 6.91 (2H, d, *J* = 9.0 Hz, H-3′′ and H-5′′), 7.06 (2H, brs, NH_2_), 7.33 (2H, dd, ^3^*J*_HF_ = 9.0 Hz, ^3^*J*_HH_ = 8.8 Hz, H-3′ and H-5′), 7.70 (2H, d, *J* = 8.9 Hz, H-2′′ and H-6′′), 8.36 (2H, dd, ^4^*J*_HF_ = 5.8 Hz, ^3^*J*_HH_ = 8.8 Hz, H-2′ and H-6′), 9.37 (1H, s, NH); ^13^C NMR (75 MHz, DMSO-*d*_6_): *δ* 55.1 (OCH_3_), 113.6 (C-3′′ and C-5′′), 115.1 (d, ^2^*J*_CF_ = 21.7 Hz, C-3′ and C-5′), 121.8 (C-2′′ and C-6′′), 130.1 (d, ^3^*J*_CF_ = 8.9 Hz, C-2′ and C-6′), 132.8 (C-1′′), 133.3 (d, ^4^*J*_CF_ = 2.7 Hz, C-1′), 154.7 (C-4′′), 164.1 (d, ^1^*J*_CF_ = 248.3 Hz, C-4′), 164.4 (C-4), 167.1 (C-6), 169.1 (C-2). Anal. calcd for C_16_H_14_FN_5_O: C, 61.73; H, 4.53; N, 22.50. Found: C, 61.60; H, 4.65; N, 22.34.

### 6-(4-Chlorophenyl)-*N*^2^-phenyl-1,3,5-triazine-2,4-diamine (34)

Yield 0.25 g, 33%. Mp 154–156 °C (EtOH). ^1^H NMR (300 MHz, DMSO-*d*_6_): *δ* 7.01 (1H, t, *J* = 7.9 Hz, H-4′′), 7.19 (2H, brs, NH_2_), 7.32 (2H, t, *J* = 7.9 Hz, H-3′′ and H-5′′), 7.60 (2H, d, *J* = 8.6 Hz, H-3′ and H-5′), 7.85 (2H, d, *J* = 7.9 Hz, H-2′′ and H-6′′), 8.33 (2H, d, *J* = 8.7 Hz, H-2′ and H-6′), 9.58 (1H, s, NH); ^13^C NMR (75 MHz, DMSO-*d*_6_): *δ* 120.0 (C-2′′ and C-6′′), 122.0 (C-1′′), 128.4 (C-3′, C-5′, C-3′′ and C-5′′), 129.5 (C-2′ and C-6′), 135.6 (C-1′), 136.1 (C-4′), 139.8 (C-4′′), 164.5 (C-4), 167.1 (C-6), 169.2 (C-2). Anal. calcd for C_15_H_12_ClN_5_: C, 60.51; H, 4.06; N, 23.52. Found: C, 60.46; H, 4.20; N, 23.36.

### *N* ^2^-(4-Fluorophenyl)-6-(3-methylphenyl)-1,3,5-triazine-2,4-diamine (53)

Yield 0.25 g, 35%. Mp 138–139 °C (EtOH/H_2_O). ^1^H NMR (300 MHz, DMSO-*d*_6_): *δ* 2.40 (3H, s, CH_3_), 7.13 (2H, brs, NH_2_), 7.15 (2H, dd, ^3^*J*_HF_ = 8.9 Hz, ^3^*J*_HH_ = 8.9 Hz, H-3′′ and H-5′′), 7.37–7.42 (2H, m, H-4′ and H-5′), 7.85 (2H, dd, ^4^*J*_HF_ = 5.0 Hz, ^3^*J*_HH_ = 9.1 Hz, H-2′′ and H-6′′), 8.10–8.16 (2H, m, H-2′ and H-6′), 9.56 (1H, s, NH); ^13^C NMR (75 MHz, DMSO-*d*_6_): *δ* 21.0 (CH_3_), 114.8 (d, ^2^*J*_CF_ = 21.6 Hz, C-2′′ and C-6′′), 121.5 (d, ^3^*J*_CF_ = 7.5 Hz, C-3′′ and C-5′′), 124.9 (C-2′), 128.1 (C-6′), 128.3 (C-5′), 131.9 (C-4′), 136.3 (d, ^4^*J*_CF_ = 3.0 Hz, C-1′′), 136.7 (C-3′), 137.3 (C-1′), 157.4 (d, ^1^*J*_CF_ = 238.4 Hz, C-4′′), 164.4 (C-2), 167.1 (C-4), 170.2 (C-6). Anal. calcd for C_16_H_14_FN_5_: C, 65.07; H, 4.78; N, 23.71. Found: C, 64.95; H, 4.91; N, 23.58.

### *N* ^2^-(4-Methoxyphenyl)-6-(3-methylphenyl)-1,3,5-triazine-2,4-diamine (57)

Yield 0.22 g, 30%. Mp 150–152 °C (EtOH/H_2_O). ^1^H NMR (300 MHz, DMSO-*d*_6_): *δ* 2.39 (3H, s, CH_3_), 3.74 (OCH_3_), 6.89 (2H, d, *J* = 9.0 Hz, H-3′′ and H-5′′), 7.02 (2H, brs, NH_2_), 7.34–7.41 (2H, m, H-4′ and H-5′), 7.71 (2H, d, *J* = 9.1 Hz, H-2′′ and H-6′′), 8.09–8.15 (2H, m, H-2′ and H-6′), 9.34 (1H, s, NH); ^13^C NMR (75 MHz, DMSO-*d*_6_): *δ* 21.0 (CH_3_), 55.1 (OCH_3_), 113.6 (C-3′′ and C-5′′), 121.6 (C-2′′ and C-6′′), 124.9 (C-2′), 128.1 (C-6′), 128.3 (C-5′), 131.8 (C-1′′), 132.9 (C-4′), 136.8 (C-3′), 137.2 (C-1′), 154.5 (C-4′′), 164.4 (C-2), 167.1 (C-4), 170.1 (C-6). Anal. calcd for C_17_H_17_N_5_O: C, 66.43; H, 5.58; N, 22.79. Found: C, 66.34; H, 5.70; N, 22.64.

### *N* ^2^-(4-Isopropylphenyl)-6-(3-methylphenyl)-1,3,5-triazine-2,4-diamine (59)

Yield 0.36 g, 45%. Mp 134–136 °C (EtOH/H_2_O). ^1^H NMR (300 MHz, DMSO-*d*_6_): *δ* 1.20 (6H, d, *J* = 6.9 Hz, (CH_3_)_2_), 2.40 (3H, s, CH_3_), 2.85 (1H, m, *J* = 7.0 Hz, CH), 7.08 (2H, brs, NH_2_), 7.17 (2H, d, *J* = 8.5 Hz, H-3′′ and H-5′′), 7.34–7.42 (2H, m, H-4′ and H-5′), 7.75 (2H, d, *J* = 8.6 Hz, H-2′′ and H-6′′), 8.12–8.18 (2H, m, H-2′ and H-6′), 9.44 (1H, s, NH); ^13^C NMR (75 MHz, DMSO-*d*_6_): *δ* 21.0 (CH_3_), 24.0 ((CH_3_)_2_), 32.8 (CH), 120.1 (C-2′′ and C-6′′), 125.0 (C-2′), 126.0 (C-3′′ and C-5′′), 128.1 (C-6′), 128.3 (C-5′), 131.9 (C-4′), 136.8 (C-3′), 137.2 (C-1′), 137.6 (C-1′′), 142.0 (C-4′′), 164.5 (C-2), 167.1 (C-4), 170.2 (C-6). Anal. calcd for C_19_H_21_N_5_: C, 71.45; H, 6.63; N, 21.93. Found: C, 71.38; H, 6.69; N, 21.85.

### *N* ^2^-(4-Methoxyphenyl)-6-(4-methylphenyl)-1,3,5-triazine-2,4-diamine (65)

Yield 0.45 g, 60%. Mp 168–170 °C (EtOH). ^1^H NMR (300 MHz, DMSO-*d*_6_): *δ* 2.38 (3H, s, CH_3_), 3.74 (3H, s, OCH_3_), 6.90 (2H, d, *J* = 9.0 Hz, H-3′′ and H-5′′), 7.00 (2H, brs, NH_2_), 7.30 (2H, d, *J* = 8.2 Hz, H-3′ and H-5′), 7.71 (2H, d, *J* = 9.0 Hz, H-2′′ and H-6′′), 8.21 (2H, d, *J* = 8.1 Hz, H-2′ and H-6′), 9.32 (1H, s, NH); ^13^C NMR (75 MHz, DMSO-*d*_6_): *δ* 21.0 (CH_3_), 55.1 (OCH_3_), 113.6 (C-3′′ and C-5′′), 121.6 (C-2′′ and C-6′′), 127.7 (C-3′ and C-5′), 128.8 (C-2′ and C-6′), 132.9 (C-1′′), 134.1 (C-1′), 141.1 (C-4′), 154.5 (C-4′′), 164.5 (C-4), 167.1 (C-6), 170.0 (C-2). Anal. calcd for C_17_H_17_N_5_O: C, 66.43; H, 5.58; N, 22.79. Found: C, 66.34; H, 5.66; N, 22.70.

### *N* ^2^,6-Bis(4-methoxyphenyl)-1,3,5-triazine-2,4-diamine (71)

Yield 0.30 g, 38%. Mp 163–165 °C (MeCN). ^1^H NMR (300 MHz, DMSO-*d*_6_): *δ* 3.74 (3H, s, OCH_3_), 3.84 (3H, s, OCH_3_), 6.90 (2H, d, *J* = 9.0 Hz, H-3′′ and H-5′′), 6.94 (2H, brs, NH_2_), 7.05 (2H, d, *J* = 8.9 Hz, H-3′ and H-5′), 7.71 (2H, d, *J* = 9.0 Hz, H-2′′ and H-6′′), 8.28 (2H, d, *J* = 8.9 Hz, H-2′ and H-6′), 9.28 (1H, s, NH); ^13^C NMR (75 MHz, DMSO-*d*_6_): *δ* 55.1 (OCH_3_), 55.2 (OCH_3_), 113.5 (C-3′′ and C-5′′), 113.6 (C-3′ and C-5′), 121.6 (C-2′′ and C-6′′), 129.1 (C-1′), 129.5 (C-2′ and C-6′), 133.0 (C-1′′), 154.5 (C-4′), 161.8 (C-4′′), 164.4 (C-4), 167.0 (C-6), 169.6 (C-2). Anal. calcd for C_17_H_17_N_5_O_2_: C, 63.15; H, 5.30; N, 21.66. Found: C, 63.02; H, 5.41; N, 21.57.

### *N* ^2^-(4-Fluorophenyl)-6-(4-(trifluoromethyl)phenyl)-1,3,5-triazine-2,4-diamine (84)

Yield 0.21 g, 28%. Mp 150–152 °C (EtOH). ^1^H NMR (300 MHz, DMSO-*d*_6_): *δ* 7.16 (2H, dd, ^3^*J*_HF_ = 8.9 Hz, ^3^*J*_HH_ = 8.9 Hz, H-3′′ and H-5′′), 7.29 (2H, brs, NH_2_), 7.85 (2H, dd, ^4^*J*_HF_ = 5.1 Hz, ^3^*J*_HH_ = 9.1 Hz, H-2′′ and H-6′′), 7.90 (2H, d, ^3^*J* = 8.6 Hz, H-3′ and H-5′), 8.50 (2H, d, *J* = 8.6 Hz, H-2′ and H-6′), 9.70 (1H, s, NH); ^13^C NMR (75 MHz, DMSO-*d*_6_): *δ* 114.9 (d, ^2^*J*_CF_ = 22.5 Hz, C-3′′ and C-5′′), 121.8 (d, ^3^*J*_CF_ = 7.5 Hz, C-2′′ and C-6′′), 124.1 (q, ^1^*J*_CF_ = 271.5 Hz, CF_3_), 125.3 (q, ^3^*J*_CF_ = 3.7 Hz, C-3′ and C-5′), 128.4 (C-1′), 131.1 (q, ^2^*J*_CF_ = 31.6 Hz, C-4′), 136.1 (d, ^4^*J*_CF_ = 2.2 Hz, C-1′′), 140.6 (q, ^4^*J*_CF_ = 1.5 Hz, C-2′ and C-6′), 157.6 (d, ^1^*J*_CF_ = 238.8 Hz, C-4′′), 164.5 (C-4), 167.1 (C-6), 169.0 (C-2). Anal. calcd for C_16_H_11_F_4_N_5_: C, 55.02; H, 3.17; N, 20.05. Found: C, 54.88; H, 3.26; N, 19.90.

### *N* ^2^-(4-Fluorophenyl)-6-(4-(trifluoromethoxy)phenyl)-1,3,5-triazine-2,4-diamine (91)

Yield 0.32 g, 58%. Mp 139–140 °C (EtOH). ^1^H NMR (300 MHz, DMSO-*d*_6_): *δ* 7.16 (2H, dd, ^3^*J*_HF_ = 8.9 Hz, ^3^*J*_HH_ = 8.9 Hz, H-3′′ and H-5′′), 7.22 (2H, brs, NH_2_), 7.51 (2H, d, *J* = 8.0 Hz, H-3′ and H-5′), 7.85 (2H, dd, ^4^*J*_HF_ = 5.0 Hz, ^3^*J*_HH_ = 9.0 Hz, H-2′′ and H-6′′), 8.42 (2H, d, *J* = 8.9 Hz, H-2′ and H-6′), 9.64 (1H, s, NH); ^13^C NMR (75 MHz, DMSO-*d*_6_): *δ* 114.9 (d, ^2^*J*_CF_ = 22.1 Hz, C-3′′ and C-5′′), 119.4 (q, ^1^*J*_CF_ = 154.2 Hz, OCF_3_), 120.5 (C-3′ and C-5′), 121.7 (d, ^3^*J*_CF_ = 7.4 Hz, C-2′′ and C-6′′), 129.8 (C-2′ and C-6′), 135.8 (C-1′), 136.1 (d, ^4^*J*_CF_ = 2.2 Hz, C-1′′), 150.6 (q, ^3^*J*_CF_ = 1.5 Hz, C-4′), 157.5 (d, ^1^*J*_CF_ = 239.0 Hz, C-4′′), 164.5 (C-4), 167.1 (C-6), 169.0 (C-2). Anal. calcd for C_16_H_11_F_4_N_5_O: C, 52.61; H, 3.04; N, 19.17. Found: C, 52.55; H, 3.21; N, 18.98.

### 6-(4-(Benzyloxy)phenyl)-*N*^2^-(4-fluorophenyl)-1,3,5-triazine-2,4-diamine (111)

Yield 0.53 g, 55%. Mp 177–179 °C (EtOH). ^1^H NMR (300 MHz, DMSO-*d*_6_): *δ* 5.19 (2H, s, CH_2_), 7.07 (2H, brs, NH_2_), 7.12–7.18 (4H, m, H-3′, H-5′, H-3′′ and H-5′′), 7.35–7.50 (5H, m, Ph), 7.86 (2H, dd, ^4^*J*_HF_ = 5.0 Hz, ^3^*J*_HH_ = 9.1 Hz, H-2′′ and H-6′′), 8.29 (2H, d, *J* = 8.9 Hz, H-2′ and H-6′), 9.52 (1H, s, NH); ^13^C NMR (75 MHz, DMSO-*d*_6_): *δ* 69.3 (CH_2_), 114.4 (C-3′ and C-5′), 114.8 (d, ^2^*J*_CF_ = 22.3 Hz, C-3′′ and C-5′′), 121.5 (d, ^3^*J*_CF_ = 7.4 Hz, C-2′′ and C-6′′), 127.7 (C-2′′′ and C-6′′′), 127.9 (C-4′′′), 128.4 (C-3′′′ and C-5′′′), 129.2 (C-1′), 129.5 (C-2′ and C-6′), 136.4 (d, ^4^*J*_CF_ = 2.2 Hz, C-1′′), 136.7 (C-1′′′), 157.3 (d, ^1^*J*_CF_ = 238.5 Hz, C-4′′) 161.0 (C-4′), 164.4 (C-4), 167.0 (C-6), 169.8 (C-2). Anal. calcd for C_22_H_18_FN_5_O: C, 68.21; H, 4.68; N, 18.08. Found: C, 68.08; H, 4.80; N, 17.96.

### *N* ^2^-(2-Fluorophenyl)-6-(thiophen-2-yl)-1,3,5-triazine-2,4-diamine (116)

Yield 0.32 g, 45%. Mp 165–167 °C (EtOH/H_2_O). ^1^H NMR (300 MHz, DMSO-*d*_6_): *δ* 7.09 (2H, brs, NH_2_), 7.15–7.27 (4H, m, H-4′, H-3′′, H-4′′ and H-5′′), 7.72–7.78 (2H, m, H-5′ and H-6′′), 7.87 (1H, dd, *J* = 1.1 Hz, *J* = 3.6 Hz, H-3′), 9.04 (1H, s, NH); ^13^C NMR (75 MHz, DMSO-*d*_6_): *δ* 115.5 (d, ^2^*J*_CF_ = 19.4 Hz, C-3′′), 124.0 (d, ^3^*J*_CF_ = 3.7 Hz, C-6′′), 125.5 (d, ^3^*J*_CF_ = 7.6 Hz, C-4′′), 126.5 (d, ^2^*J*_CF_ = 11.9 Hz, C-1′′), 126.6 (d, ^4^*J*_CF_ = 2.2 Hz, C-5′′), 128.0 (C-4′), 129.2 (C-5′), 130.9 (C-3′), 142.4 (C-1′), 155.4 (d, ^1^*J*_CF_ = 246.3 Hz, C-2′′), 164.8 (C-4), 166.6 (C-6), 166.9 (C-2). Anal. calcd for C_13_H_10_FN_5_S: C, 54.35; H, 3.51; N, 24.38. Found: C, 54.22; H, 3.75; N, 24.26.

### *N* ^2^-(2-Methoxyphenyl)-6-(thiophen-2-yl)-1,3,5-triazine-2,4-diamine (122)

Yield 0.43 g, 58%. Mp 165–167 °C (MeCN). ^1^H NMR (300 MHz, DMSO-*d*_6_): *δ* 3.87 (3H, s, OCH_3_), 6.94–7.00 (1H, m, H-3′′), 7.04–7.08 (2H, m, H-4′′ and H-5′′), 7.18 (2H, brs, NH_2_), 7.21 (1H, dd, *J* = 3.7 Hz, *J* = 5.0 Hz, H-4′), 7.77 (1H, dd, *J* = 1.2 Hz, *J* = 5.0 Hz, H-5′), 7.92 (1H, dd, *J* = 1.3 Hz, *J* = 3.7 Hz, H-3′), 7.97 (1H, s, NH), 8.21 (1H, d, *J* = 7.3 Hz, H-6′′); ^13^C NMR (75 MHz, DMSO-*d*_6_): *δ* 55.7 (OCH_3_), 110.9 (C-3′′), 120.3 (C-5′′), 121.8 (C-6′′), 123.5 (C-4′′), 127.7 (C-1′′), 128.1 (C-4′), 129.3 (C-5′), 131.0 (C-3′), 142.3 (C-1′), 149.6 (C-2′′), 164.2 (C-4), 166.6 (C-6), 166.9 (C-2). Anal. calcd for C_14_H_13_N_5_OS: C, 56.17; H, 4.38; N, 23.40. Found: C, 56.08; H, 4.50; N, 23.26.

### *N* ^2^-(4-Methoxyphenyl)-6-(thiophen-2-yl)-1,3,5-triazine-2,4-diamine (123)

Yield 0.52 g, 70%. Mp 139–141 °C (EtOH/H_2_O). ^1^H NMR (300 MHz, DMSO-*d*_6_): *δ* 3.74 (3H, s, OCH_3_), 6.88 (2H, d, *J* = 9.0 Hz, H-3′′ and H-5′′), 7.06 (2H, brs, NH_2_), 7.20 (1H, dd, *J* = 3.7 Hz, *J* = 5.0 Hz, H-4′), 7.70 (2H, d, *J* = 9.1 Hz, H-2′′ and H-6′′), 7.76 (1H, dd, *J* = 1.3 Hz, *J* = 5.0 Hz, H-5′), 7.89 (1H, dd, *J* = 1.3 Hz, *J* = 3.7 Hz, H-3′), 9.35 (1H, s, NH); ^13^C NMR (75 MHz, DMSO-*d*_6_): *δ* 55.1 (OCH_3_), 113.5 (C-3′′ and C-5′′), 121.6 (C-2′′ and C-6′′), 128.0 (C-4′), 129.0 (C-5′), 130.7 (C-3′), 132.8 (C-1′′), 142.6 (C-1′), 154.5 (C-4′′), 164.0 (C-4), 166.3 (C-6), 166.7 (C-2). Anal. calcd for C_14_H_13_N_5_OS: C, 56.17; H, 4.38; N, 23.40. Found: C, 56.05; H, 4.54; N, 23.23.

### Cytotoxicity evaluation

The cytotoxic activity of 6,*N*^2^-diaryl-1,3,5-triazine-2,4-diamines (1–126) was evaluated against three breast carcinoma cell lines (MDA-MB231, SKBR-3, and MCF-7) and normal breast cell line (MCF-10A) by MTT assay.^22^ All cells were obtained from the American Type Culture Collection and were grown in 10% fetal bovine serum and 1% pen-strep antibiotic supplemented media (DMEM for MDA-MB231 and SKBR-3, RPMI for MCF-7, and MEGM for MCF-10A). For MTT assay, 20 000 to 75 000 cells per mL (based on the doubling time for each cell line) were seeded in 96 well plates and incubated for 24 h at 37 °C in 5% CO_2_ incubator. Then, compounds at different concentrations were added followed by the incubation at 37 °C for 72 h. After that, MTT solution (0.5 mg mL^−1^) was added and the plates were incubated for another 4 h. The supernatant was then discarded and 100 μL of DMSO was added to each well. The plates were then read by Tecan NanoQuant (model: infinite m200 pro) plate reader and absorbance was measured at 570 nm. GI_50_ values were calculated using sigmoidal concentration–response curves (see ESI†) generated using the GraphPad Prism 7 program. Three independent experiments were carried out and the data were represented as mean of the three experiments.

### Building 3D-QSAR model

Out of 25 compounds, 19 compounds were utilized as a training set for building QSAR model. To assess reliability of the prepared model, an external validation was performed using remaining 6 compounds as a test set. The compounds were randomly divided into training and test set *via* ‘Generate training and test set’ module in Discover Studio v18. The selected test set included compounds 58, 73, 78, 99, 101, and 120.

For the model construction, the GI_50_ values of the compounds on MDA-MB231 were converted to the negative logarithmic scale (pGI_50_). The compounds were aligned to the minimum energy using the ‘Align small molecules’ protocol in the Discovery Studio. Steric (50%) and electrostatic (50%) fields were used to align the compounds.

In Discovery Studio, the CHARMm force field was used and the electrostatic potential and the van der Waals potential were treated as separate terms. A +1*e* point charge was used as the electrostatic potential probe and distance-dependent dielectric constant was used to mimic the solvation effect. For the van der Waals potential, a carbon atom with a 1.5 Å radius was used as a probe. The truncation for both the steric and electrostatic energies was set to 30 kcal mol^−1^. The standard parameters implemented in Discovery Studio v18 were used. A Partial Least-Square (PLS) model was built using energy grids as descriptors. The QSAR model was built using the created 3D-QSAR protocol of Discovery Studio v18.

## Conflicts of interest

There are no conflicts to declare.

## Supplementary Material

RA-010-D0RA00643B-s001
