# Supplementary material for: 6,N2-Diaryl-1,3,5-triazine-2,4-diamines: synthesis, antiproliferative activity and 3D-QSAR modeling
Source: RSC Adv. 2020 Mar 25;10(21):12135–44. doi: 10.1039/d0ra00643b (PMC9050923; doi:10.1039/d0ra00643b)
Supplement: RA-010-D0RA00643B-s001 [file RA-010-D0RA00643B-s001.pdf]

Electronic Supplementary Material (ESI) for RSC Advances.
This journal is © The Royal Society of Chemistry 2020

RSC Advances

6,N^2-Diaryl-1,3,5-triazine-2,4-diamines: Synthesis, Antiproliferative Activity and 3D-QSAR Modeling

(Electronic Supplementary Information)

Ahmad Junaid,[a] Felicia Phei Lin Lim,[a] Lay Hong Chuah,[a] and Anton V. Dolzhenko,[a,b]*

[a] School of Pharmacy, Monash University Malaysia, Jalan Lagoon Selatan, Bandar Sunway, Selangor Darul Ehsan 47500, Malaysia

[b] School of Pharmacy and Biomedical Sciences, Curtin Health Innovation Research Institute, Faculty of Health Sciences, Curtin University, GPO Box U1987 Perth, Western Australia 6845, Australia

Copies of ^{1}H and ^{13}C NMR spectra for the prepared new 6,N^2-diaryl-1,3,5-triazine-2,4-diamines (**2**, **3**, **4**, **30**, **34**, **53**, **57**, **59**, **65**, **71**, **84**, **91**, **111**, **116**, **122**, and **123**)

N^2-(2-fluorophenyl)-6-phenyl-1,3,5-triazine-2,4-diamine (2)

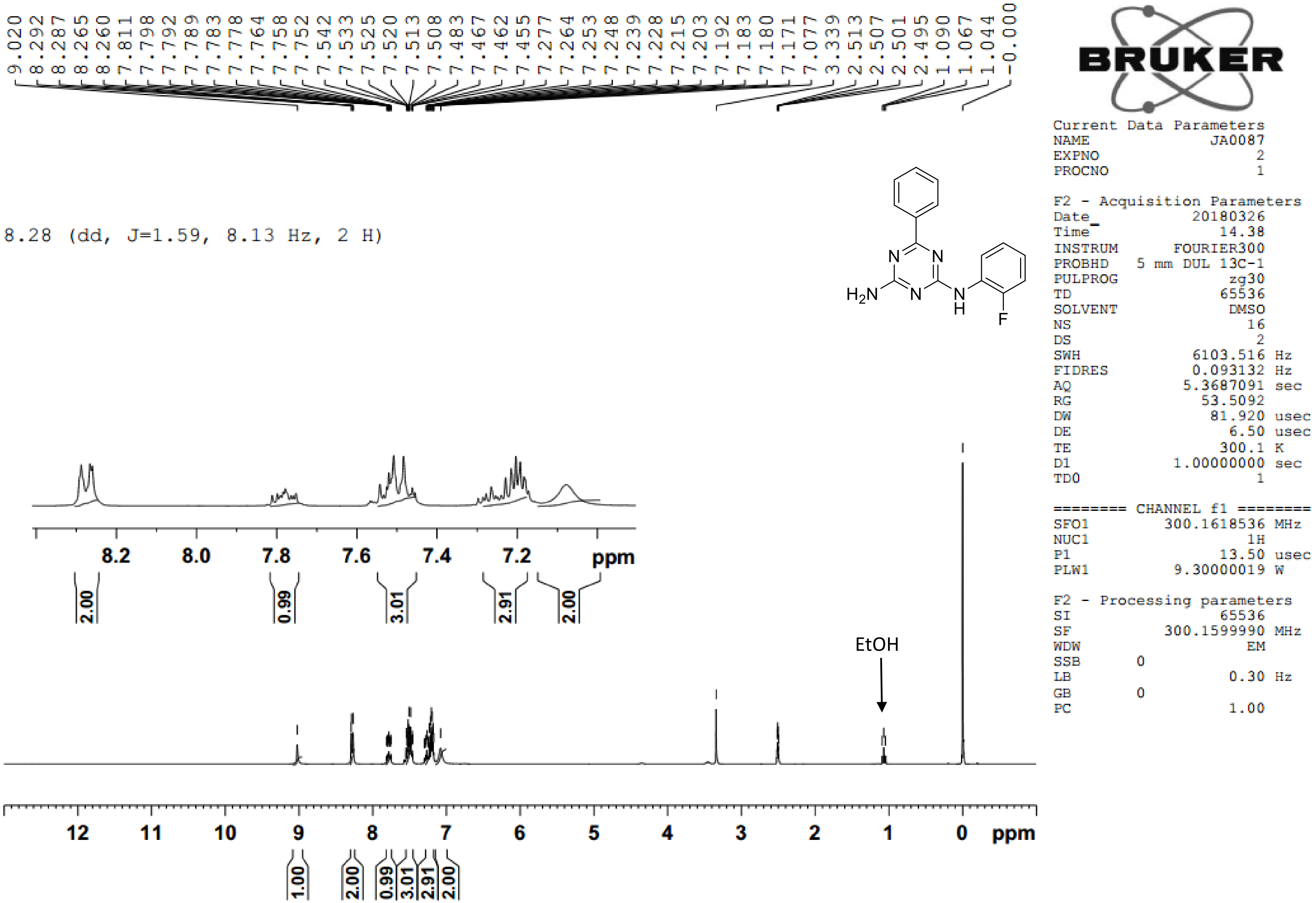

N^2-(2-fluorophenyl)-6-phenyl-1,3,5-triazine-2,4-diamine (2)

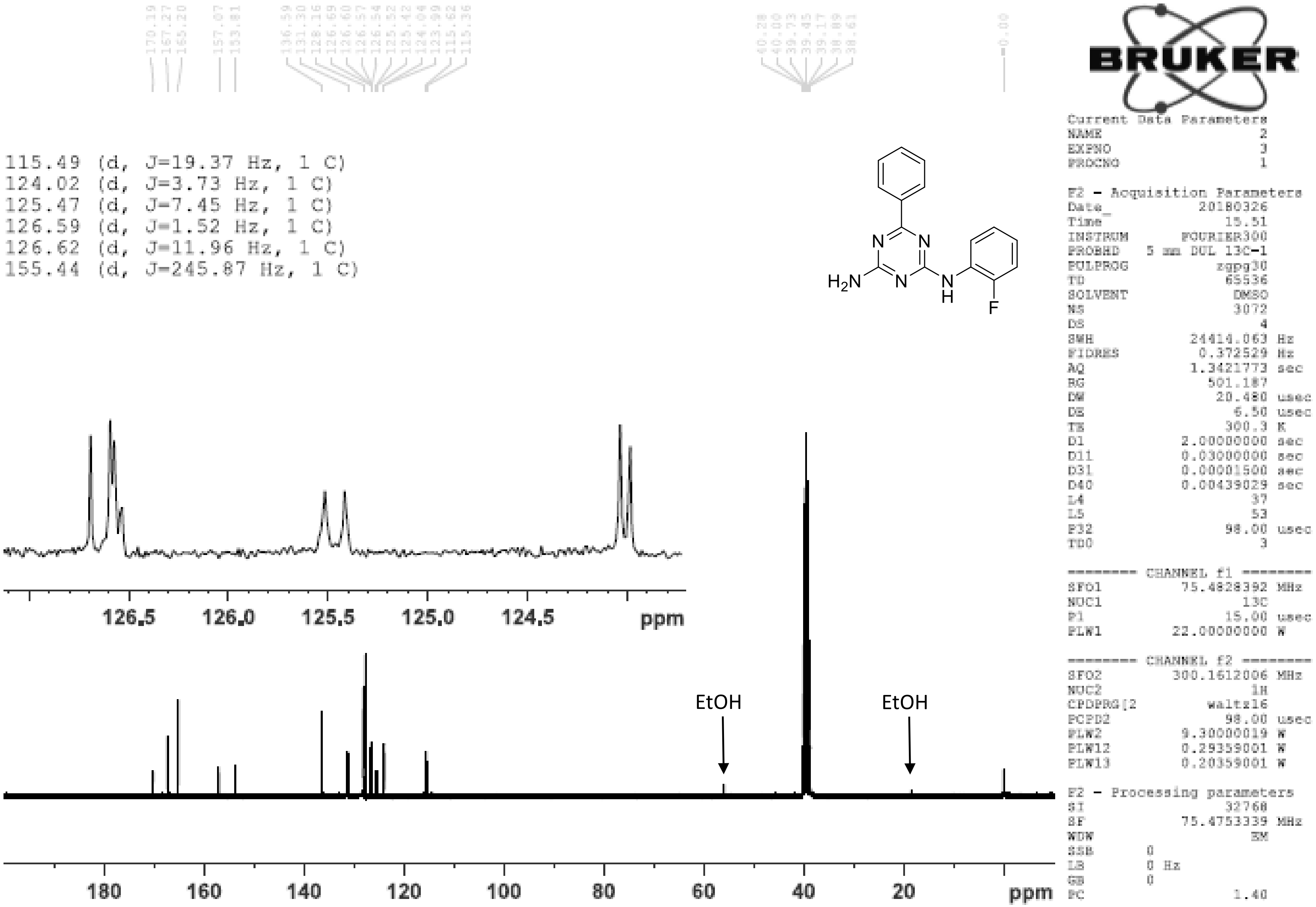

N^2-(4-fluorophenyl)-6-phenyl-1,3,5-triazine-2,4-diamine (3)

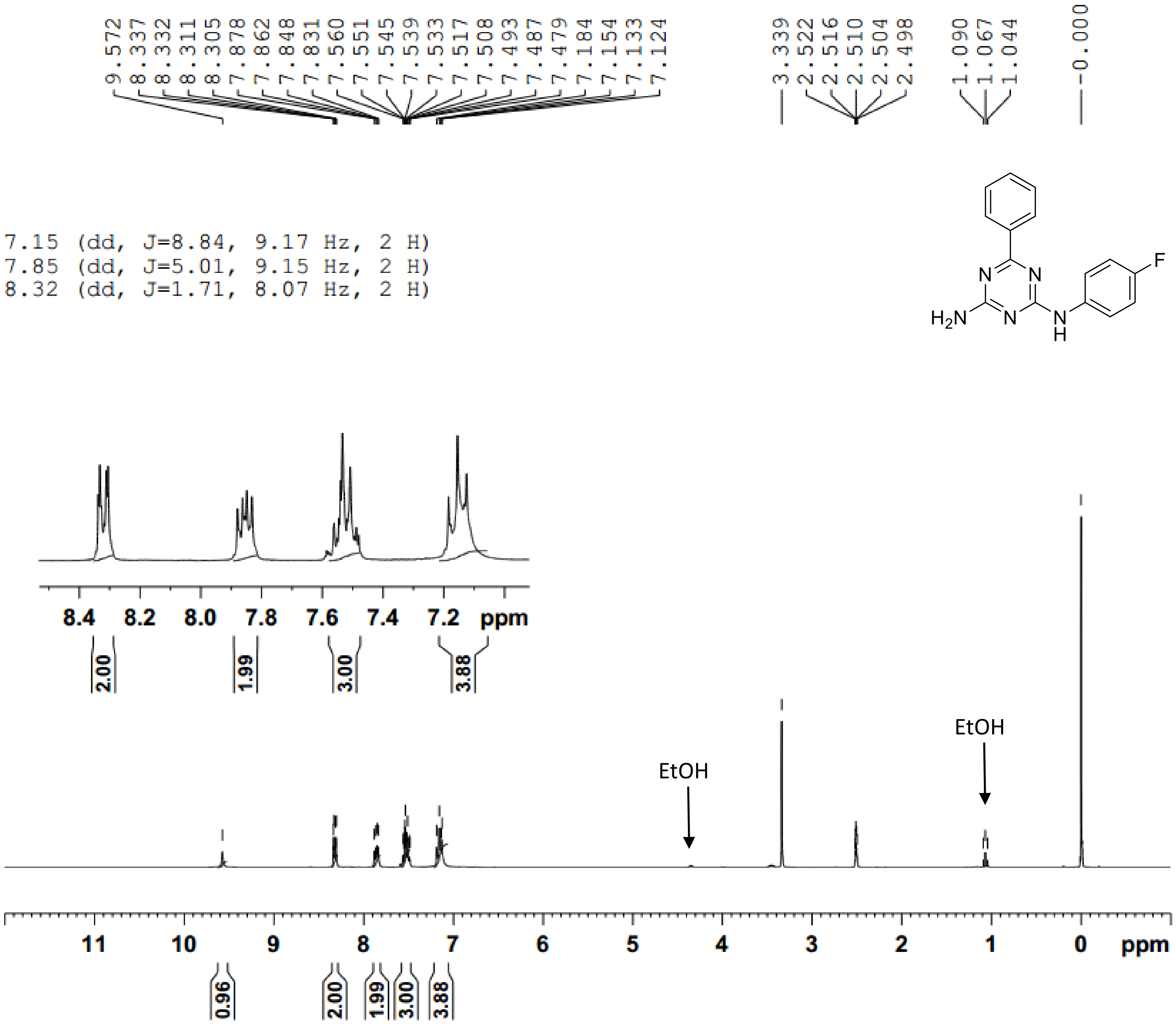


```
Current Data Parameters
NAME              JA0027
EXPNO                  2
PROCNO                 1

F2 - Acquisition Parameters
Date_           20180326
Time               14.43
INSTRUM      FOURIER300
PROBHD   5 mm DUL 13C-1
PULPROG             zg30
TD                 65536
SOLVENT             DMSO
NS                    16
DS                     2
SWH             6103.516 Hz
FIDRES          0.093132 Hz
AQ             5.3687091 sec
RG               68.2444
DW                81.920 usec
DE                  6.50 usec
TE                 300.1 K
D1            1.00000000 sec
TD0                    1

======== CHANNEL f1 ========
SFO1         300.1618536 MHz
NUC1                  1H
P1                 13.50 usec
PLW1          9.30000019 W

F2 - Processing parameters
SI                 65536
SF           300.1599981 MHz
WDW                   EM
SSB       0
LB                  0.30 Hz
GB        0
PC                  1.00
```

N^2-(4-fluorophenyl)-6-phenyl-1,3,5-triazine-2,4-diamine (3)

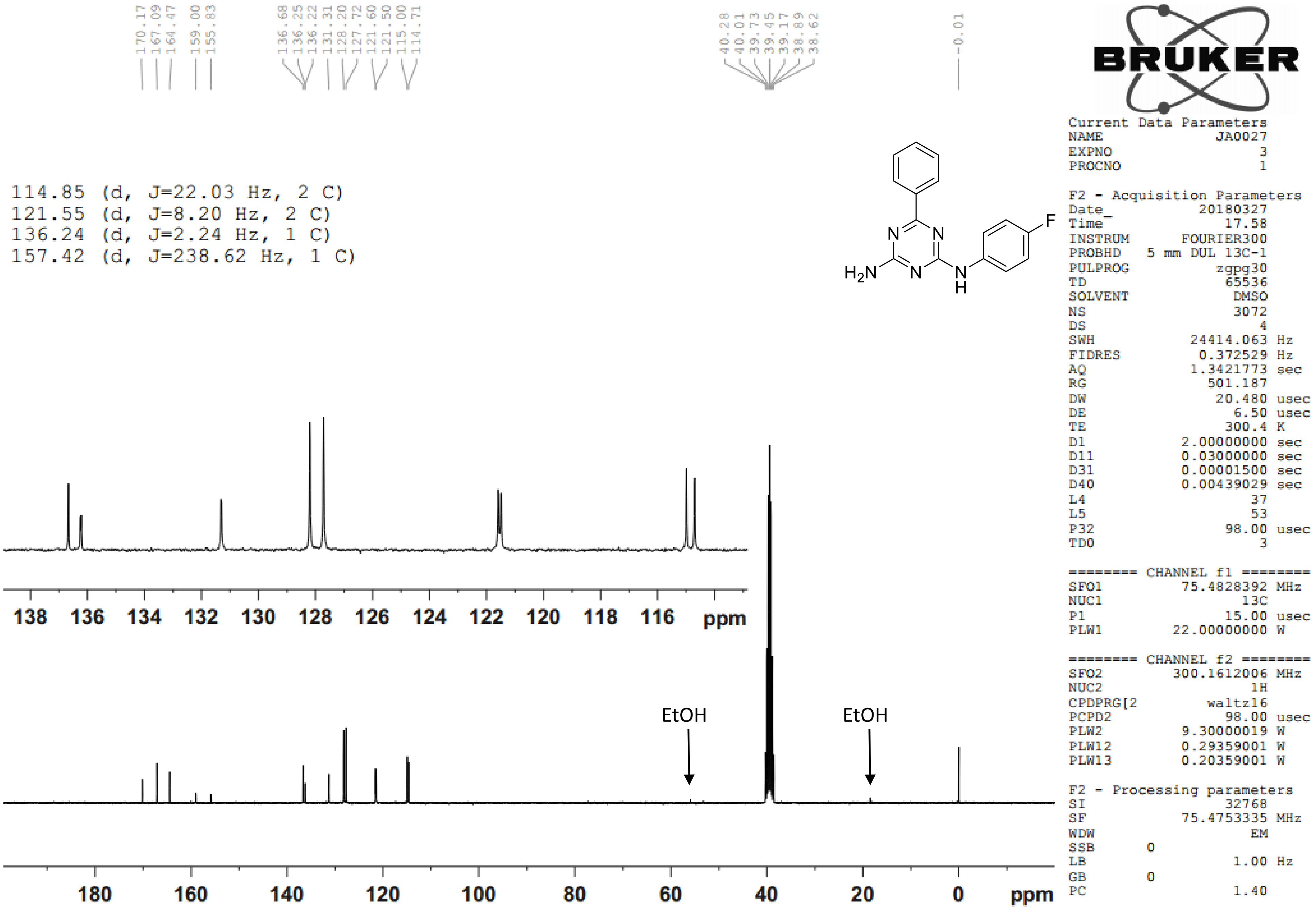

N²-(2-chlorophenyl)-6-phenyl-1,3,5-triazine-2,4-diamine (4)

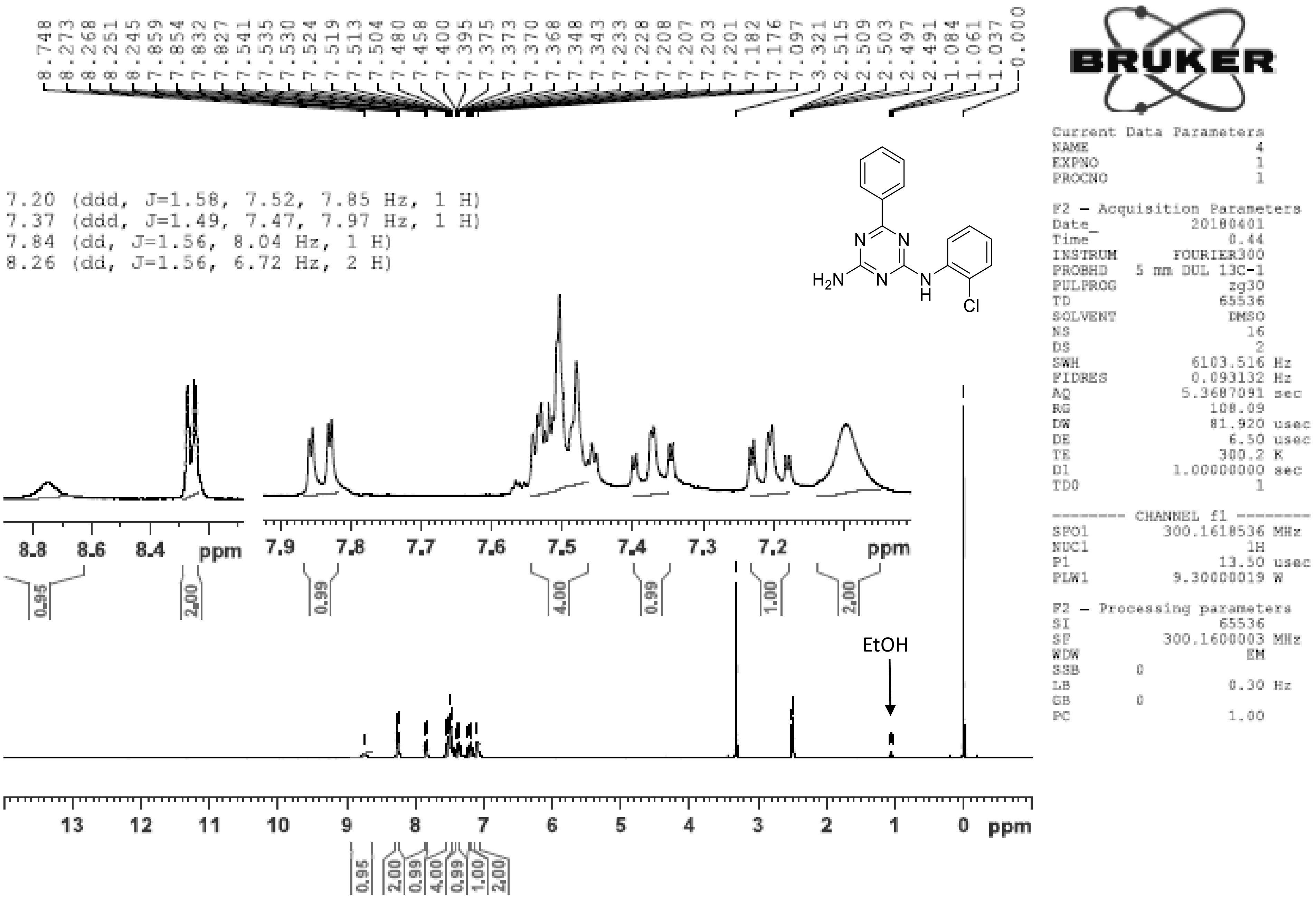

N[2]-(2-chlorophenyl)-6-phenyl-1,3,5-triazine-2,4-diamine (4)

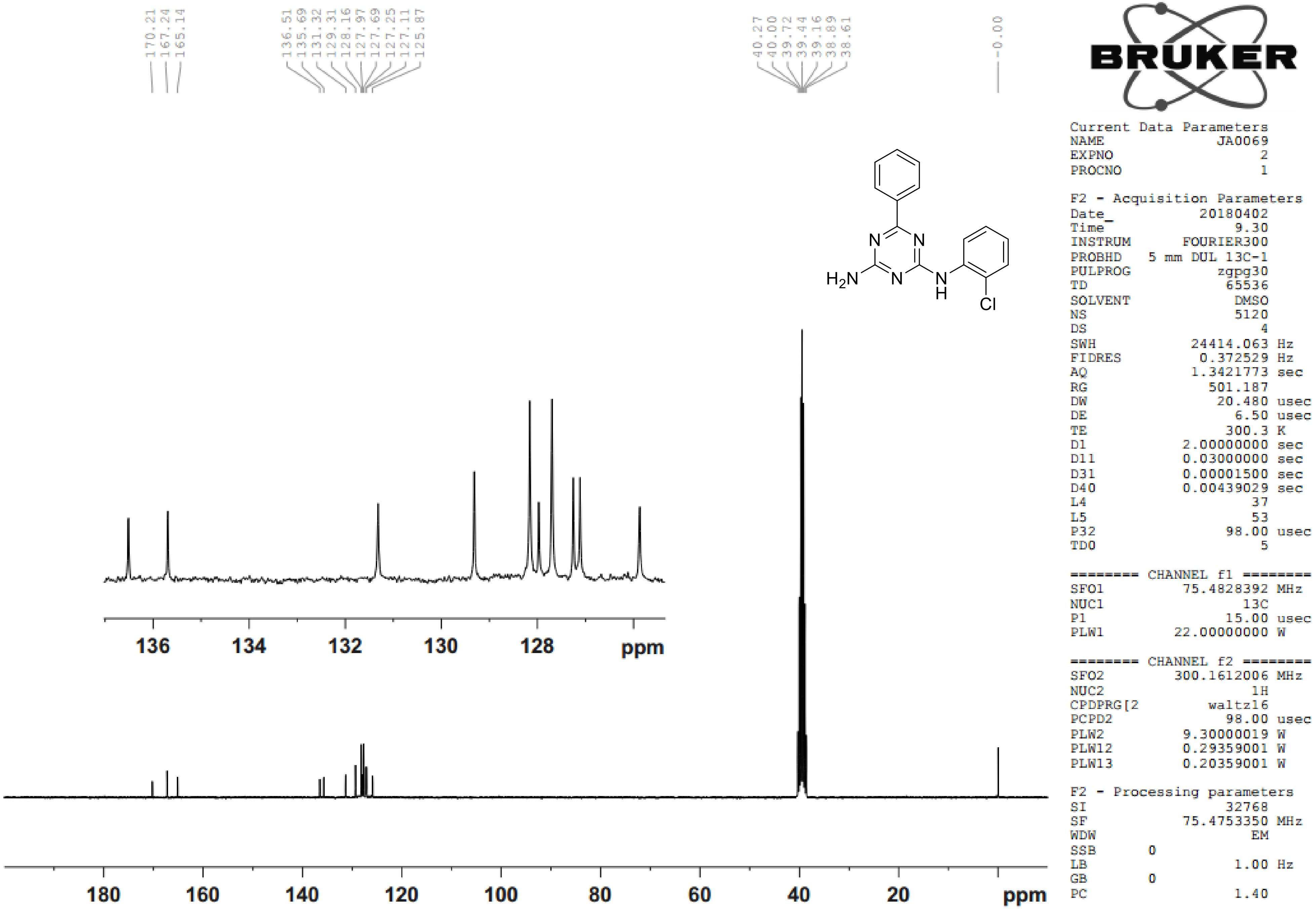

6-(4-fluorophenyl)-N²-(4-methoxyphenyl)-1,3,5-triazine-2,4-diamine (30)

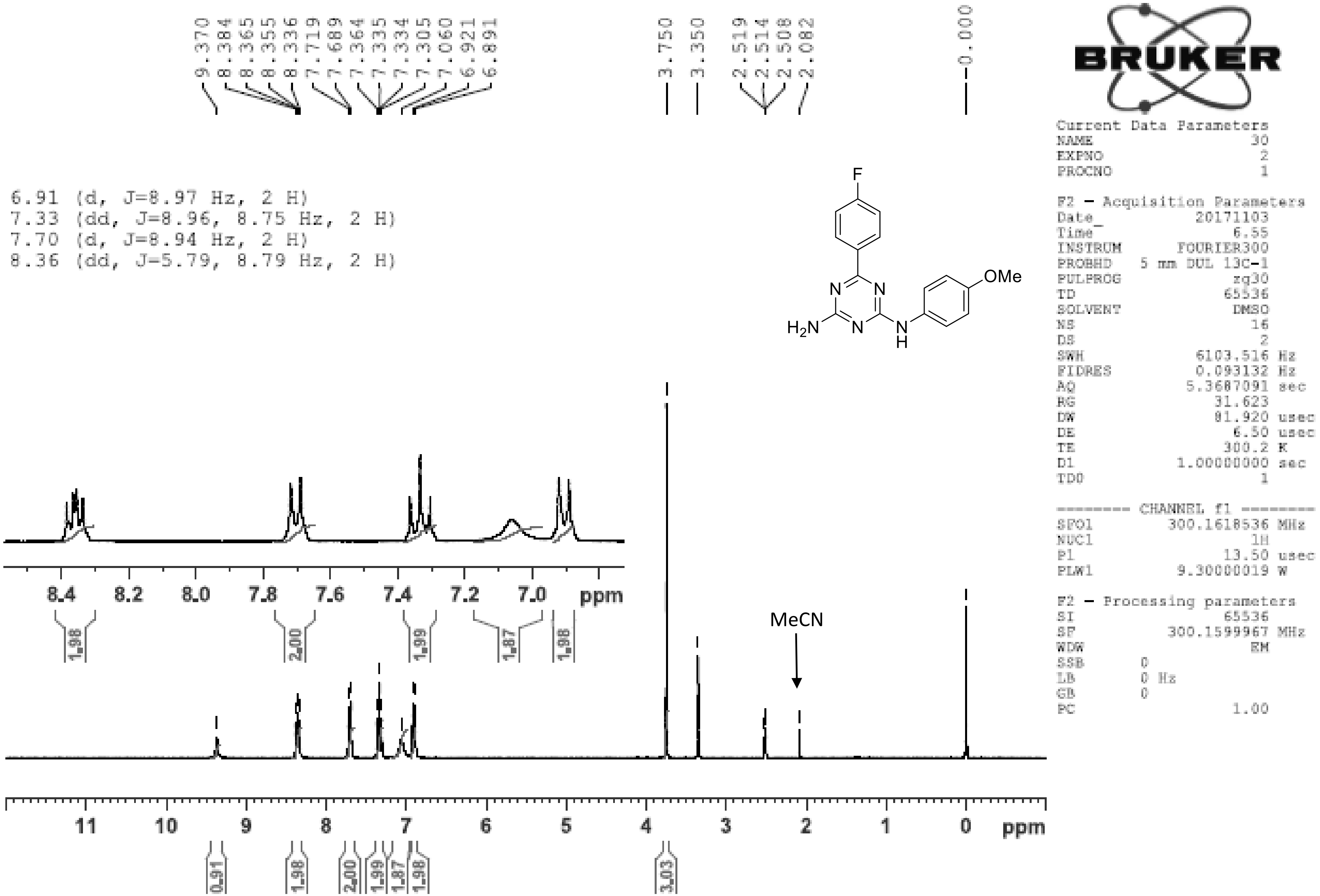

6-(4-fluorophenyl)-N^2-(4-methoxyphenyl)-1,3,5-triazine-2,4-diamine (30)

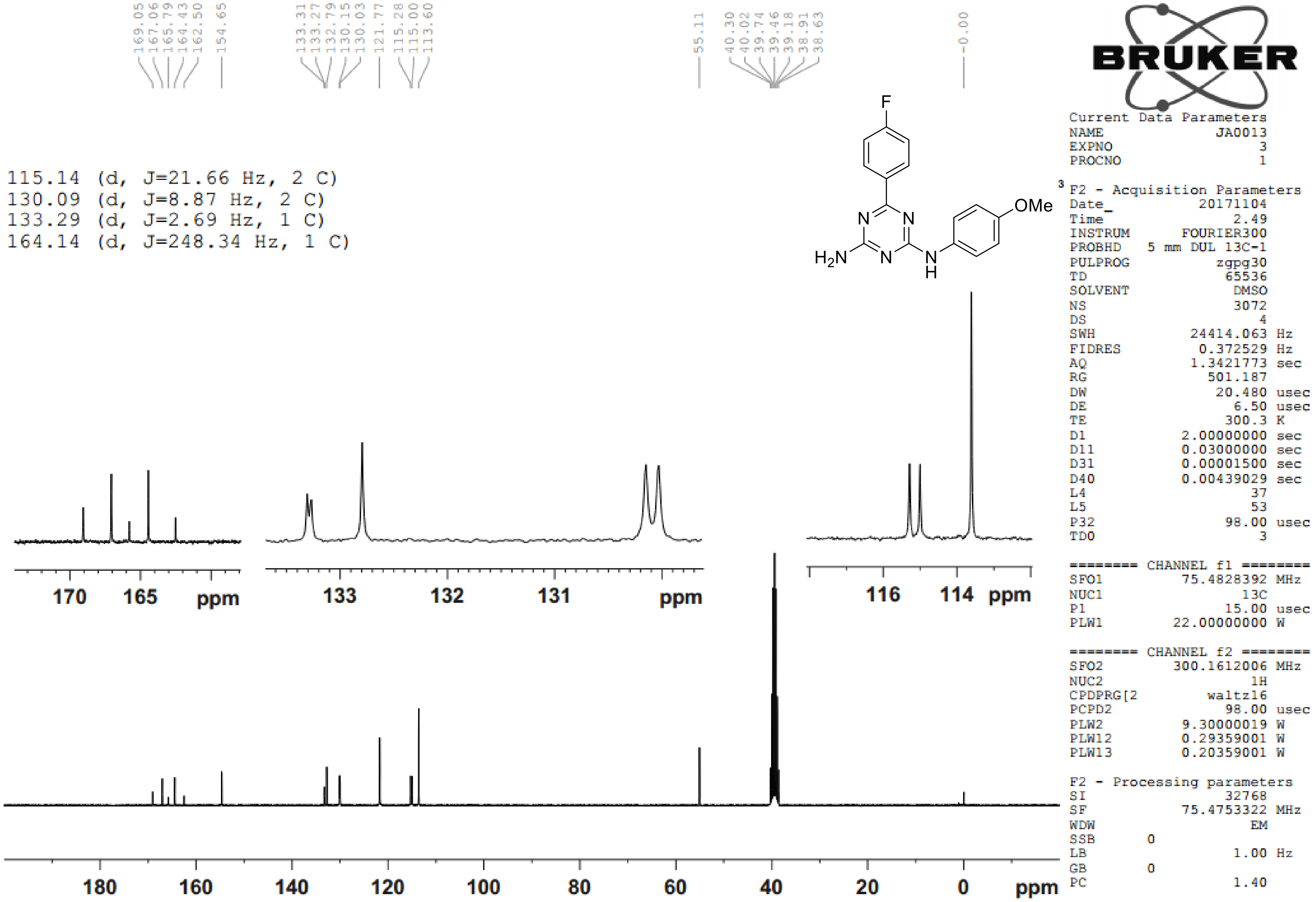

6-(4-chlorophenyl)-N^2-phenyl-1,3,5-triazine-2,4-diamine (34)

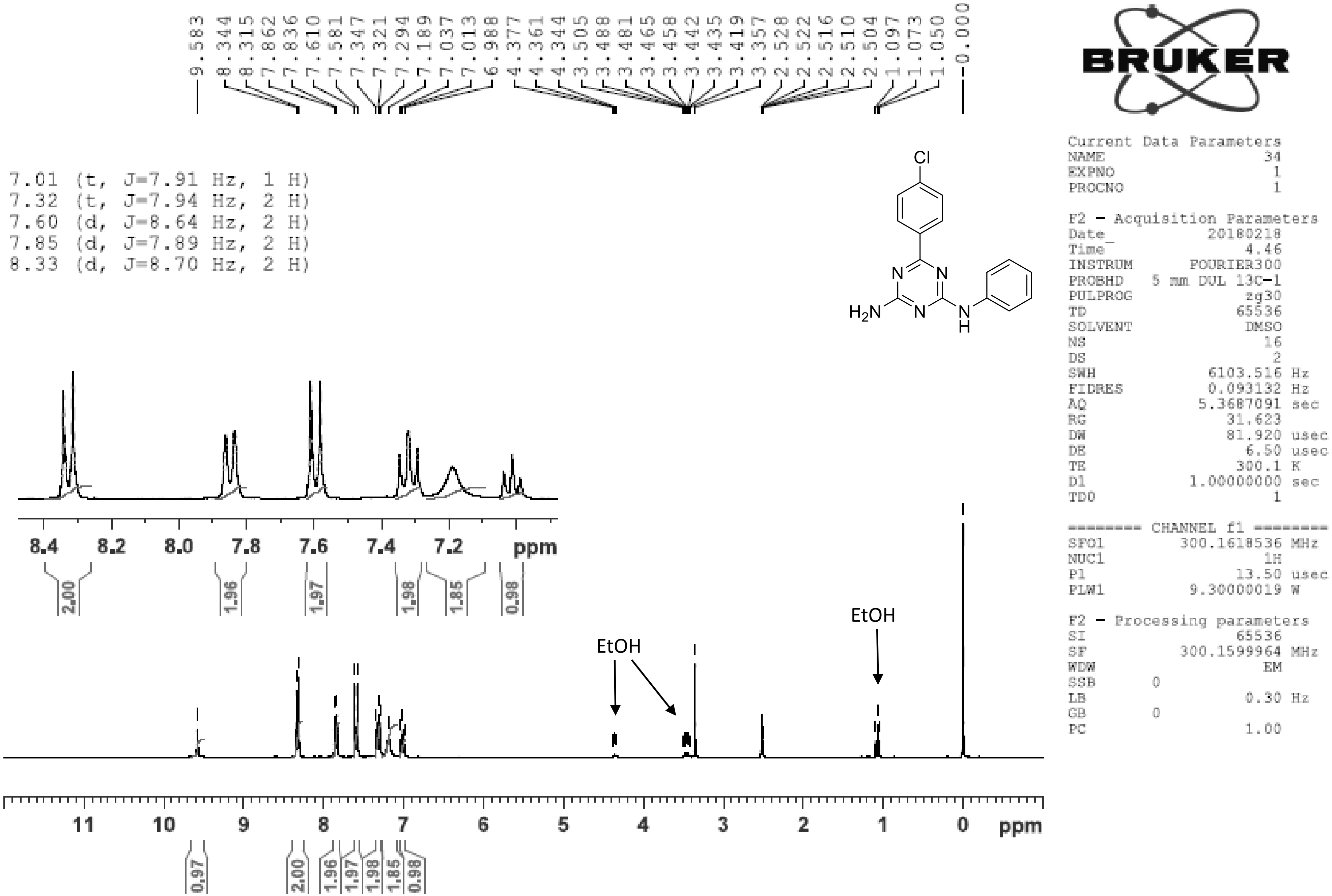

6-(4-chlorophenyl)-N²-phenyl-1,3,5-triazine-2,4-diamine (34)

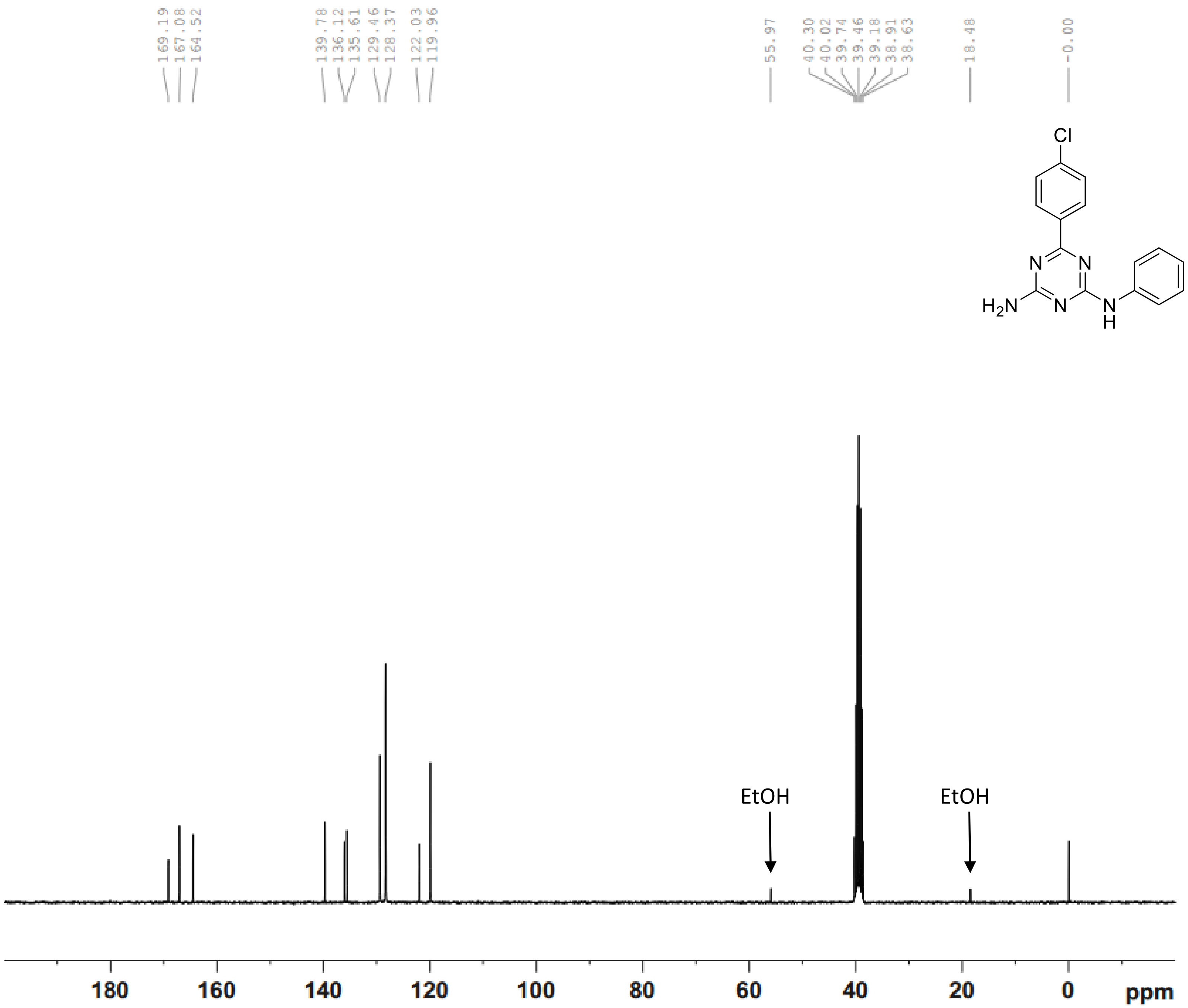


```
Current Data Parameters
NAME              JA0210
EXPNO                  2
PROCNO                 1

F2 - Acquisition Parameters
Date_           20180218
Time               23.57
INSTRUM      FOURIER300
PROBHD   5 mm DUL 13C-1
PULPROG          zgpg30
TD                 65536
SOLVENT             DMSO
NS                  3072
DS                     4
SWH           24414.063 Hz
FIDRES         0.372529 Hz
AQ            1.3421773 sec
RG              501.187
DW               20.480 usec
DE                 6.50 usec
TE                300.3 K
D1           2.00000000 sec
D11          0.03000000 sec
D31          0.00001500 sec
D40          0.00439029 sec
L4                   37
L5                   53
P32               98.00 usec
TD0                   3

======== CHANNEL f1 ========
SFO1        75.4828392 MHz
NUC1               13C
P1               15.00 usec
PLW1       22.00000000 W

======== CHANNEL f2 ========
SFO2       300.1612006 MHz
NUC2                1H
CPDPRG[2        waltz16
PCPD2            98.00 usec
PLW2        9.30000019 W
PLW12       0.29359001 W
PLW13       0.20359001 W

F2 - Processing parameters
SI                32768
SF           75.4753327 MHz
WDW                  EM
SSB        0
LB                 1.00 Hz
GB         0
PC                 1.40
```

N^2-(4-fluorophenyl)-6-(m-tolyl)-1,3,5-triazine-2,4-diamine (53)

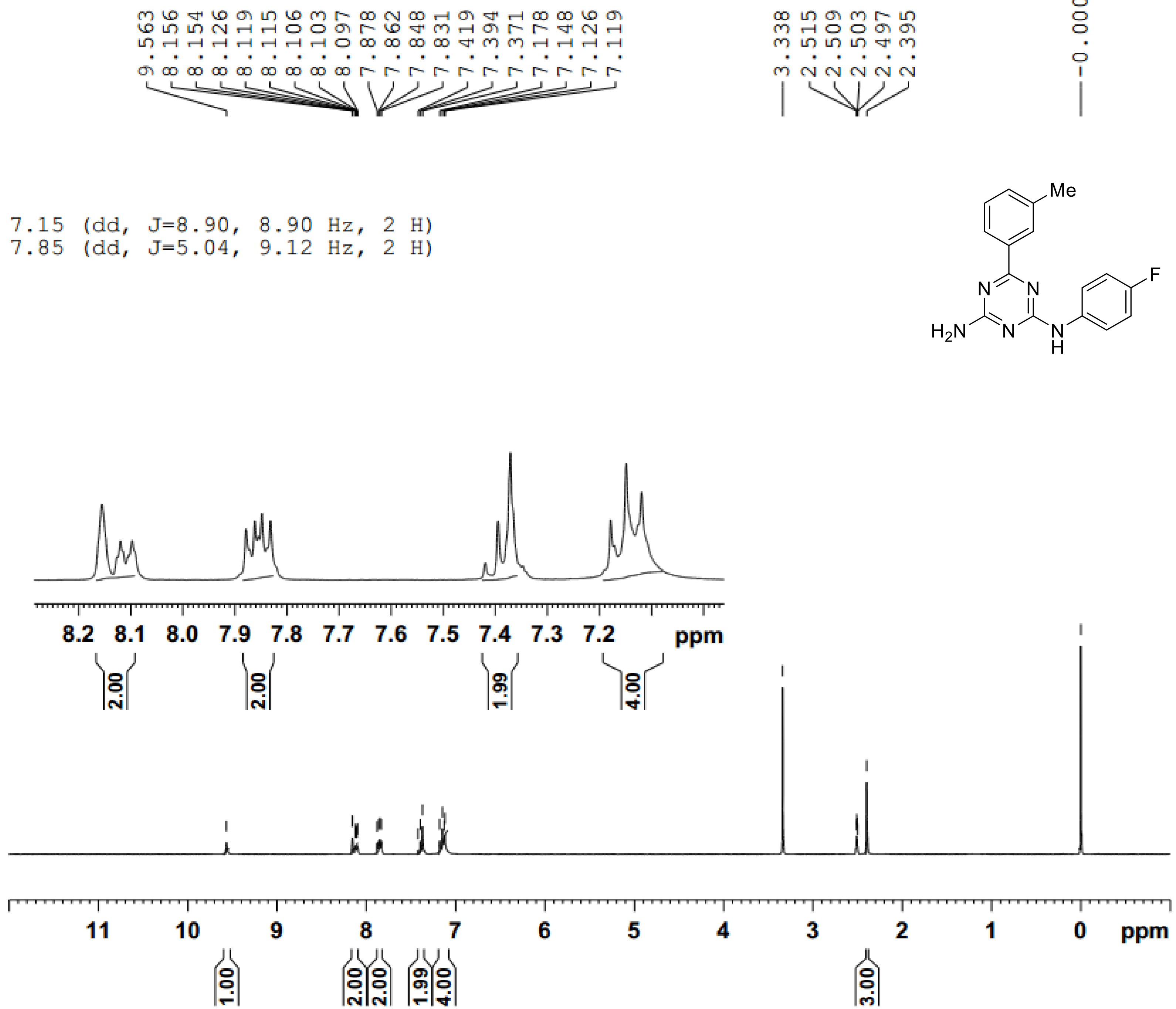


```
Current Data Parameters
NAME              JA0503
EXPNO                  3
PROCNO                 1

F2 - Acquisition Parameters
Date_           20180913
Time                9.45
INSTRUM       FOURIER300
PROBHD   5 mm DUL 13C-1
PULPROG             zg30
TD                 65536
SOLVENT             DMSO
NS                    16
DS                     2
SWH             6103.516 Hz
FIDRES          0.093132 Hz
AQ             5.3687091 sec
RG               67.1476
DW                81.920 usec
DE                  6.50 usec
TE                 300.2 K
D1            1.00000000 sec
TD0                    1

======== CHANNEL f1 ========
SFO1         300.1618536 MHz
NUC1                  1H
P1                 13.50 usec
PLW1          9.30000019 W

F2 - Processing parameters
SI                 65536
SF           300.1599984 MHz
WDW                   EM
SSB      0
LB                  0.30 Hz
GB       0
PC                  1.00
```

N^2-(4-fluorophenyl)-6-(m-tolyl)-1,3,5-triazine-2,4-diamine (53)

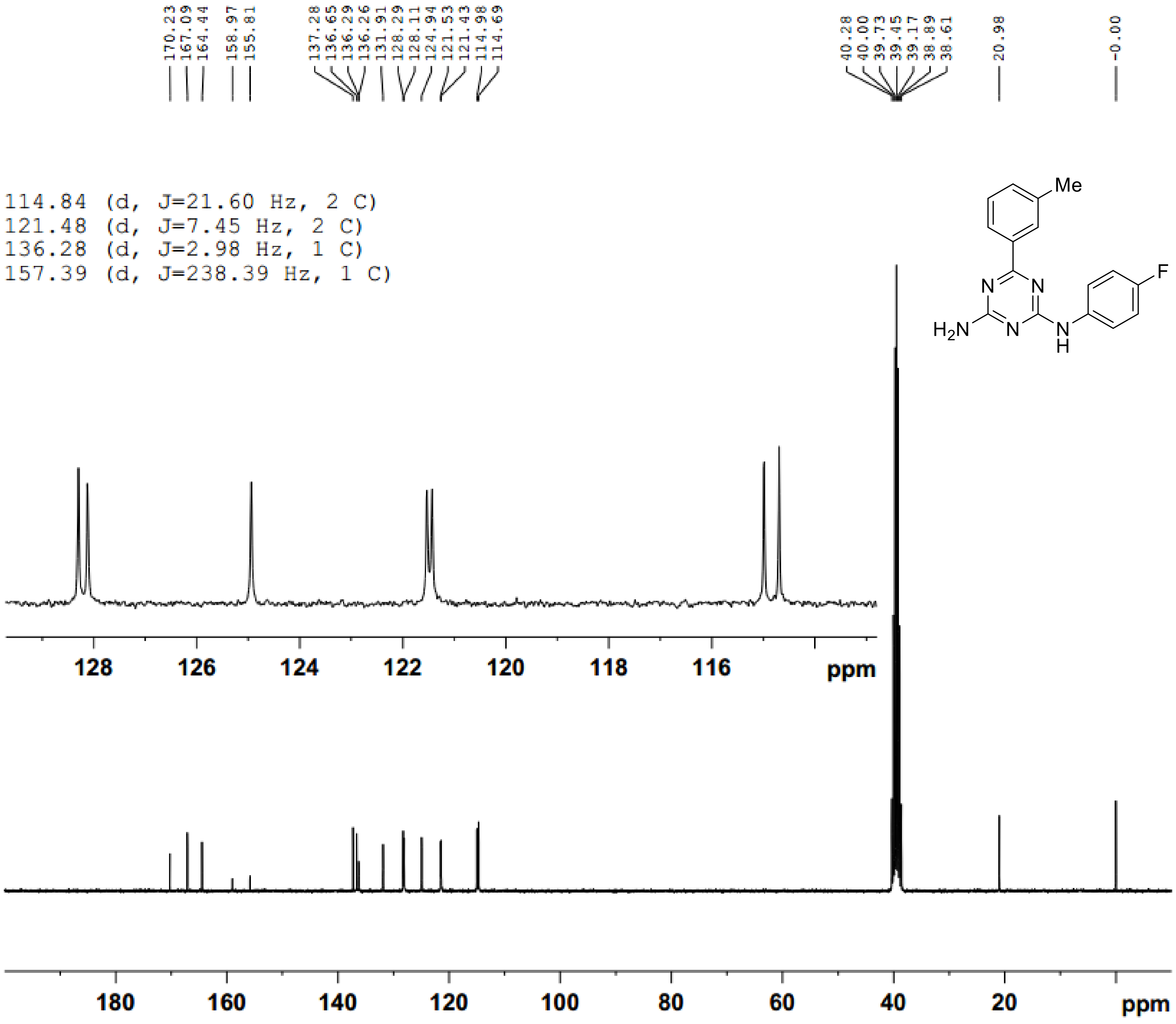

Current Data Parameters
NAME JA0503
EXPNO 4
PROCNO 1

F2 - Acquisition Parameters
Date_ 20180913
Time 22.43
INSTRUM FOURIER300
PROBHD 5 mm DUL 13C-1
PULPROG zgpg30
TD 65536
SOLVENT DMSO
NS 4096
DS 4
SWH 24414.063 Hz
FIDRES 0.372529 Hz
AQ 1.3421773 sec
RG 501.187
DW 20.480 usec
DE 6.50 usec
TE 300.3 K
D1 2.00000000 sec
D11 0.03000000 sec
D31 0.00001500 sec
D40 0.00439029 sec
L4 37
L5 53
P32 98.00 usec
TD0 4

======== CHANNEL f1 ========
SFO1 75.4828392 MHz
NUC1 13C
P1 15.00 usec
PLW1 22.00000000 W

======== CHANNEL f2 ========
SFO2 300.1612006 MHz
NUC2 1H
CPDPRG[2 waltz16
PCPD2 98.00 usec
PLW2 9.30000019 W
PLW12 0.29359001 W
PLW13 0.20359001 W

F2 - Processing parameters
SI 32768
SF 75.4753335 MHz
WDW EM
SSB 0
LB 1.00 Hz
GB 0
PC 1.40

N^2-(4-methoxyphenyl)-6-(m-tolyl)-1,3,5-triazine-2,4-diamine (57)

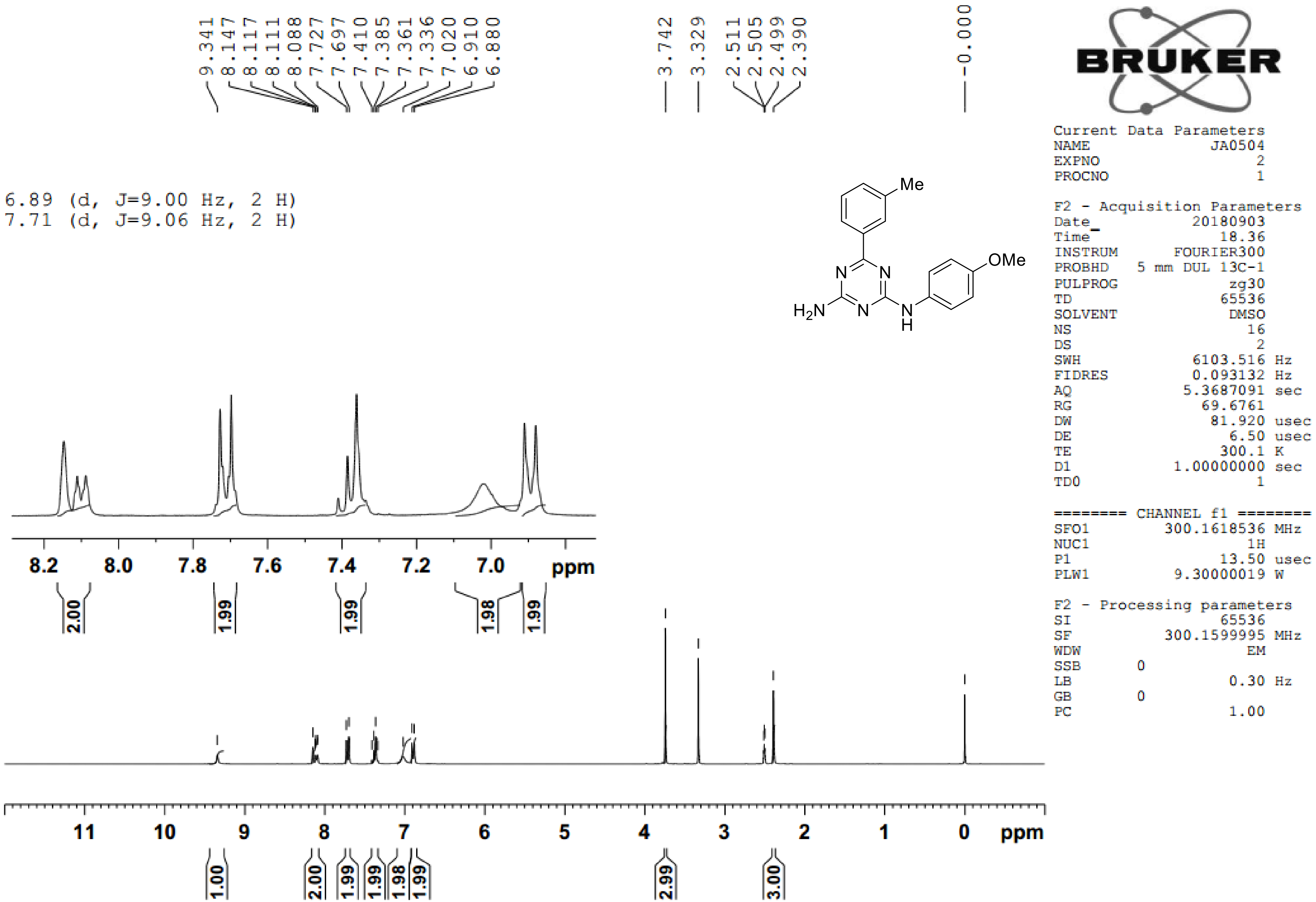

N²-(4-methoxyphenyl)-6-(m-tolyl)-1,3,5-triazine-2,4-diamine (57)

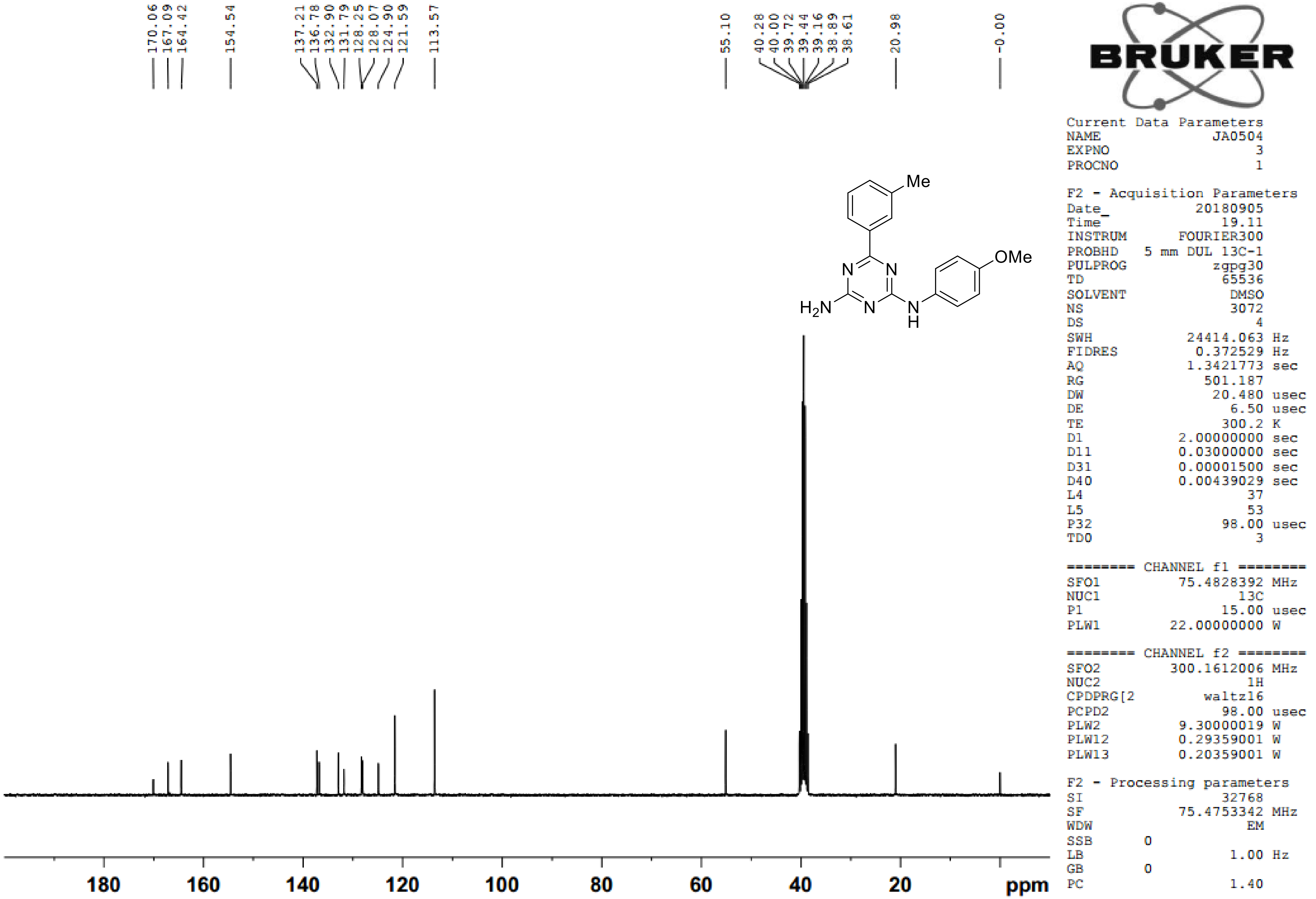

N^2-(4-isopropylphenyl)-6-(m-tolyl)-1,3,5-triazine-2,4-diamine (59)

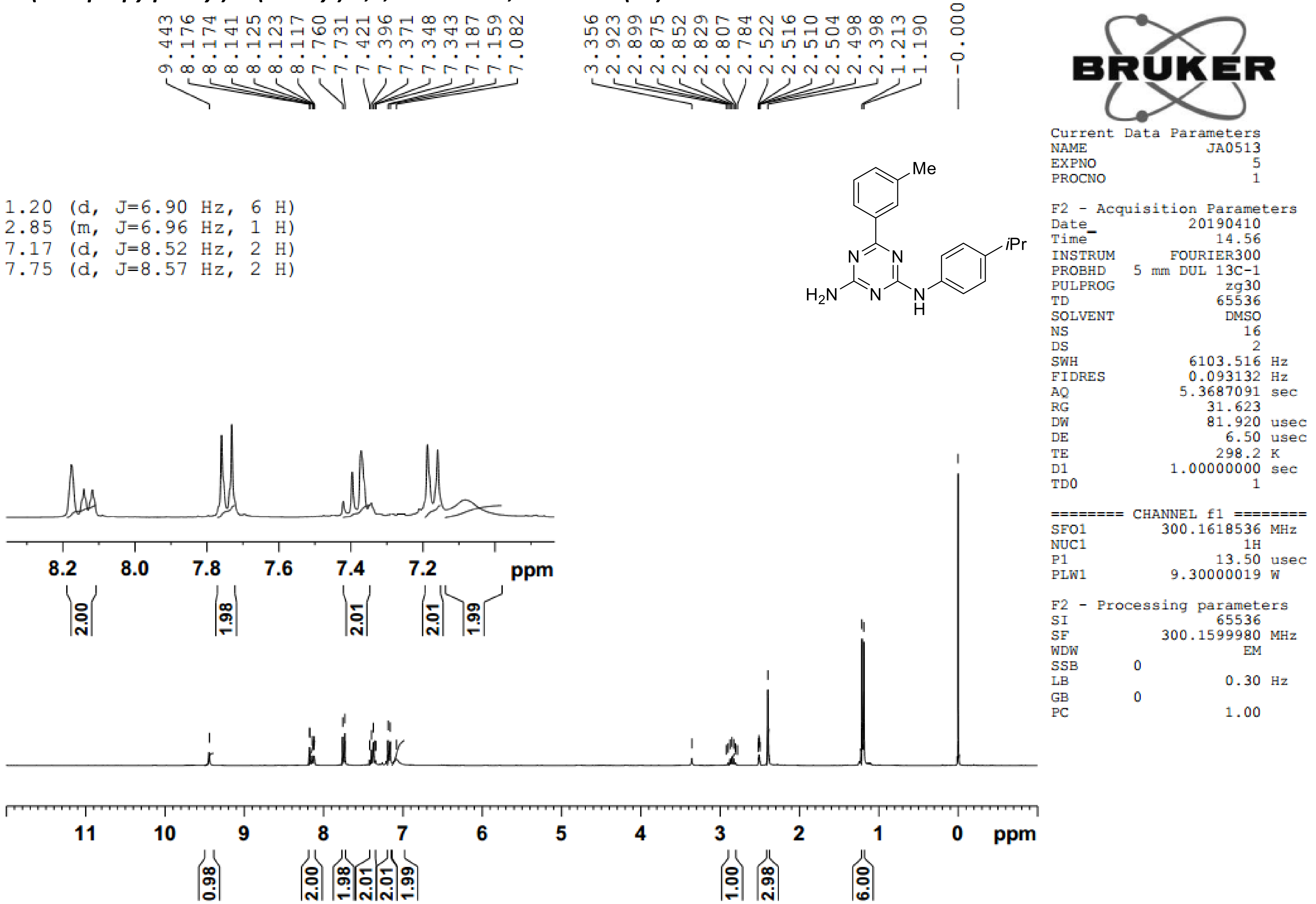

N[2]-(4-isopropylphenyl)-6-(m-tolyl)-1,3,5-triazine-2,4-diamine (59)

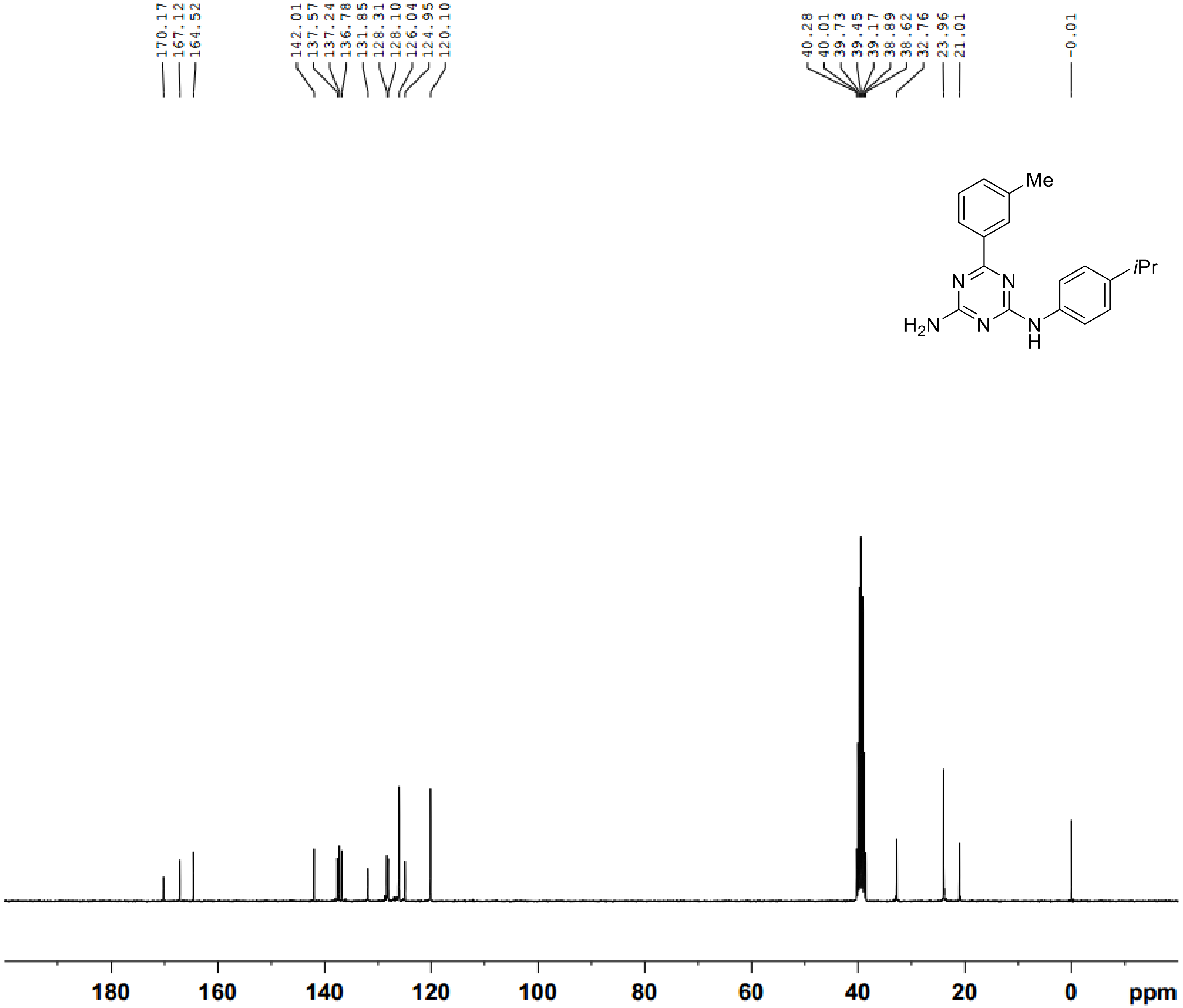

Current Data Parameters
NAME JA0513
EXPNO 6
PROCNO 1

F2 - Acquisition Parameters
Date_ 20190410
Time 19.30
INSTRUM FOURIER300
PROBHD 5 mm DUL 13C-1
PULPROG zgpg30
TD 65536
SOLVENT DMSO
NS 3072
DS 4
SWH 24414.063 Hz
FIDRES 0.372529 Hz
AQ 1.3421773 sec
RG 501.187
DW 20.480 usec
DE 6.50 usec
TE 298.3 K
D1 2.00000000 sec
D11 0.03000000 sec
D31 0.00001500 sec
D40 0.00439029 sec
L4 37
L5 53
P32 98.00 usec
TD0 3

======== CHANNEL f1 ========
SFO1 75.4828392 MHz
NUC1 13C
P1 15.00 usec
PLW1 22.00000000 W

======== CHANNEL f2 ========
SFO2 300.1612006 MHz
NUC2 1H
CPDPRG[2 waltz16
PCPD2 98.00 usec
PLW2 9.30000019 W
PLW12 0.29359001 W
PLW13 0.20359001 W

F2 - Processing parameters
SI 32768
SF 75.4753327 MHz
WDW EM
SSB 0
LB 1.00 Hz
GB 0
PC 1.40

N²-(4-methoxyphenyl)-6-(p-tolyl)-1,3,5-triazine-2,4-diamine (65)

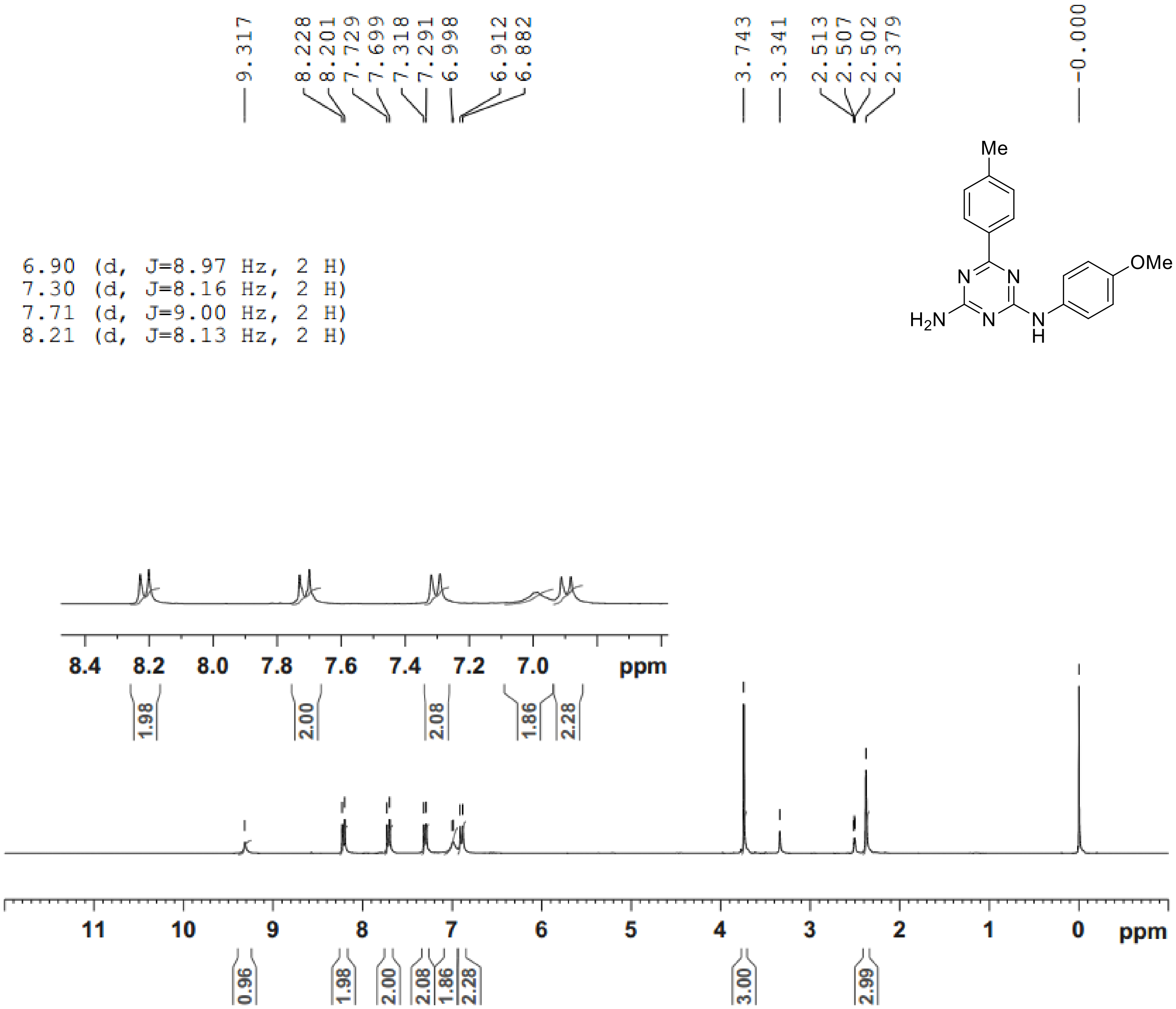

6.90 (d, J=8.97 Hz, 2 H)
7.30 (d, J=8.16 Hz, 2 H)
7.71 (d, J=9.00 Hz, 2 H)
8.21 (d, J=8.13 Hz, 2 H)

BRUKER

```
Current Data Parameters
NAME                JA0007
EXPNO                    2
PROCNO                   1

F2 - Acquisition Parameters
Date_             20171030
Time                 14.39
INSTRUM         FOURIER300
PROBHD    5 mm DUL 13C-1
PULPROG               zg30
TD                   65536
SOLVENT               DMSO
NS                      16
DS                       2
SWH            6103.516 Hz
FIDRES         0.093132 Hz
AQ            5.3687091 sec
RG                  31.623
DW              81.920 usec
DE                6.50 usec
TE                 300.0 K
D1           1.00000000 sec
TD0                      1

======== CHANNEL f1 ========
SFO1        300.1618536 MHz
NUC1                    1H
P1               13.50 usec
PLW1          9.30000019 W

F2 - Processing parameters
SI                   65536
SF          300.1599989 MHz
WDW                     EM
SSB       0
LB                 0.30 Hz
GB        0
PC                    1.00
```

N[2]-(4-methoxyphenyl)-6-(p-tolyl)-1,3,5-triazine-2,4-diamine (65)

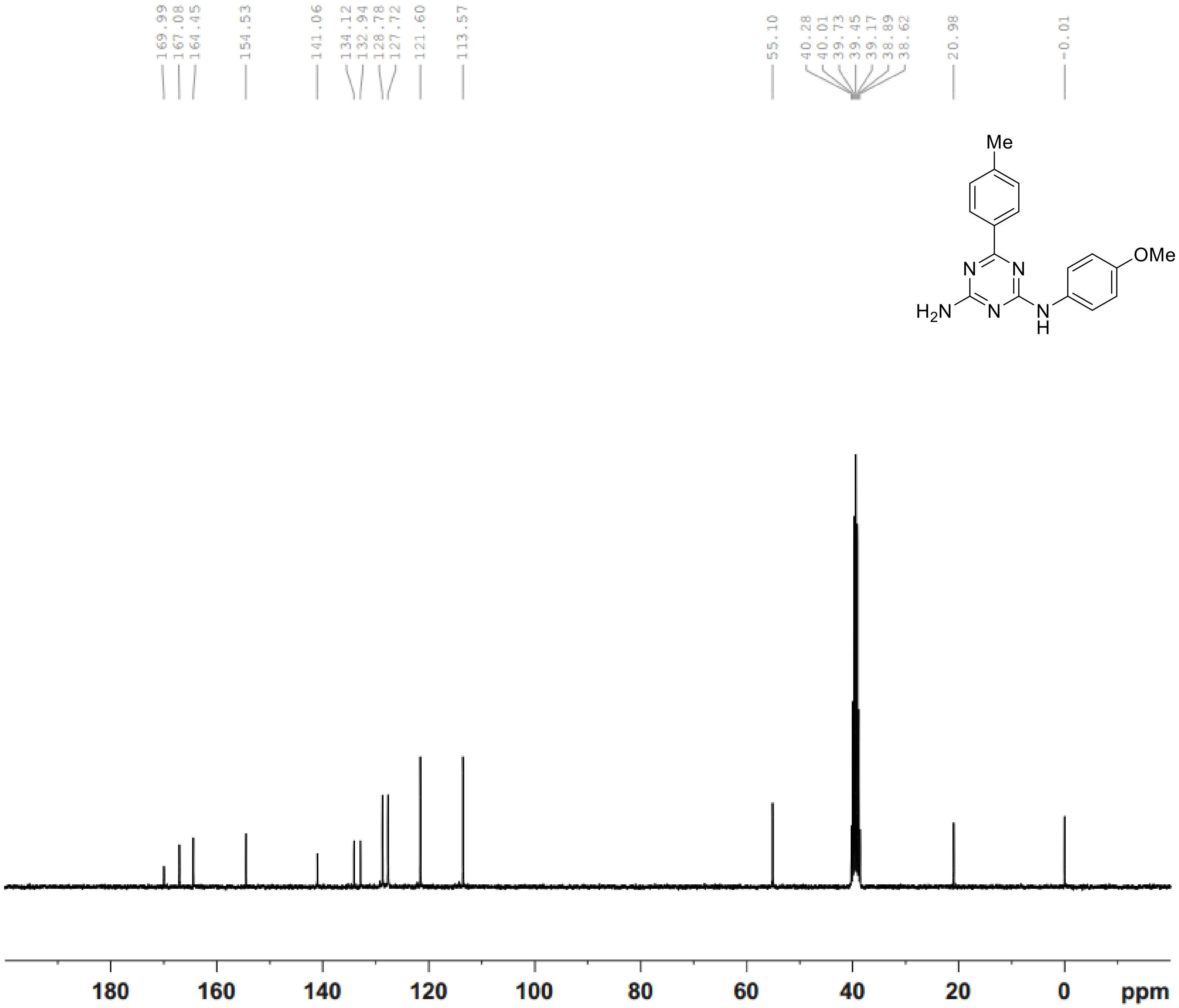

BRUKER

```
Current Data Parameters
NAME              JA0007
EXPNO                  3
PROCNO                 1

F2 - Acquisition Parameters
Date_           20171031
Time                5.08
INSTRUM       FOURIER300
PROBHD   5 mm DUL 13C-1
PULPROG          zgpg30
TD                 65536
SOLVENT             DMSO
NS                  1024
DS                     4
SWH            24414.063 Hz
FIDRES          0.372529 Hz
AQ             1.3421773 sec
RG               501.187
DW                20.480 usec
DE                  6.50 usec
TE                 300.1 K
D1            2.00000000 sec
D11           0.03000000 sec
D31           0.00001500 sec
D40           0.00439029 sec
L4                    37
L5                    53
P32                98.00 usec
TD0                    1

======== CHANNEL f1 ========
SFO1          75.4828392 MHz
NUC1                 13C
P1                 15.00 usec
PLW1         22.00000000 W

======== CHANNEL f2 ========
SFO2         300.1612006 MHz
NUC2                  1H
CPDPRG[2         waltz16
PCPD2              98.00 usec
PLW2          9.30000019 W
PLW12         0.29359001 W
PLW13         0.20359001 W

F2 - Processing parameters
SI                 32768
SF            75.4753335 MHz
WDW                   EM
SSB      0
LB                  1.00 Hz
GB       0
PC                  1.40
```

N²,6-bis(4-methoxyphenyl)-1,3,5-triazine-2,4-diamine (71)

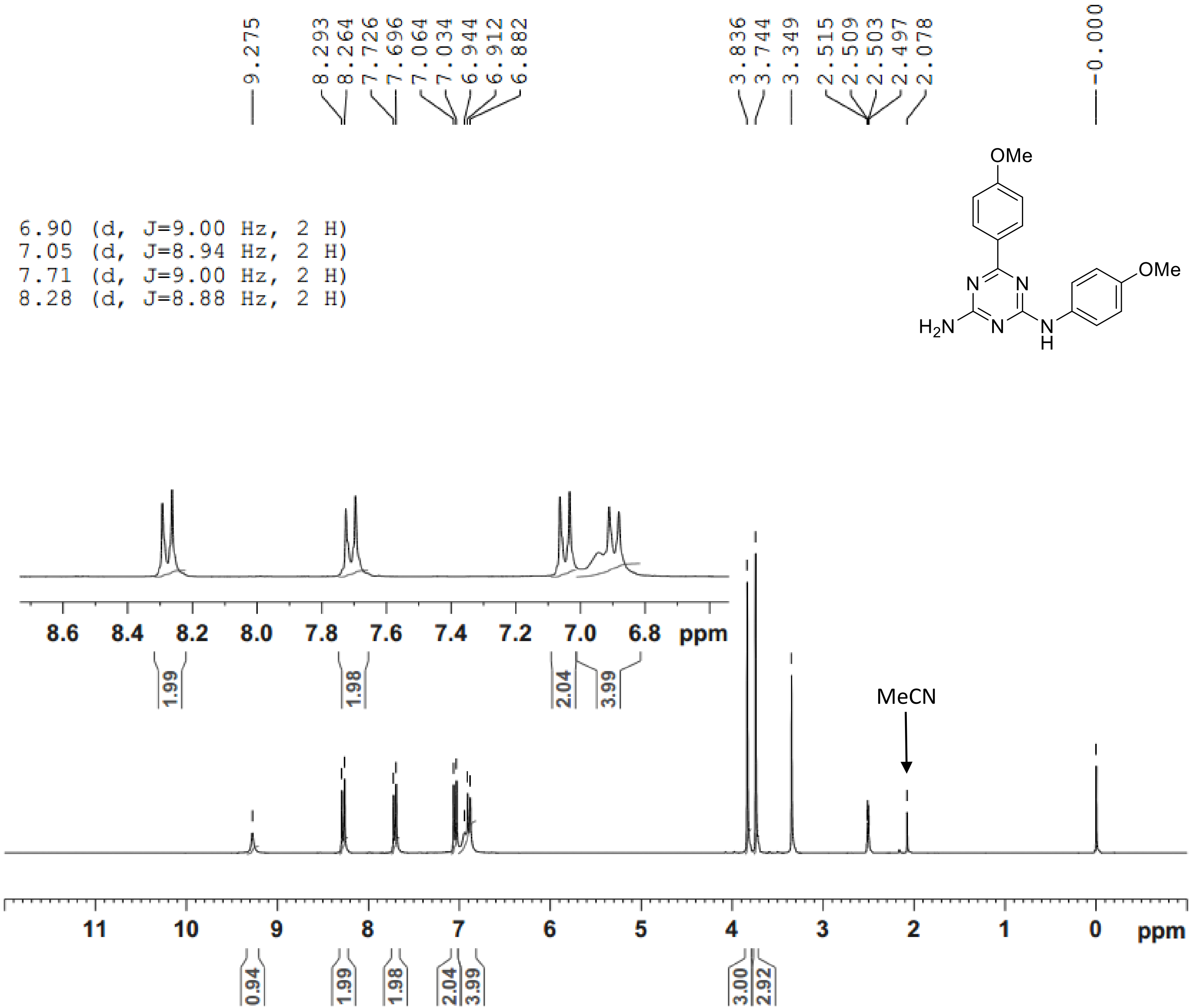


```
Current Data Parameters
NAME              JA0004
EXPNO                  2
PROCNO                 1

F2 - Acquisition Parameters
Date_           20171029
Time               15.31
INSTRUM      FOURIER300
PROBHD    5 mm DUL 13C-1
PULPROG             zg30
TD                 65536
SOLVENT             DMSO
NS                    16
DS                     2
SWH             6103.516 Hz
FIDRES          0.093132 Hz
AQ             5.3687091 sec
RG                31.623
DW                81.920 usec
DE                  6.50 usec
TE                 300.0 K
D1            1.00000000 sec
TD0                    1

======== CHANNEL f1 ========
SFO1         300.1618536 MHz
NUC1                  1H
P1                 13.50 usec
PLW1          9.30000019 W

F2 - Processing parameters
SI                 65536
SF           300.1599984 MHz
WDW                   EM
SSB      0
LB                  0.30 Hz
GB       0
PC                  1.00
```

N^2,6-bis(4-methoxyphenyl)-1,3,5-triazine-2,4-diamine (71)

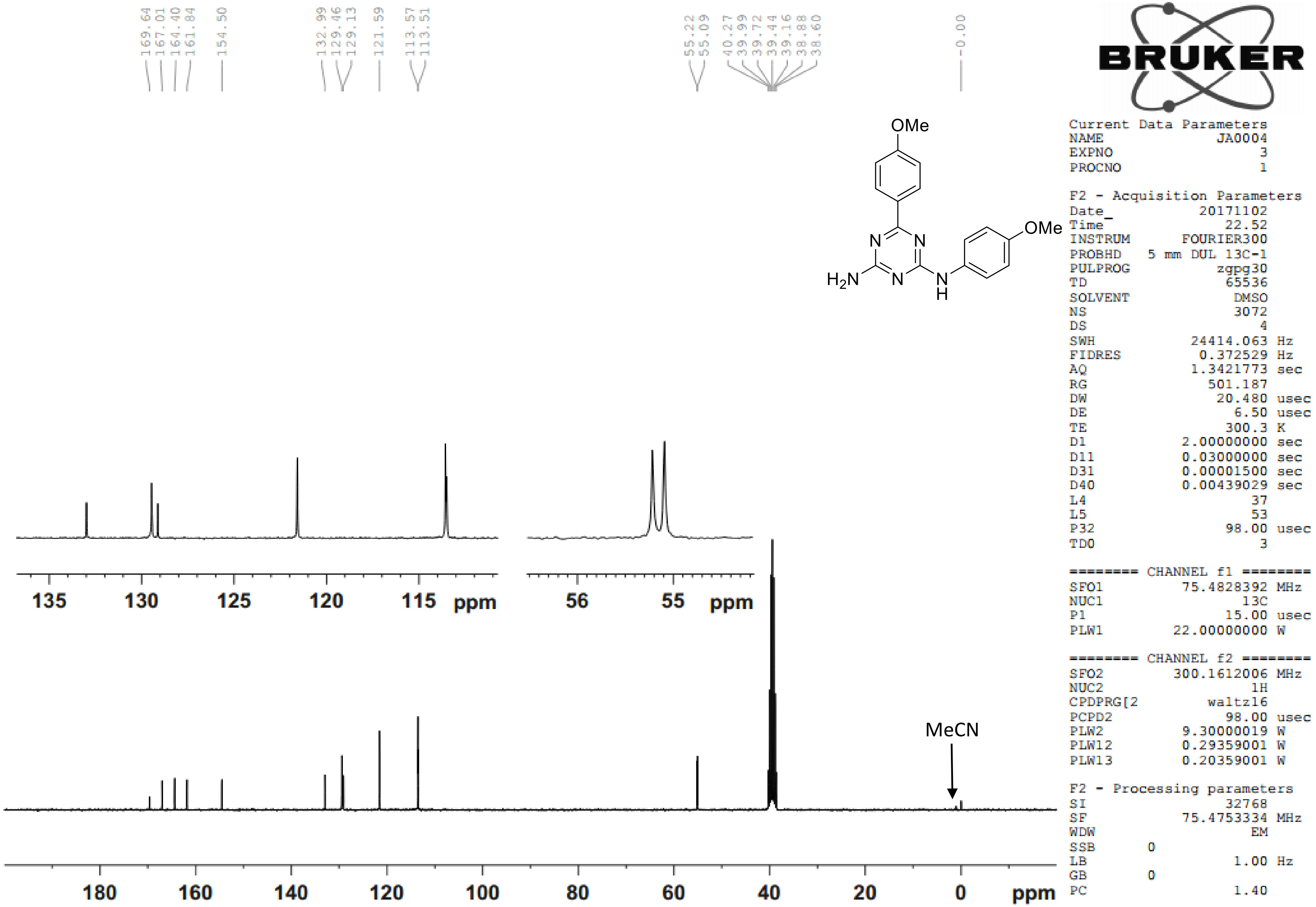

N[2]-(4-fluorophenyl)-6-(4-(trifluoromethyl)phenyl)-1,3,5-triazine-2,4-diamine (84)*

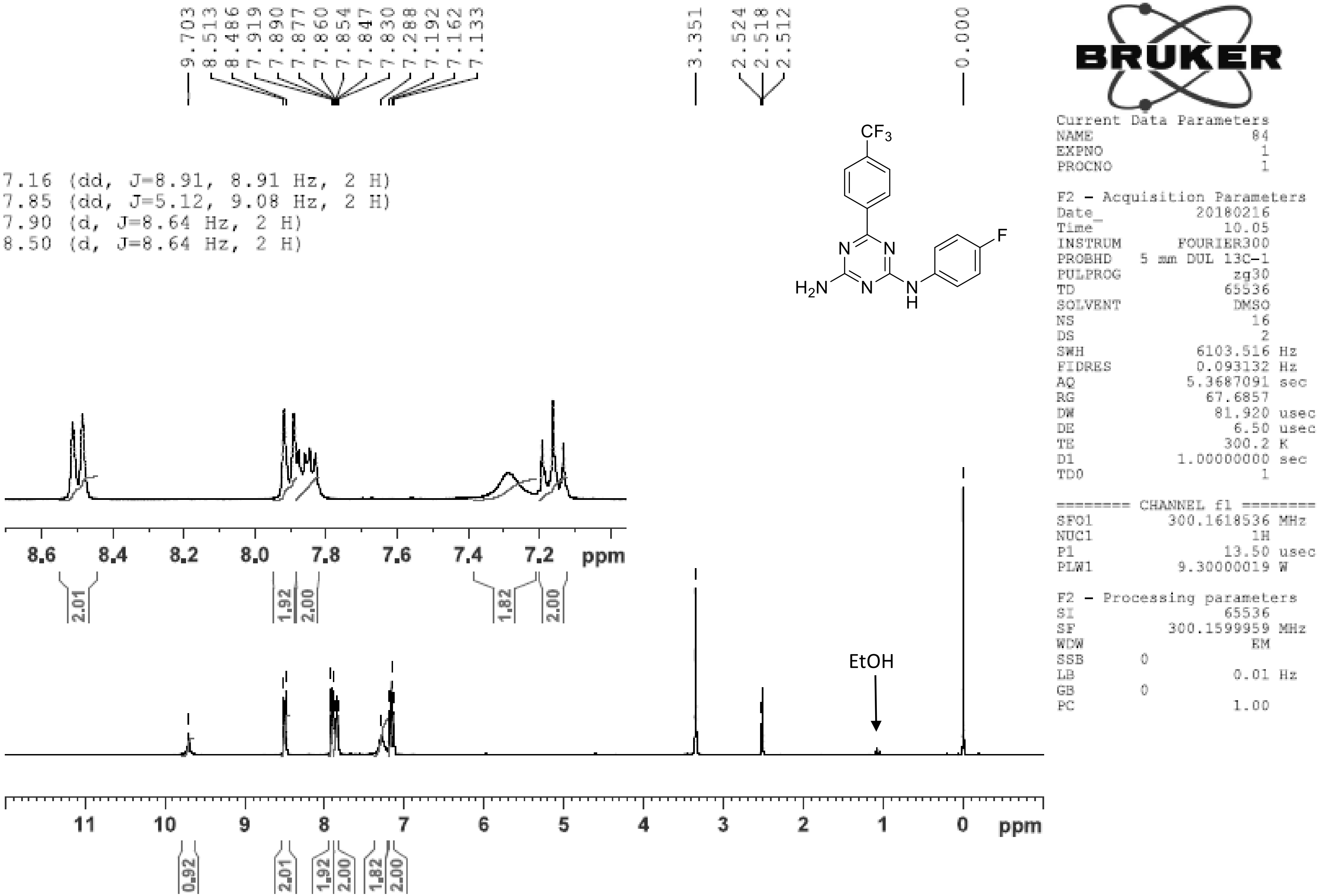

N*[2]-(4-fluorophenyl)-6-(4-(trifluoromethyl)phenyl)-1,3,5-triazine-2,4-diamine (84)

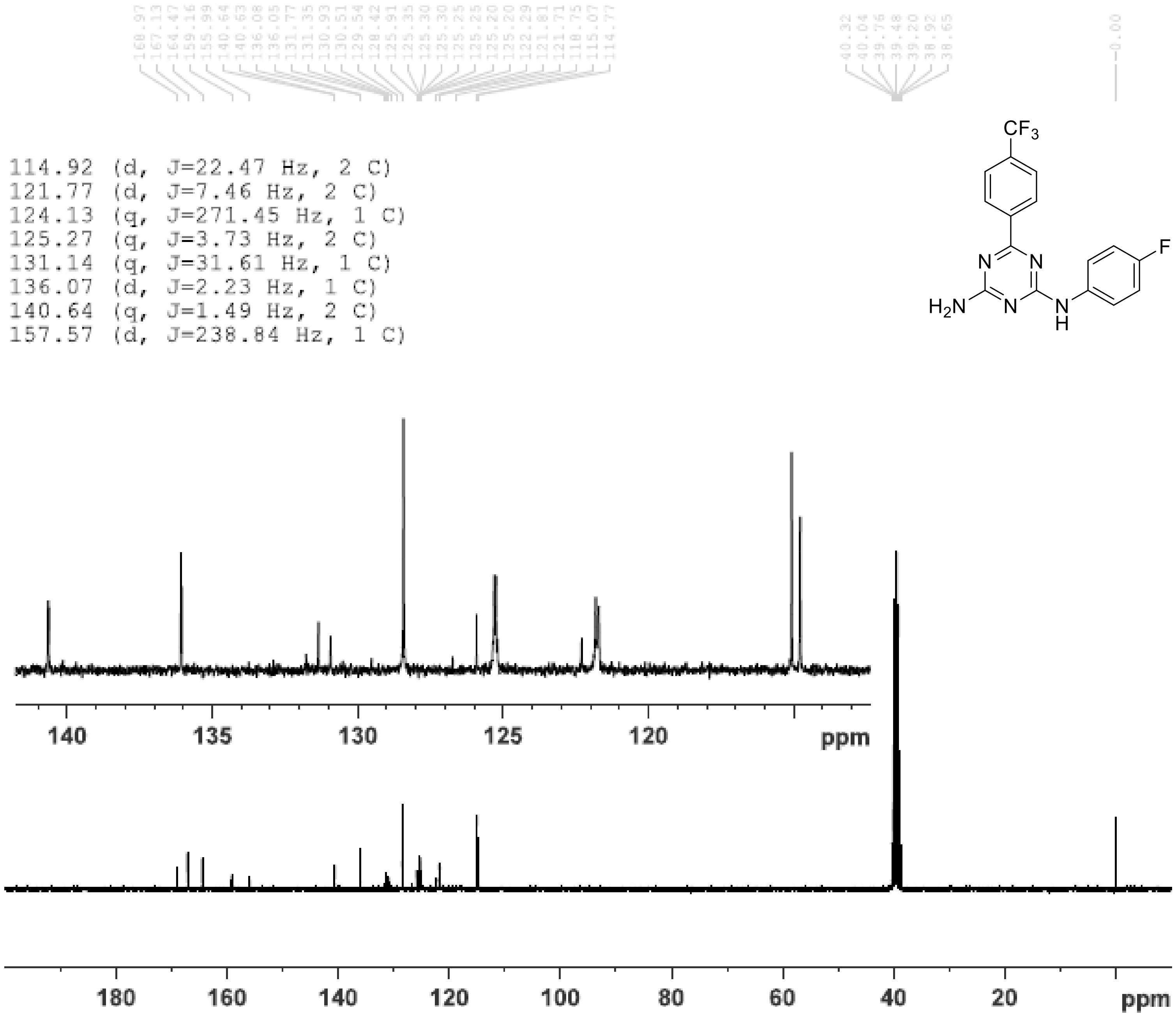

BRUKER

Current Data Parameters
NAME 84
EXPNO 2
PROCNO 1

F2 - Acquisition Parameters
Date_ 20180216
Time 10.11
INSTRUM FOURIER300
PROBHD 5 mm DUL 13C-1
PULPROG zgpg30
TD 65536
SOLVENT DMSO
NS 3072
DS 4
SWH 24414.063 Hz
FIDRES 0.372529 Hz
AQ 1.3421773 sec
RG 501.187
DW 20.480 usec
DE 6.50 usec
TE 300.3 K
D1 2.00000000 sec
D11 0.03000000 sec
D31 0.00001500 sec
D40 0.00439029 sec
L4 37
L5 53
P32 98.00 usec
TD0 3

======== CHANNEL f1 ========
SFO1 75.4828392 MHz
NUC1 13C
P1 15.00 usec
PLW1 22.00000000 W

======== CHANNEL f2 ========
SFO2 300.1612006 MHz
NUC2 1H
CPDPRG[2 waltz16
PCPD2 98.00 usec
PLW2 9.30000019 W
PLW12 0.29359001 W
PLW13 0.20359001 W

F2 - Processing parameters
SI 32768
SF 75.4753305 MHz
WDW EM
SSB 0
LB 0 Hz
GB 0
PC 1.40

N[2]-(4-fluorophenyl)-6-(4-(trifluoromethoxy)phenyl)-1,3,5-triazine-2,4-diamine (91)

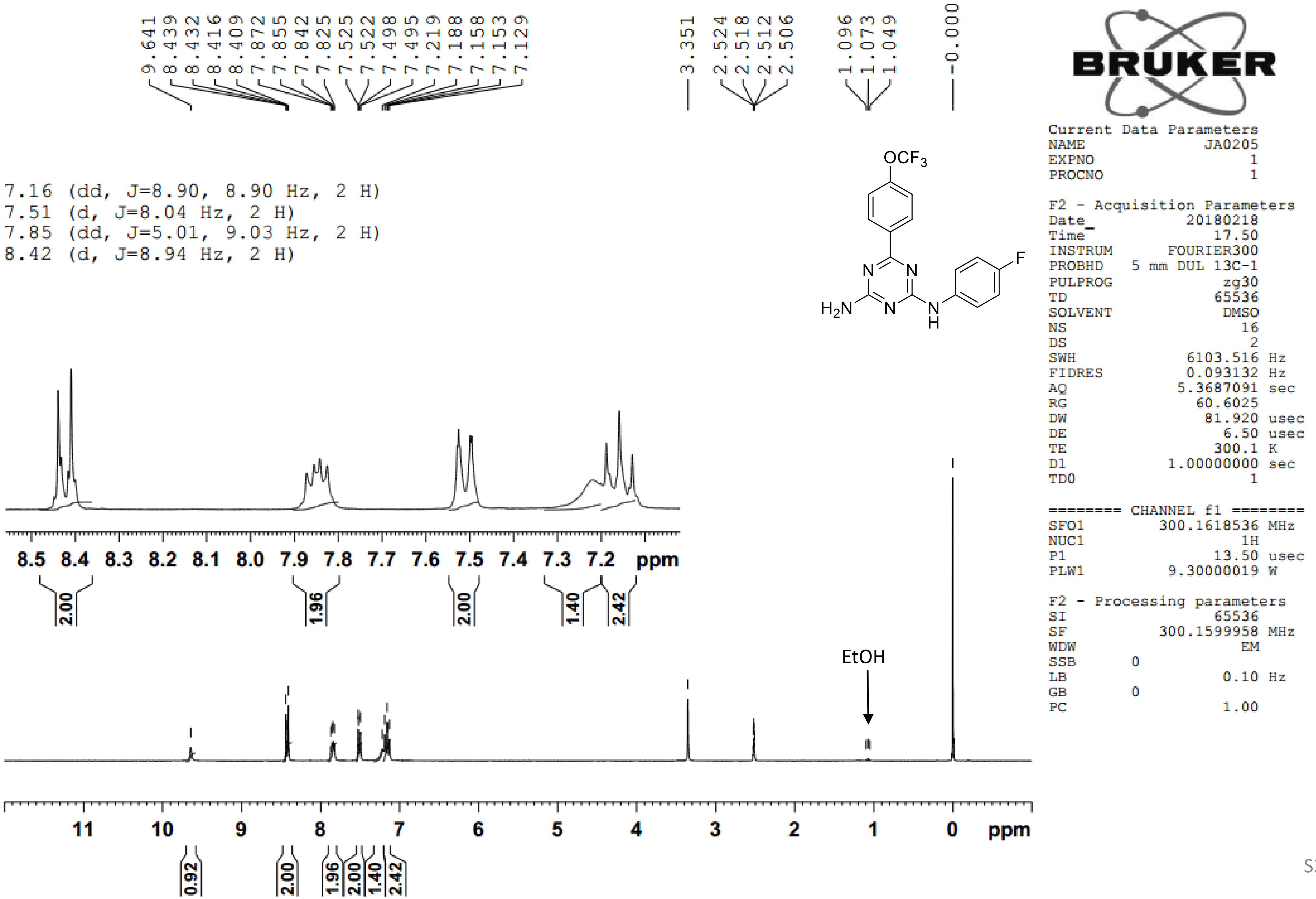

N²-(4-fluorophenyl)-6-(4-(trifluoromethoxy)phenyl)-1,3,5-triazine-2,4-diamine (91)

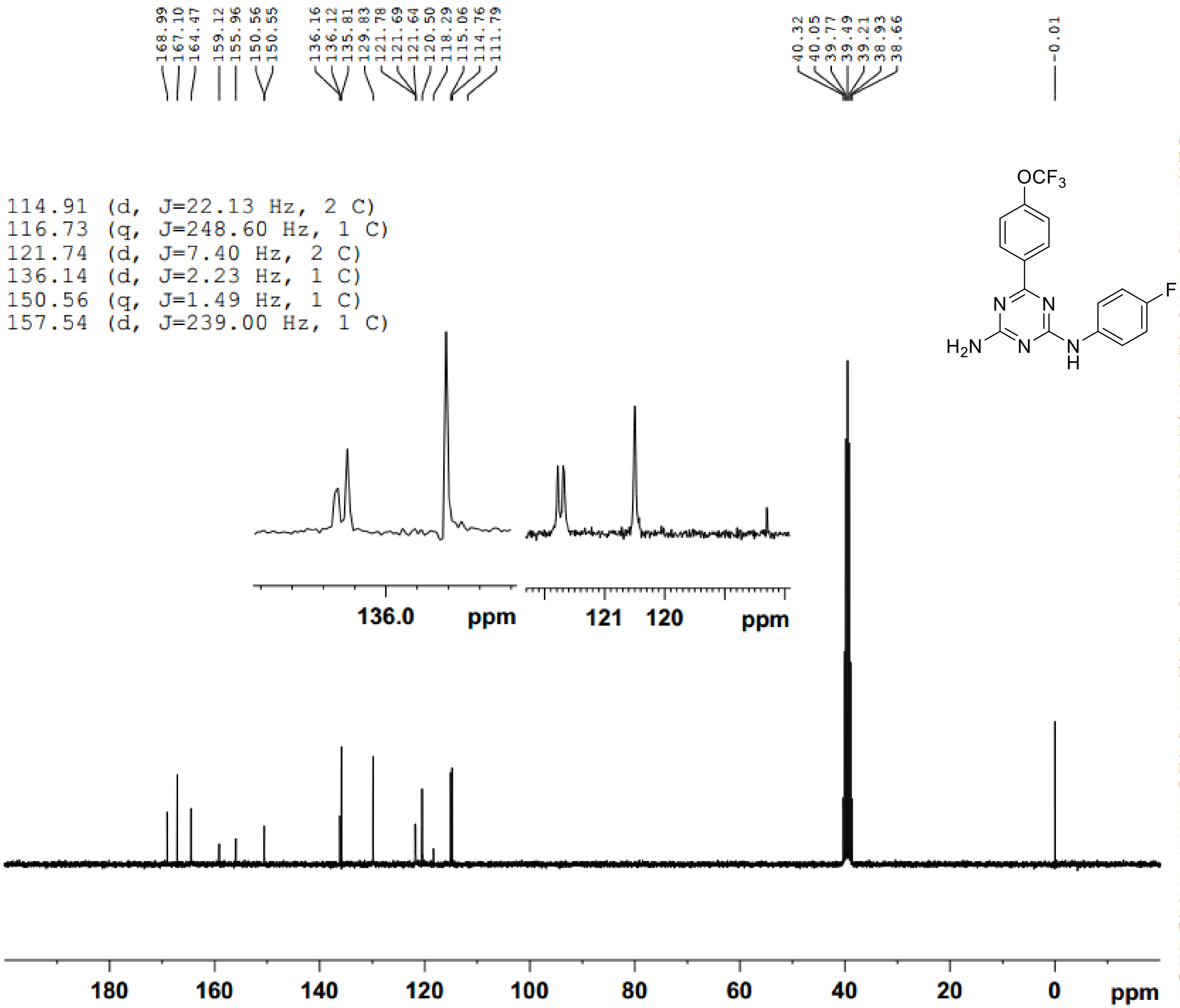

BRUKER

```
Current Data Parameters
NAME                 JA0205
EXPNO                     2
PROCNO                    1

F2 - Acquisition Parameters
Date_              20180219
Time                   2.54
INSTRUM          FOURIER300
PROBHD   5 mm DUL 13C-1
PULPROG              zgpg30
TD                    65536
SOLVENT                DMSO
NS                     3072
DS                        4
SWH               24414.063 Hz
FIDRES             0.372529 Hz
AQ                1.3421773 sec
RG                  501.187
DW                   20.480 usec
DE                     6.50 usec
TE                    300.3 K
D1               2.00000000 sec
D11              0.03000000 sec
D31              0.00001500 sec
D40              0.00439029 sec
L4                       37
L5                       53
P32                   98.00 usec
TD0                       3

======== CHANNEL f1 ========
SFO1            75.4828392 MHz
NUC1                   13C
P1                   15.00 usec
PLW1           22.00000000 W

======== CHANNEL f2 ========
SFO2           300.1612006 MHz
NUC2                    1H
CPDPRG[2           waltz16
PCPD2                98.00 usec
PLW2            9.30000019 W
PLW12           0.29359001 W
PLW13           0.20359001 W

F2 - Processing parameters
SI                   32768
SF              75.4753298 MHz
WDW                     EM
SSB      0
LB       0 Hz
GB       0
PC                    1.40
```

6-(4-(benzyloxy)phenyl)-N²-(4-fluorophenyl)-1,3,5-triazine-2,4-diamine (111)

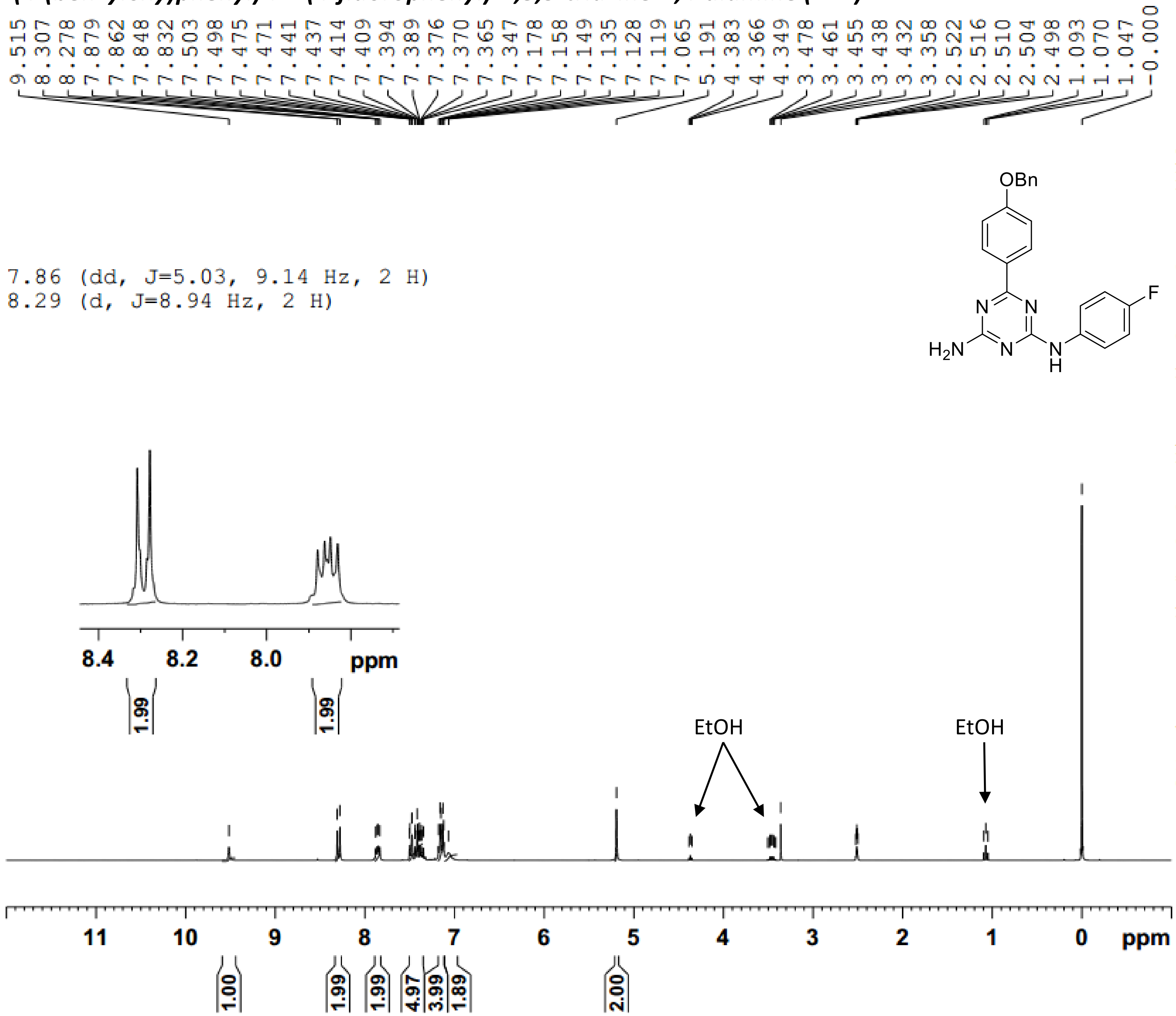

BRUKER

Current Data Parameters

NAME	JA0253
EXPNO	5
PROCNO	1

F2 - Acquisition Parameters

Date_	20190410
Time	14.51
INSTRUM	FOURIER300
PROBHD	5 mm DUL 13C-1
PULPROG	zg30
TD	65536
SOLVENT	DMSO
NS	16
DS	2
SWH	6103.516 Hz
FIDRES	0.093132 Hz
AQ	5.3687091 sec
RG	31.623
DW	81.920 usec
DE	6.50 usec
TE	298.2 K
D1	1.00000000 sec
TD0	1

======== CHANNEL f1 ========

SFO1	300.1618536 MHz
NUC1	1H
P1	13.50 usec
PLW1	9.30000019 W

F2 - Processing parameters

SI	65536
SF	300.1599978 MHz
WDW	EM
SSB	0
LB	0.30 Hz
GB	0
PC	1.00

6-(4-(benzyloxy)phenyl)-N²-(4-fluorophenyl)-1,3,5-triazine-2,4-diamine (111)

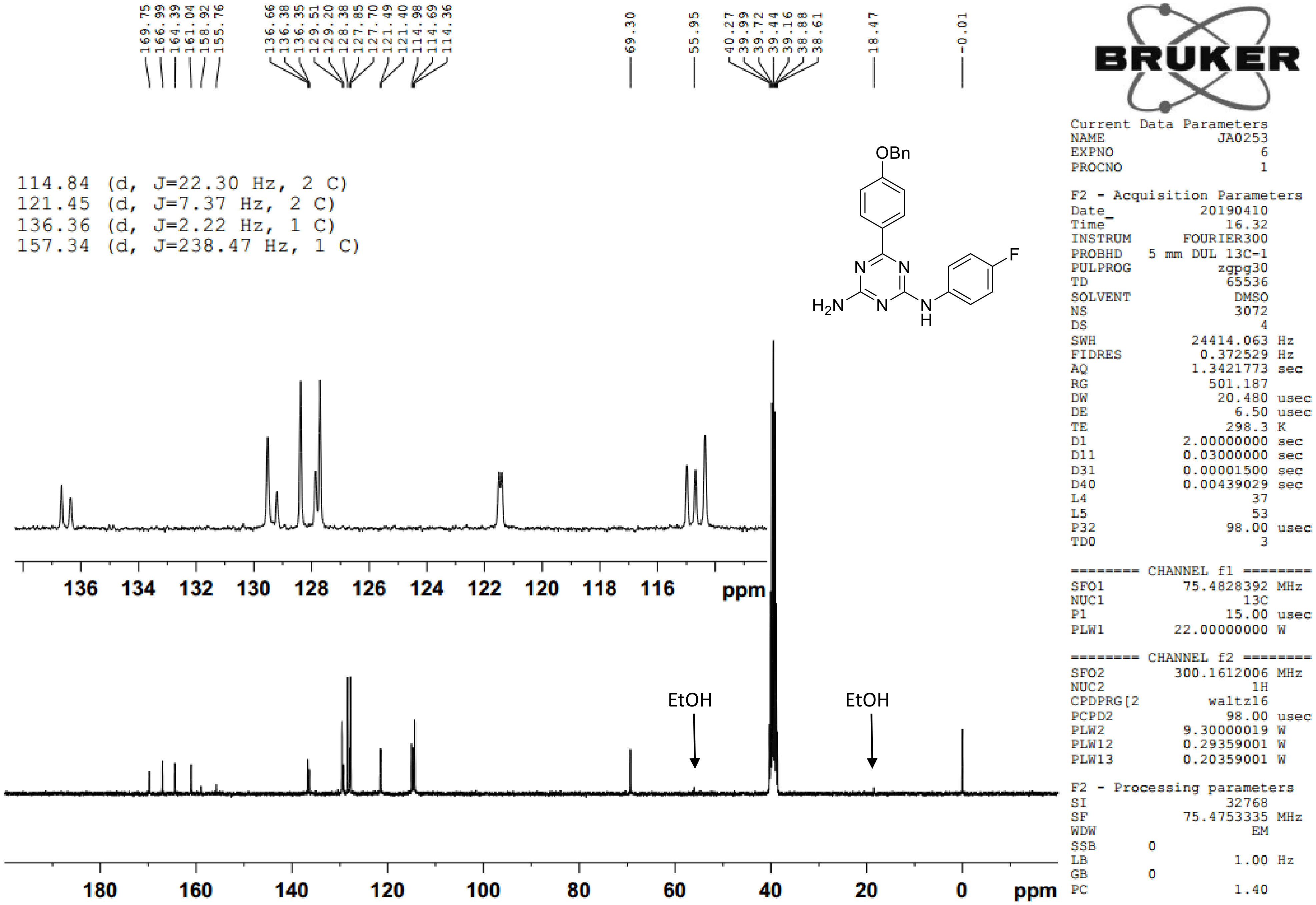

N[2]-(2-fluorophenyl)-6-(thiophen-2-yl)-1,3,5-triazine-2,4-diamine (116)

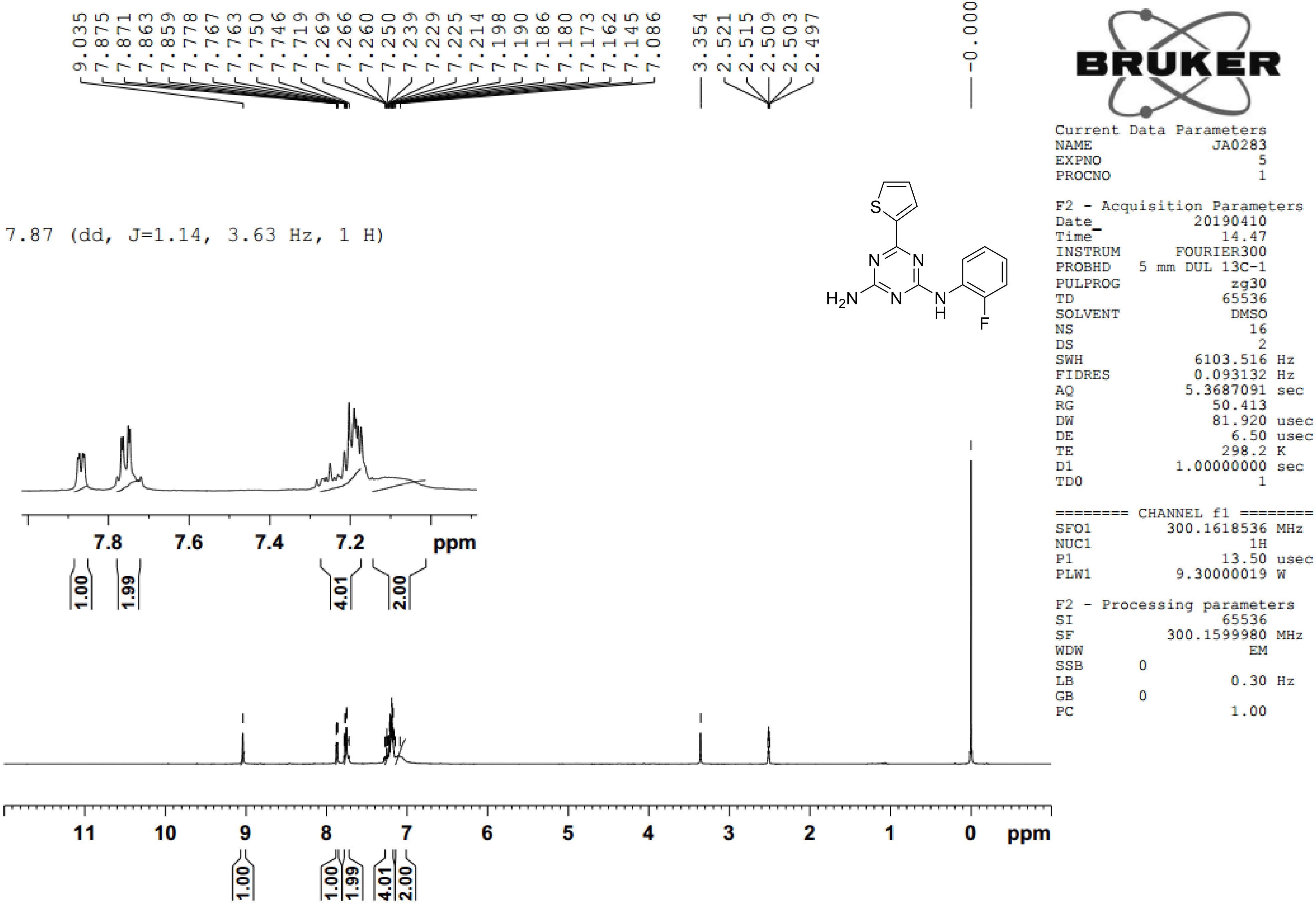

N^2-(2-fluorophenyl)-6-(thiophen-2-yl)-1,3,5-triazine-2,4-diamine (116)

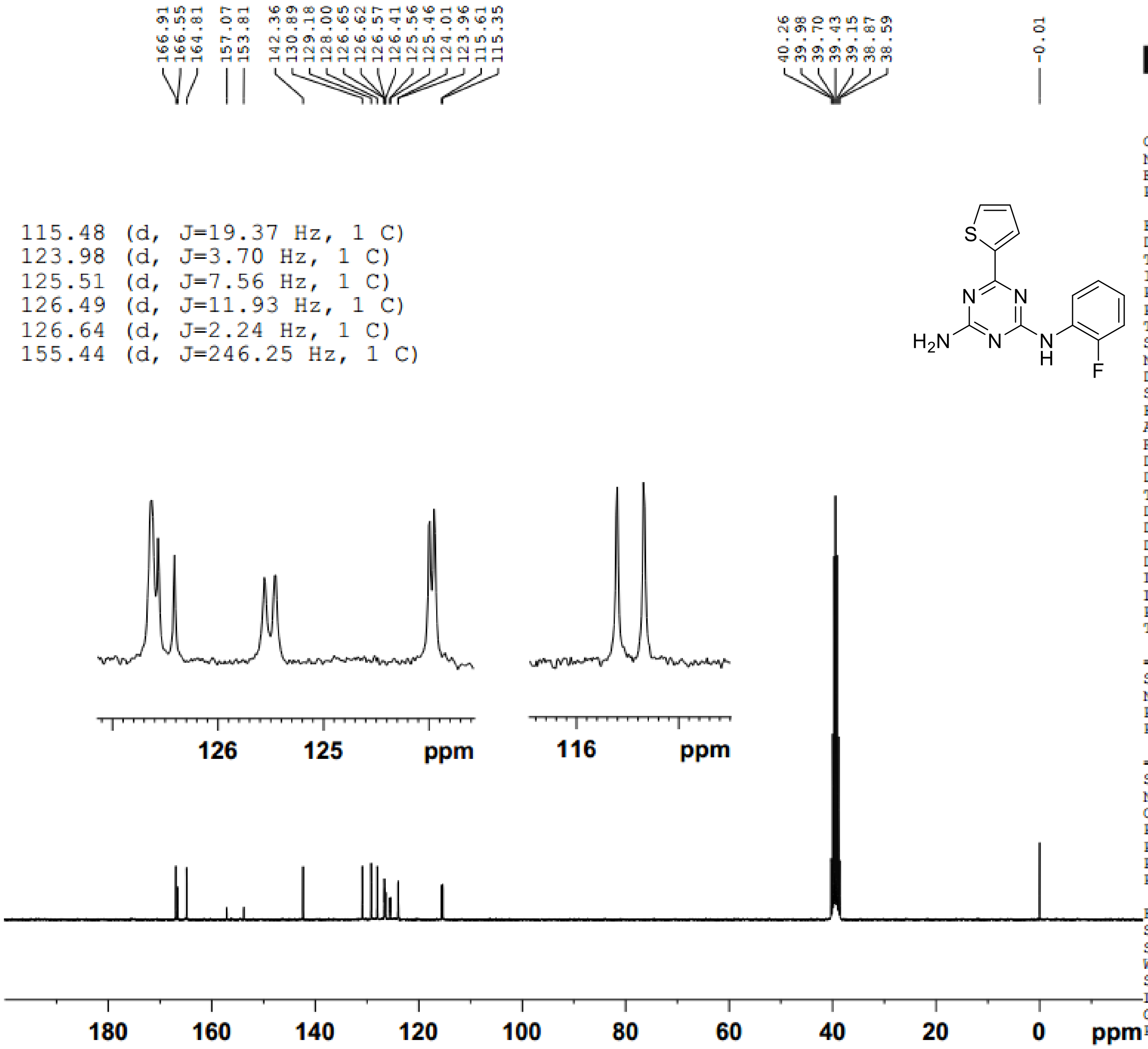


```
Current Data Parameters
NAME              JA0283
EXPNO                  6
PROCNO                 1

F2 - Acquisition Parameters
Date_           20190410
Time               22.28
INSTRUM       FOURIER300
PROBHD   5 mm DUL 13C-1
PULPROG          zgpg30
TD                65536
SOLVENT             DMSO
NS                  3072
DS                     4
SWH           24414.063 Hz
FIDRES         0.372529 Hz
AQ            1.3421773 sec
RG              501.187
DW               20.480 usec
DE                 6.50 usec
TE                298.3 K
D1           2.00000000 sec
D11          0.03000000 sec
D31          0.00001500 sec
D40          0.00439029 sec
L4                    37
L5                    53
P32                98.00 usec
TD0                    3

======== CHANNEL f1 ========
SFO1         75.4828392 MHz
NUC1                 13C
P1                 15.00 usec
PLW1         22.00000000 W

======== CHANNEL f2 ========
SFO2        300.1612006 MHz
NUC2                  1H
CPDPRG[2         waltz16
PCPD2              98.00 usec
PLW2          9.30000019 W
PLW12         0.29359001 W
PLW13         0.20359001 W

F2 - Processing parameters
SI                 32768
SF            75.4753342 MHz
WDW                   EM
SSB       0
LB                  1.00 Hz
GB        0
PC                  1.40
```

N[2]-(2-methoxyphenyl)-6-(thiophen-2-yl)-1,3,5-triazine-2,4-diamine (122)

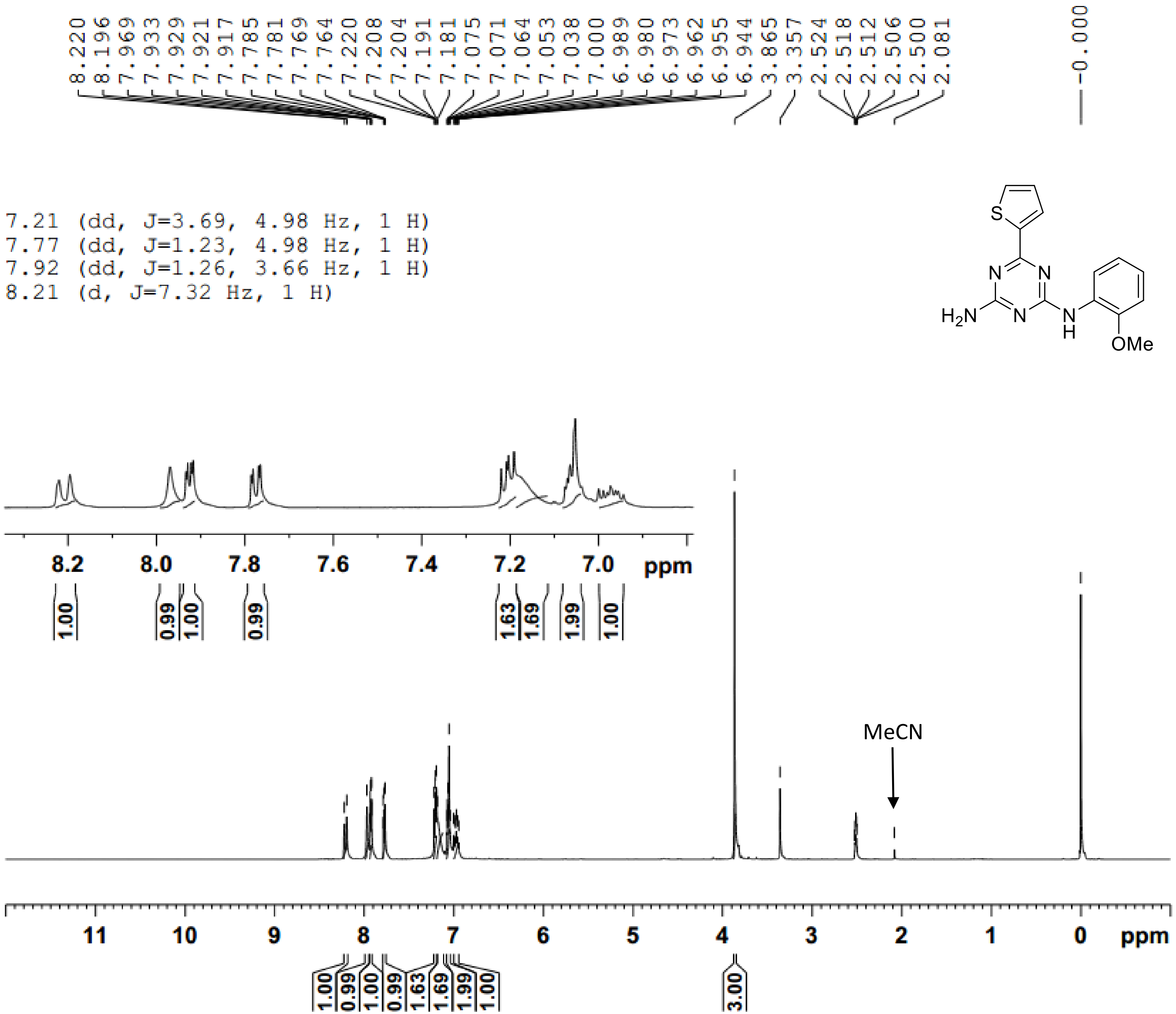


```
Current Data Parameters
NAME                JA0282
EXPNO                    2
PROCNO                   1

F2 - Acquisition Parameters
Date_             20181227
Time                 15.53
INSTRUM         FOURIER300
PROBHD    5 mm DUL 13C-1
PULPROG               zg30
TD                   65536
SOLVENT               DMSO
NS                      16
DS                       2
SWH              6103.516 Hz
FIDRES           0.093132 Hz
AQ              5.3687091 sec
RG                  31.623
DW                  81.920 usec
DE                    6.50 usec
TE                   300.2 K
D1              1.00000000 sec
TD0                      1

======== CHANNEL f1 ========
SFO1        300.1618536 MHz
NUC1                 1H
P1                13.50 usec
PLW1         9.30000019 W

F2 - Processing parameters
SI                   65536
SF          300.1599976 MHz
WDW                     EM
SSB      0
LB                    0.30 Hz
GB       0
PC                    1.00
```

N^2-(2-methoxyphenyl)-6-(thiophen-2-yl)-1,3,5-triazine-2,4-diamine (122)

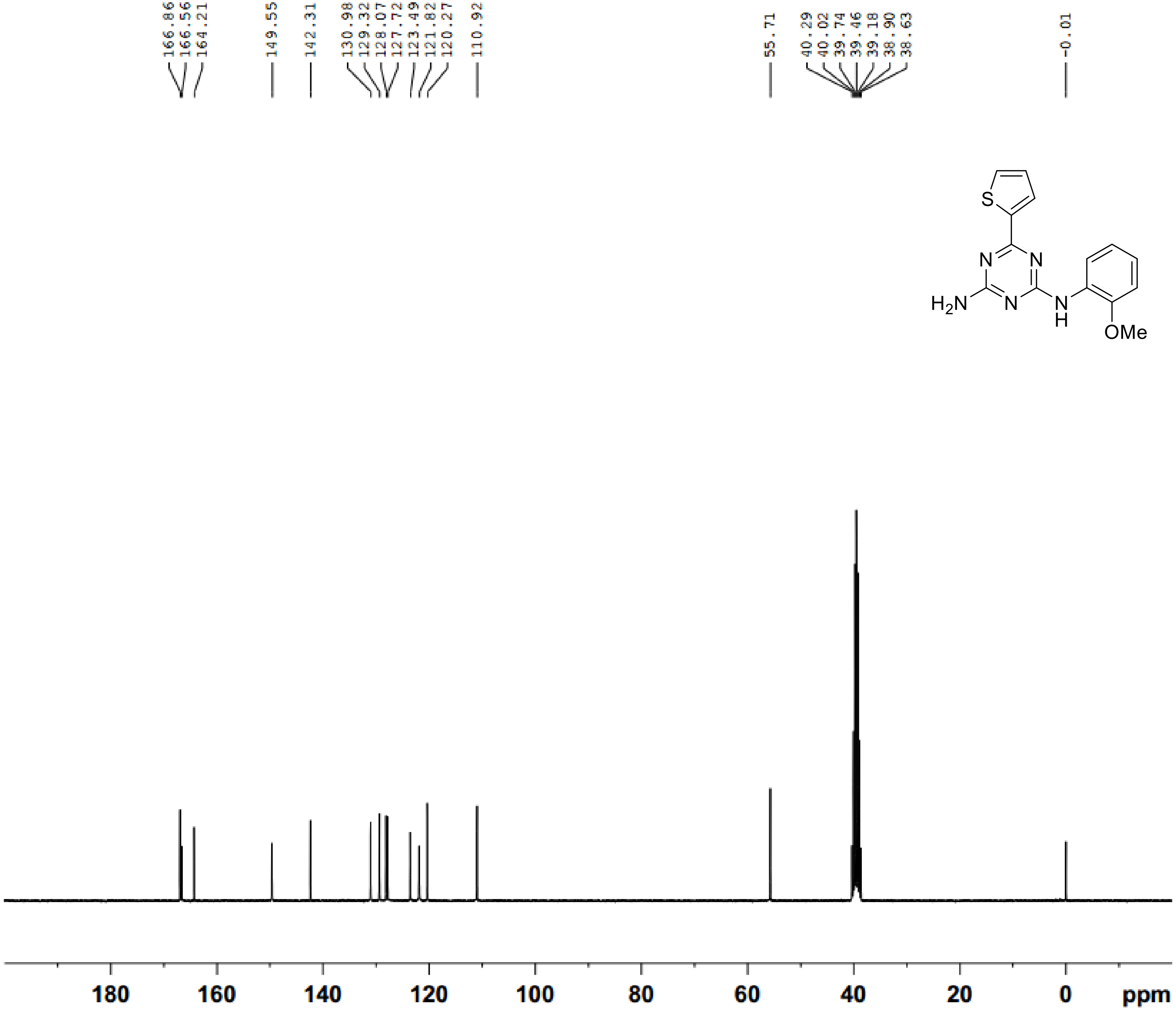

BRUKER

```
Current Data Parameters
NAME                JA0282
EXPNO                    3
PROCNO                   1

F2 - Acquisition Parameters
Date_             20181228
Time                 18.09
INSTRUM         FOURIER300
PROBHD   5 mm DUL 13C-1
PULPROG            zgpg30
TD                   65536
SOLVENT               DMSO
NS                    4096
DS                       4
SWH              24414.063 Hz
FIDRES            0.372529 Hz
AQ               1.3421773 sec
RG                 501.187
DW                  20.480 usec
DE                    6.50 usec
TE                   300.3 K
D1              2.00000000 sec
D11             0.03000000 sec
D31             0.00001500 sec
D40             0.00439029 sec
L4                      37
L5                      53
P32                  98.00 usec
TD0                      4

======== CHANNEL f1 ========
SFO1           75.4828392 MHz
NUC1                   13C
P1                   15.00 usec
PLW1          22.00000000 W

======== CHANNEL f2 ========
SFO2          300.1612006 MHz
NUC2                    1H
CPDPRG[2           waltz16
PCPD2                98.00 usec
PLW2            9.30000019 W
PLW12           0.29359001 W
PLW13           0.20359001 W

F2 - Processing parameters
SI                   32768
SF              75.4753327 MHz
WDW                     EM
SSB        0
LB                    1.00 Hz
GB         0
PC                    1.40
```

N^2-(4-methoxyphenyl)-6-(thiophen-2-yl)-1,3,5-triazine-2,4-diamine (123)

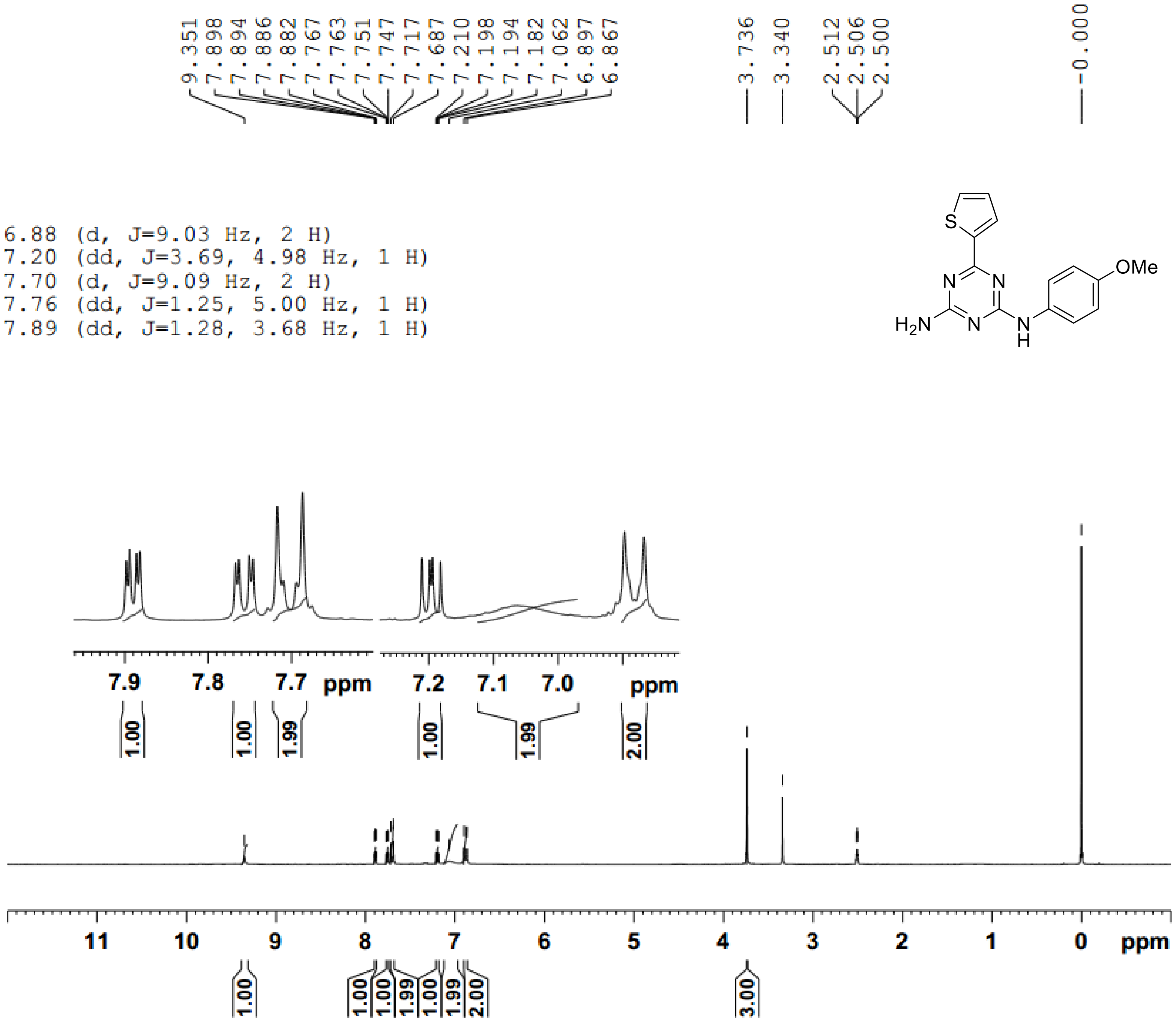

6.88 (d, J=9.03 Hz, 2 H)
7.20 (dd, J=3.69, 4.98 Hz, 1 H)
7.70 (d, J=9.09 Hz, 2 H)
7.76 (dd, J=1.25, 5.00 Hz, 1 H)
7.89 (dd, J=1.28, 3.68 Hz, 1 H)


```
Current Data Parameters
NAME              JA0276
EXPNO                  6
PROCNO                 1

F2 - Acquisition Parameters
Date_           20190410
Time               14.42
INSTRUM       FOURIER300
PROBHD   5 mm DUL 13C-1
PULPROG             zg30
TD                 65536
SOLVENT             DMSO
NS                    16
DS                     2
SWH             6103.516 Hz
FIDRES          0.093132 Hz
AQ             5.3687091 sec
RG               66.0182
DW                81.920 usec
DE                  6.50 usec
TE                 298.2 K
D1            1.00000000 sec
TD0                    1

======== CHANNEL f1 ========
SFO1         300.1618536 MHz
NUC1                  1H
P1                 13.50 usec
PLW1          9.30000019 W

F2 - Processing parameters
SI                 65536
SF           300.1599989 MHz
WDW                   EM
SSB      0
LB                  0.30 Hz
GB       0
PC                  1.00
```

N²-(4-methoxyphenyl)-6-(thiophen-2-yl)-1,3,5-triazine-2,4-diamine (123)

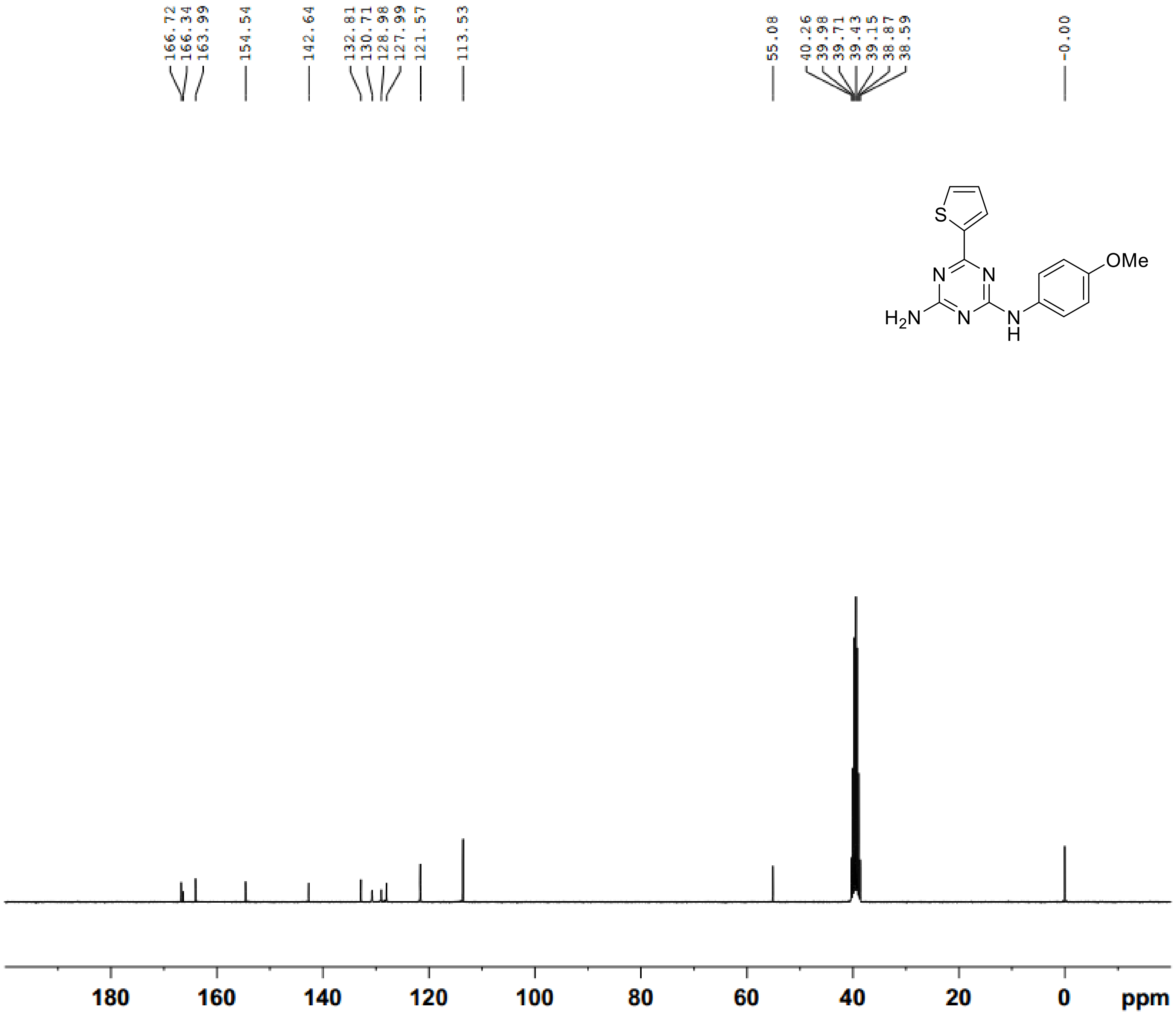


```
Current Data Parameters
NAME              JA0276
EXPNO                  7
PROCNO                 1

F2 - Acquisition Parameters
Date_           20190411
Time                1.26
INSTRUM       FOURIER300
PROBHD   5 mm DUL 13C-1
PULPROG          zgpg30
TD                 65536
SOLVENT             DMSO
NS                  5120
DS                     4
SWH            24414.063 Hz
FIDRES          0.372529 Hz
AQ             1.3421773 sec
RG               501.187
DW                20.480 usec
DE                  6.50 usec
TE                 298.3 K
D1            2.00000000 sec
D11           0.03000000 sec
D31           0.00001500 sec
D40           0.00439029 sec
L4                    37
L5                    53
P32                98.00 usec
TD0                    5

======== CHANNEL f1 ========
SFO1          75.4828392 MHz
NUC1                 13C
P1                 15.00 usec
PLW1         22.00000000 W

======== CHANNEL f2 ========
SFO2         300.1612006 MHz
NUC2                  1H
CPDPRG[2         waltz16
PCPD2              98.00 usec
PLW2          9.30000019 W
PLW12         0.29359001 W
PLW13         0.20359001 W

F2 - Processing parameters
SI                 32768
SF            75.4753342 MHz
WDW                   EM
SSB      0
LB                  1.00 Hz
GB       0
PC                  1.40
```

Concentration-response curves for the most active compounds
(**14**, **16-19**, **36**, **56**, **58**, **61**, **62**, **70**, **73**, **74**, **77**, **78**, **81**, **95**, **99**, **100-103**, **110**, **120**, and **121**)
tested against MDA-MB231 breast cancer cells

Compound 14

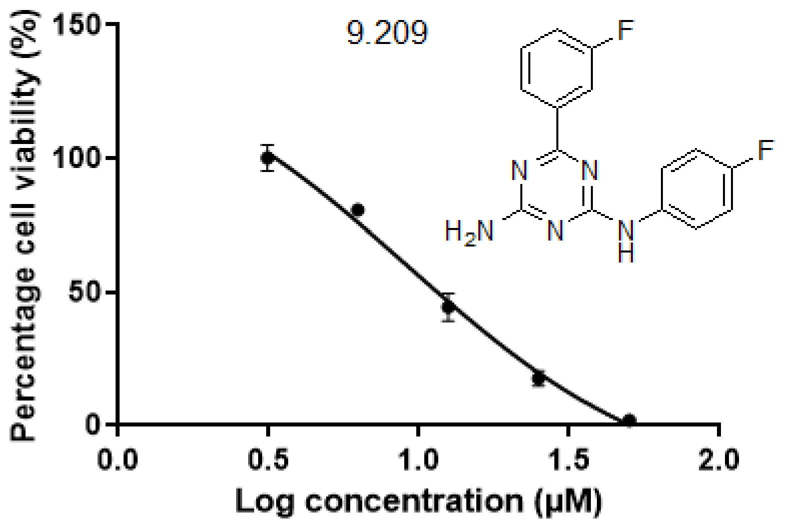

Compound 17

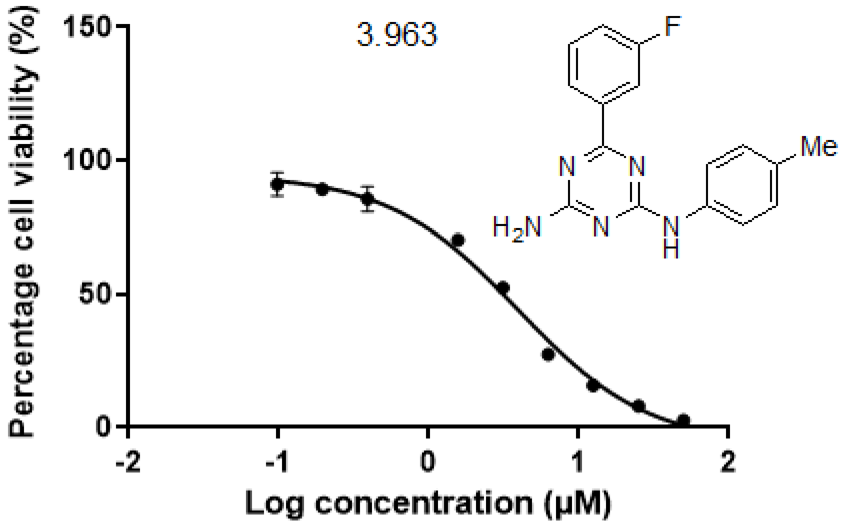

Compound 16

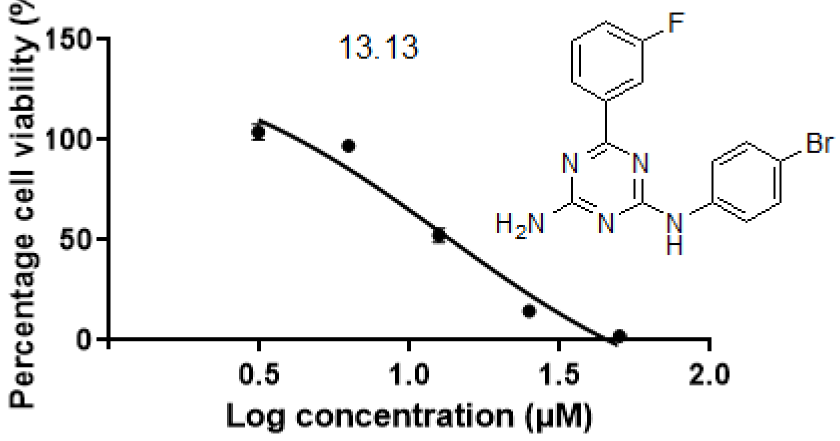

Compound 18

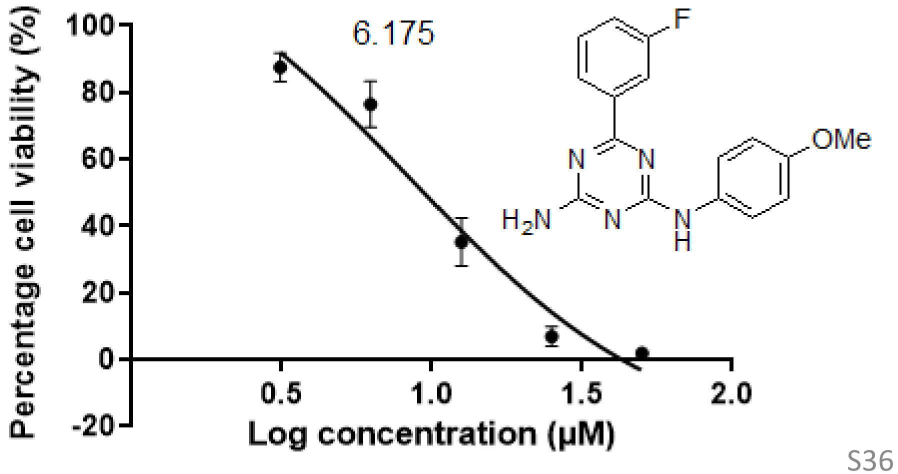

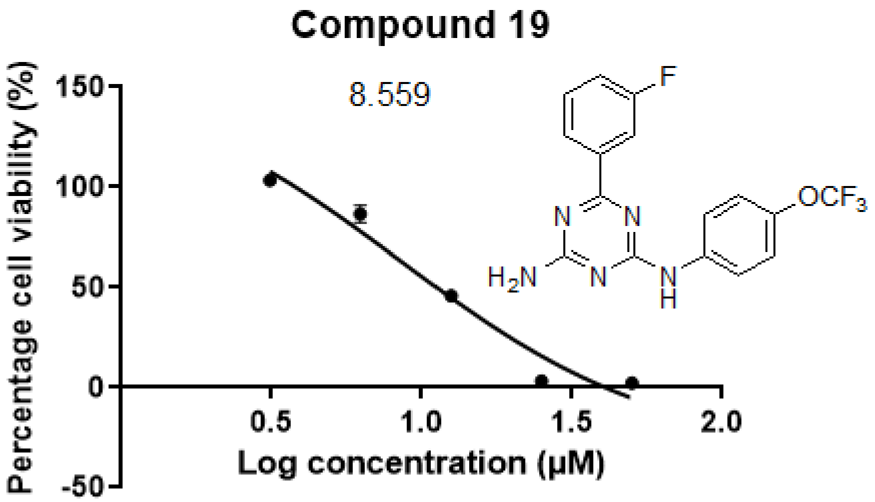
Compound 19
8.559
F
OCF3
N
N
H2N
N
N
H
Percentage cell viability (%)
Log concentration (μM)
150
100
50
0
-50
0.5
1.0
1.5
2.0

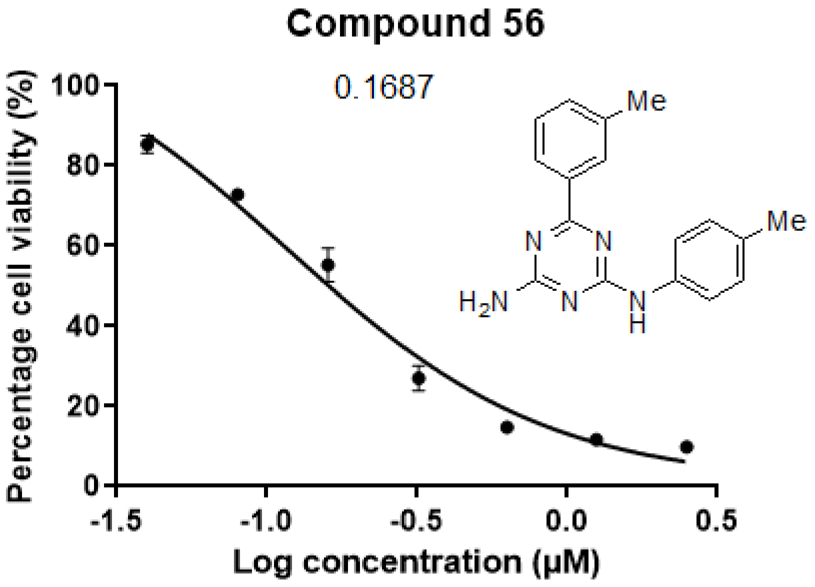
Compound 56
0.1687
Me
Me
N
N
H2N
N
N
H
Percentage cell viability (%)
Log concentration (μM)
100
80
60
40
20
0
-1.5
-1.0
-0.5
0.0
0.5

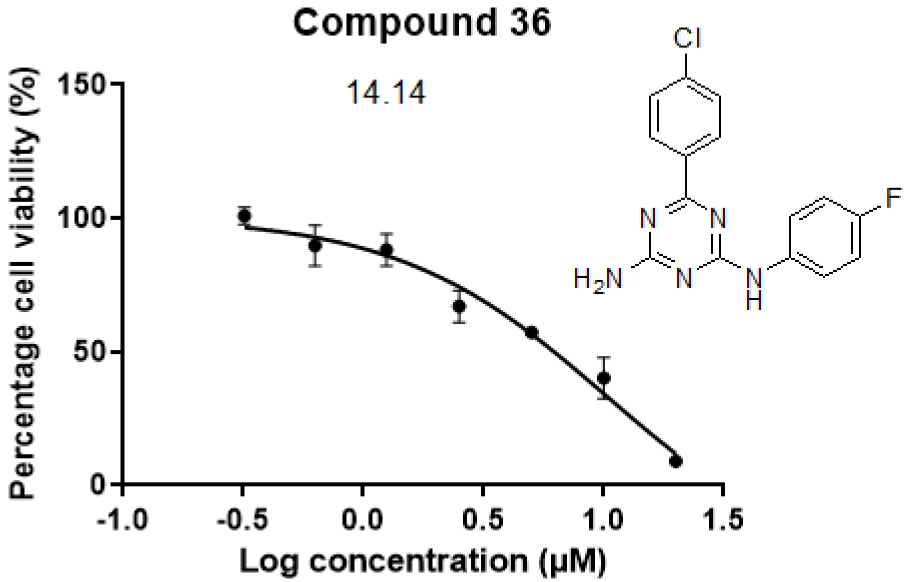
Compound 36
14.14
Cl
F
N
N
H2N
N
N
H
Percentage cell viability (%)
Log concentration (μM)
150
100
50
0
-1.0
-0.5
0.0
0.5
1.0
1.5

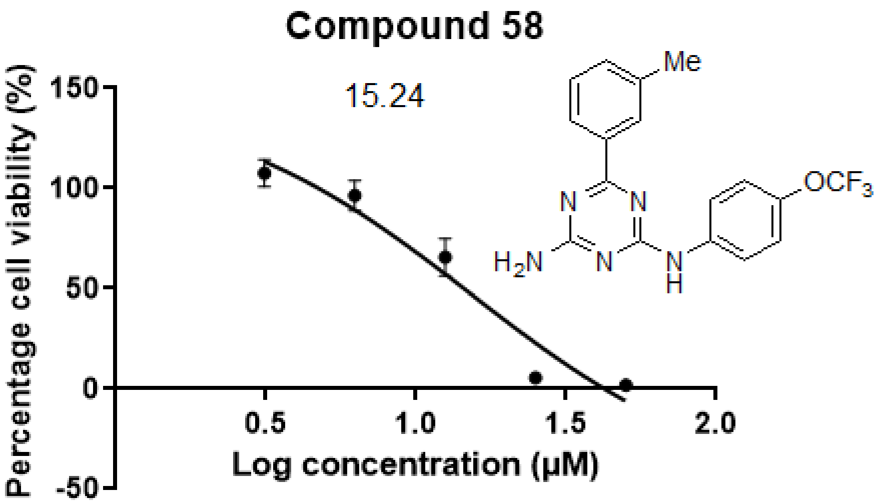
Compound 58
15.24
Me
OCF3
N
N
H2N
N
N
H
Percentage cell viability (%)
Log concentration (μM)
150
100
50
0
-50
0.5
1.0
1.5
2.0

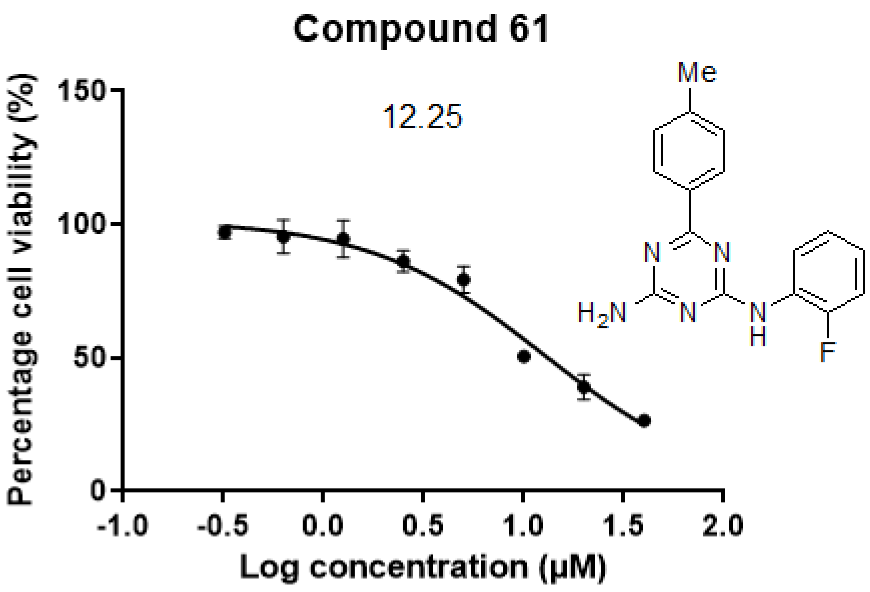
Compound 61
12.25
Me
N
N
H2N
N
N
H
F
Percentage cell viability (%)
150
100
50
0
-1.0
-0.5
0.0
0.5
1.0
1.5
2.0
Log concentration (μM)

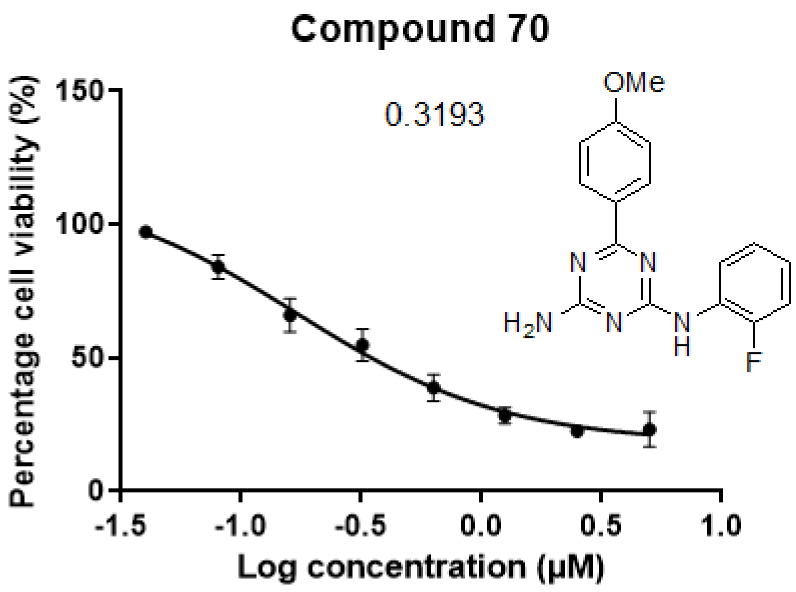
Compound 70
0.3193
OMe
N
N
H2N
N
N
H
F
Percentage cell viability (%)
150
100
50
0
-1.5
-1.0
-0.5
0.0
0.5
1.0
Log concentration (μM)

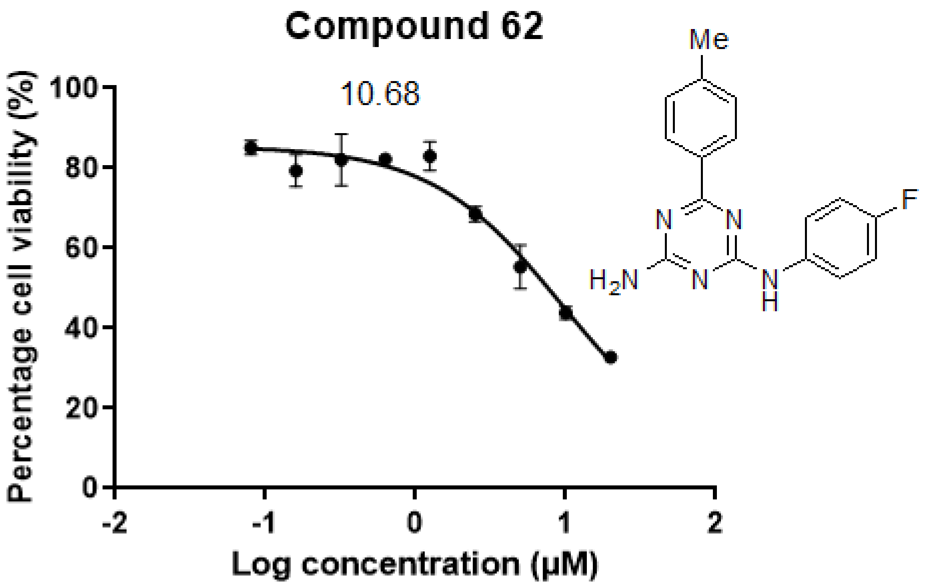
Compound 62
10.68
Me
N
N
H2N
N
N
H
F
Percentage cell viability (%)
100
80
60
40
20
0
-2
-1
0
1
2
Log concentration (μM)

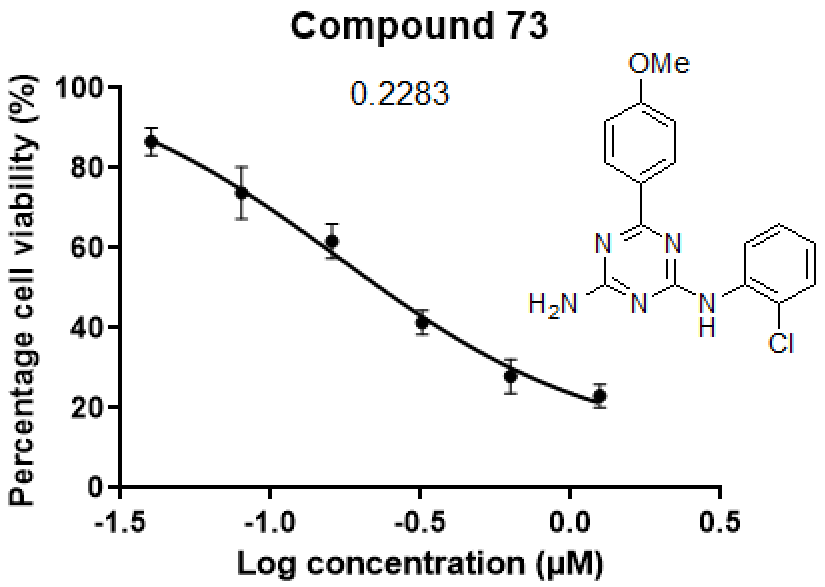
Compound 73
0.2283
OMe
N
N
H2N
N
N
H
Cl
Percentage cell viability (%)
100
80
60
40
20
0
-1.5
-1.0
-0.5
0.0
0.5
Log concentration (μM)

Compound 74

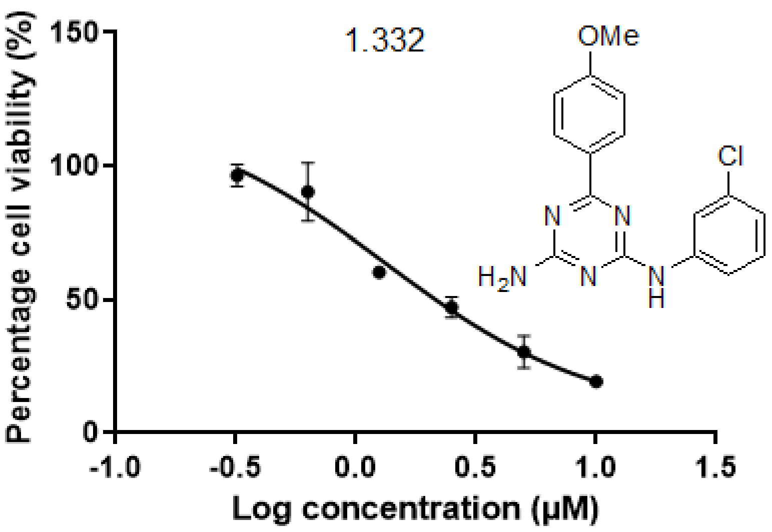

Compound 78

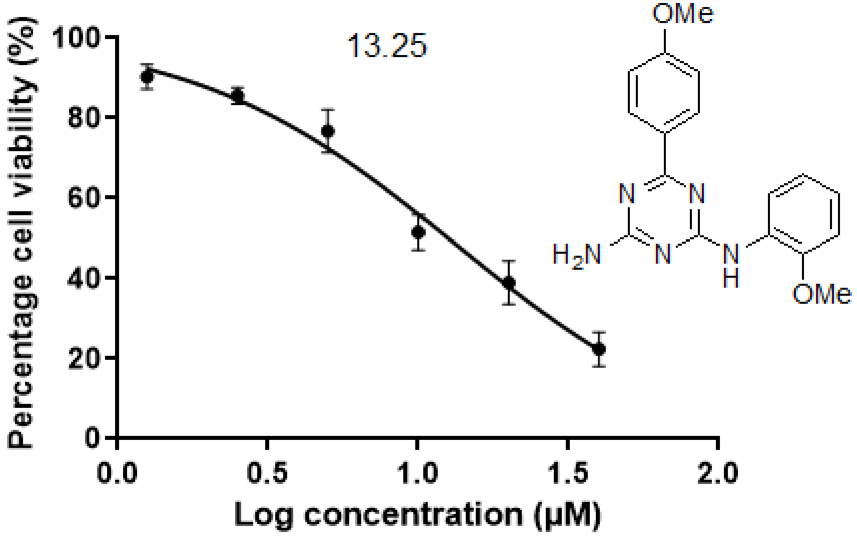

Compound 77

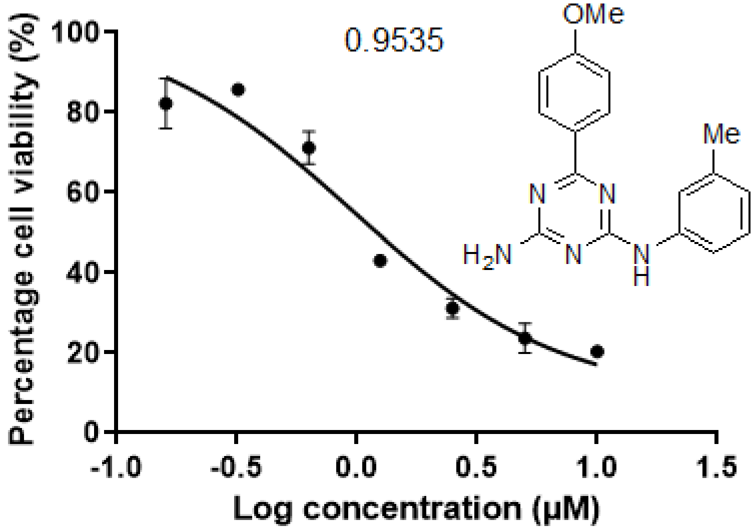

Compound 81

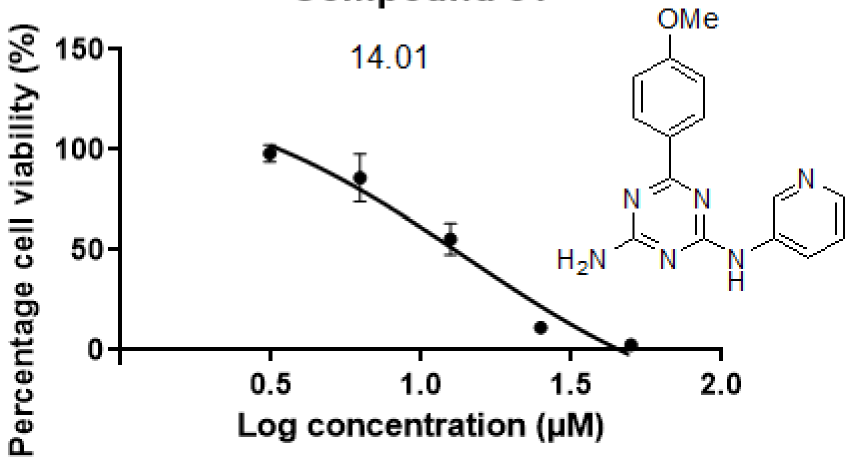

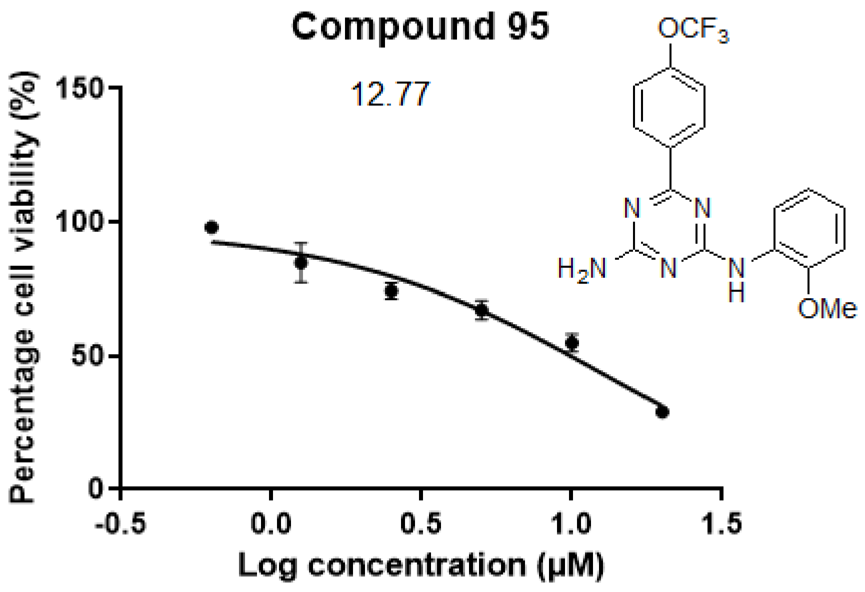
Compound 95
12.77
OCF3
N
N
H2N
N
N
H
OMe
Percentage cell viability (%)
150
100
50
0
-0.5
0.0
0.5
1.0
1.5
Log concentration (μM)

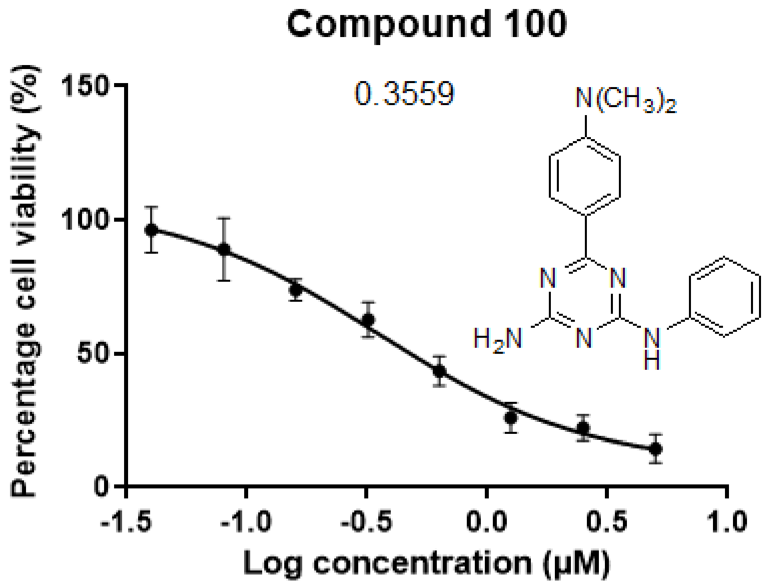
Compound 100
0.3559
N(CH3)2
N
N
H2N
N
N
H
Percentage cell viability (%)
150
100
50
0
-1.5
-1.0
-0.5
0.0
0.5
1.0
Log concentration (μM)

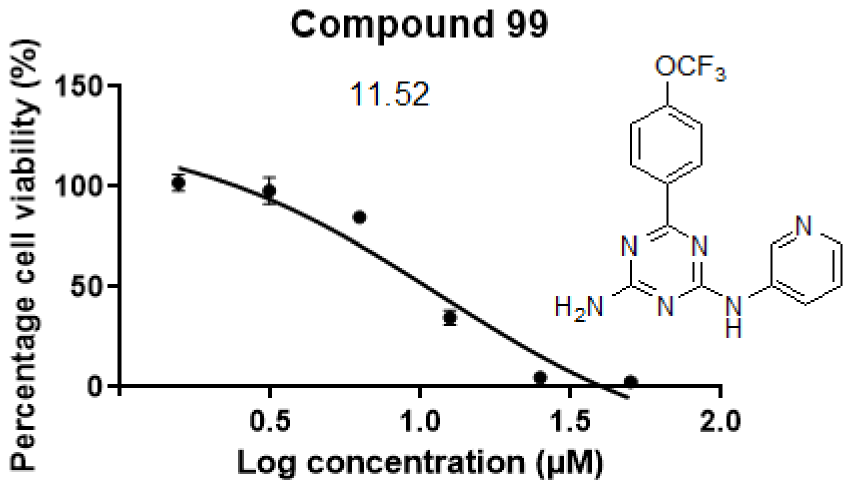
Compound 99
11.52
OCF3
N
N
N
H2N
N
N
H
Percentage cell viability (%)
150
100
50
0
0.5
1.0
1.5
2.0
Log concentration (μM)

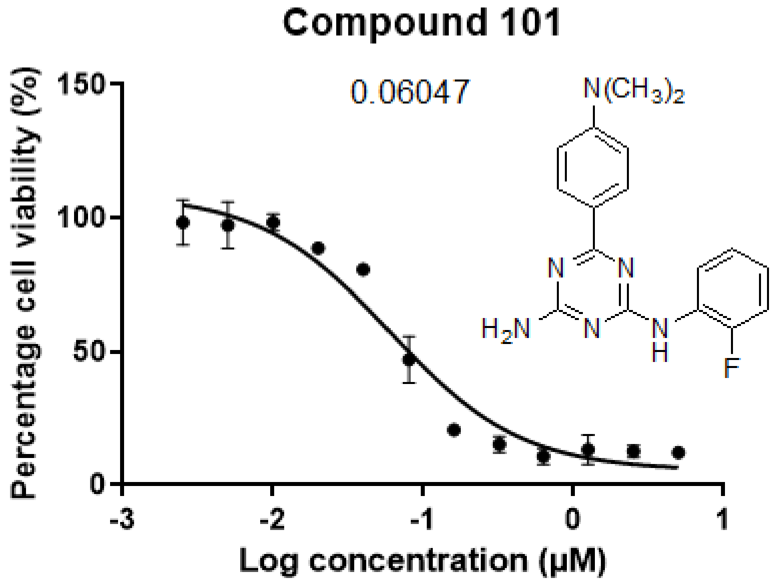
Compound 101
0.06047
N(CH3)2
N
N
H2N
N
N
H
F
Percentage cell viability (%)
150
100
50
0
-3
-2
-1
0
1
Log concentration (μM)

Compound 102

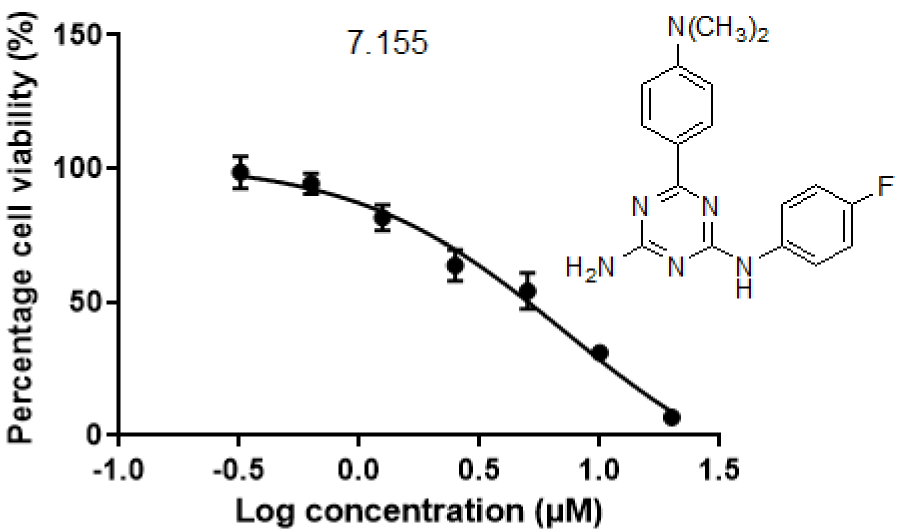

Compound 110

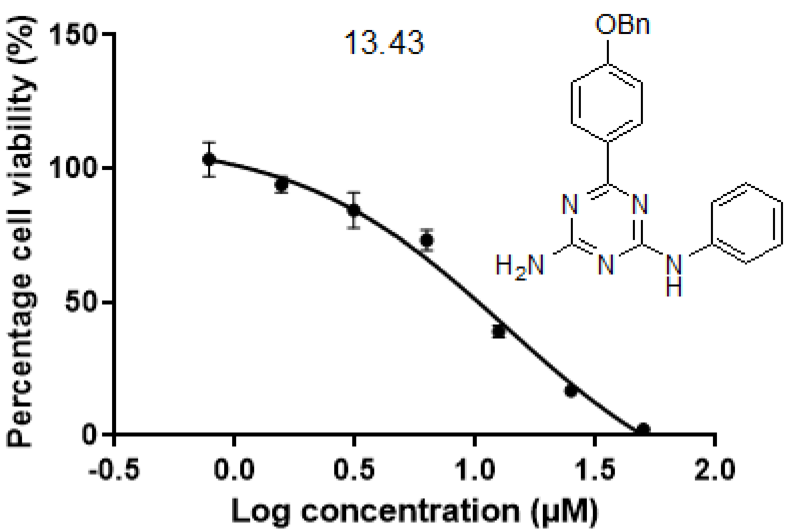

Compound 103

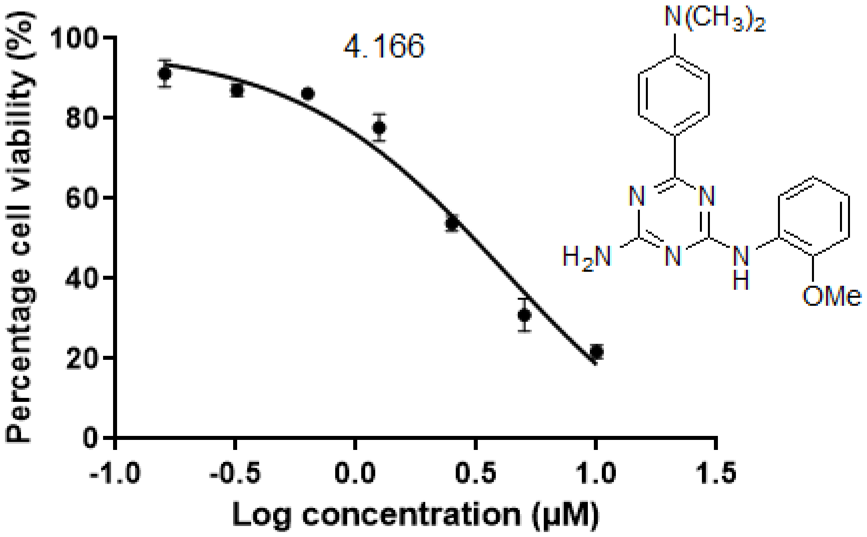

Compound 120

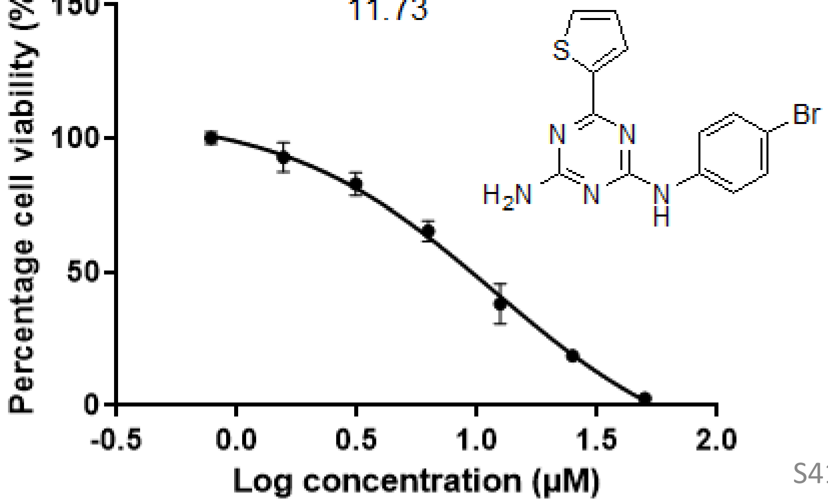

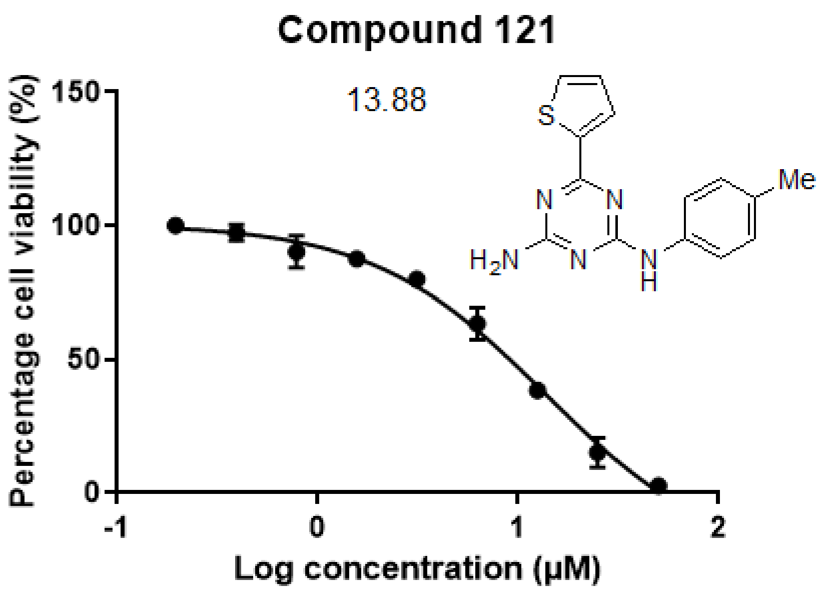
Compound 121
13.88
S
N
N
Me
H2N
N
N
H
Percentage cell viability (%)
150
100
50
0
-1
0
1
2
Log concentration (μM)

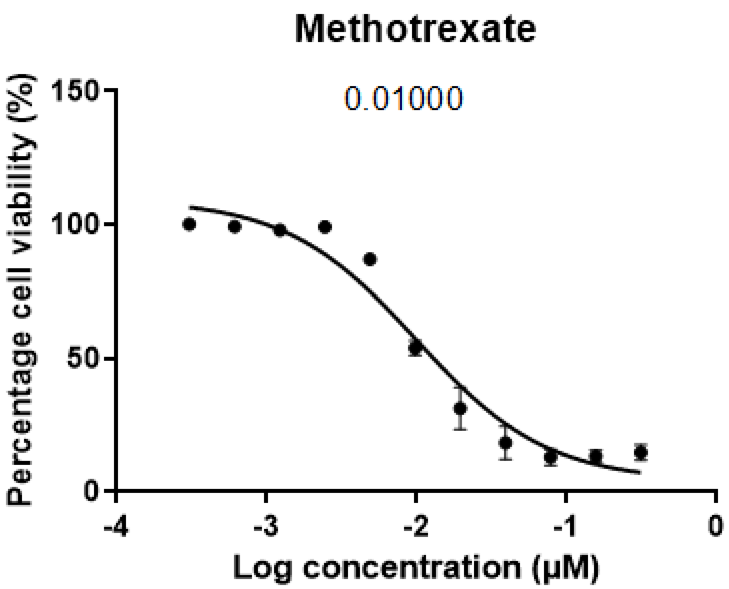
Methotrexate
0.01000
Percentage cell viability (%)
150
100
50
0
-4
-3
-2
-1
0
Log concentration (μM)

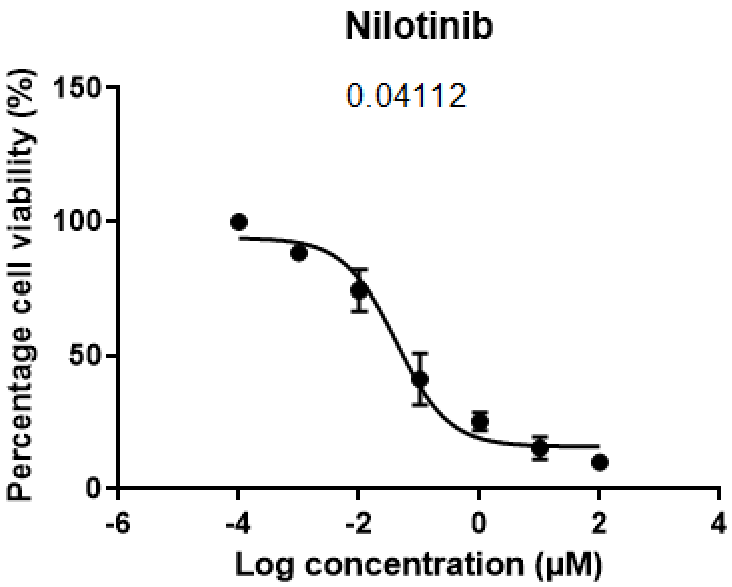
Nilotinib
0.04112
Percentage cell viability (%)
150
100
50
0
-6
-4
-2
0
2
4
Log concentration (μM)

Concentration-response curves for the most active compounds
(**16**, **18**, **36**, **56**, **73**, **74**, **77**, **78**, **81**, **100**, **101**, and **103**)
tested against SKBR-3 breast cancer cells

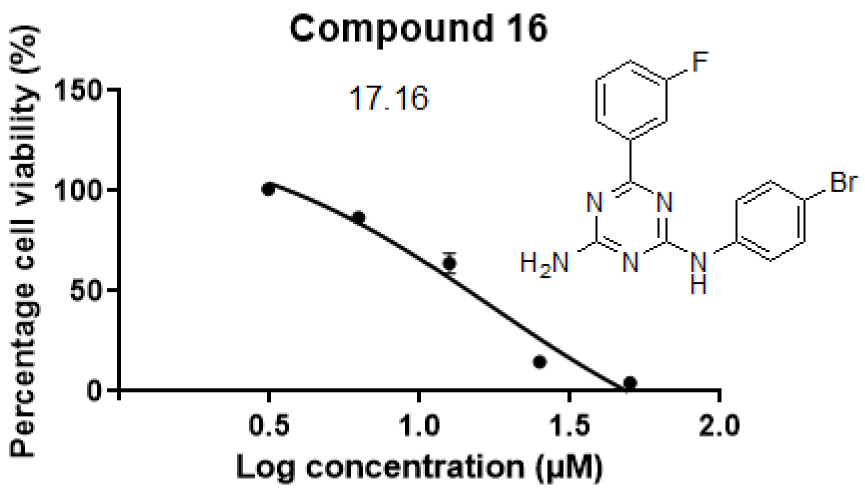
Compound 16
17.16
Percentage cell viability (%)
Log concentration (μM)
F
Br
N
N
N
H_2N
N
H

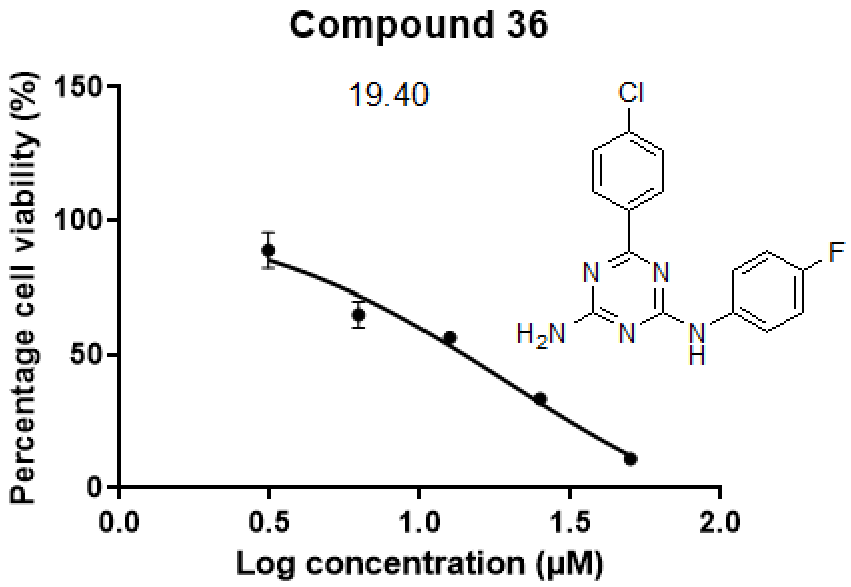
Compound 36
19.40
Percentage cell viability (%)
Log concentration (μM)
Cl
F
N
N
N
H_2N
N
H

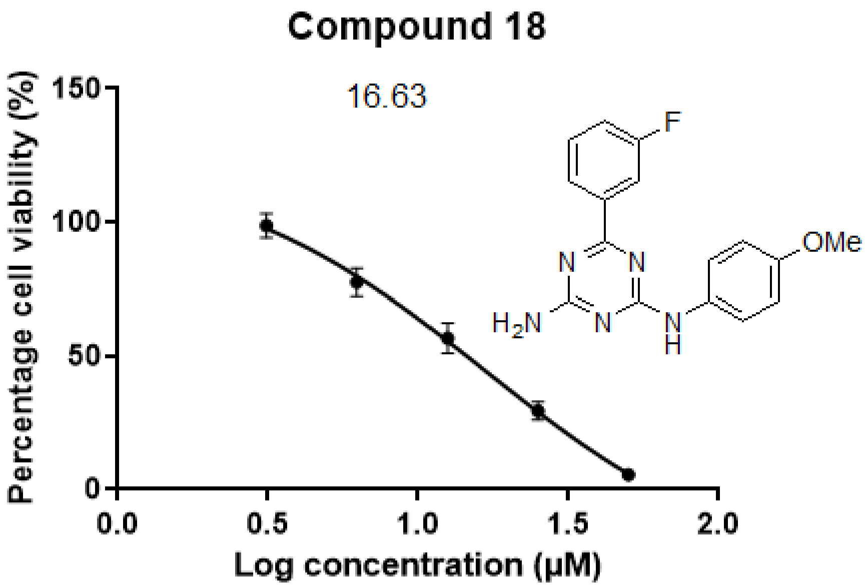
Compound 18
16.63
Percentage cell viability (%)
Log concentration (μM)
F
OMe
N
N
N
H_2N
N
H

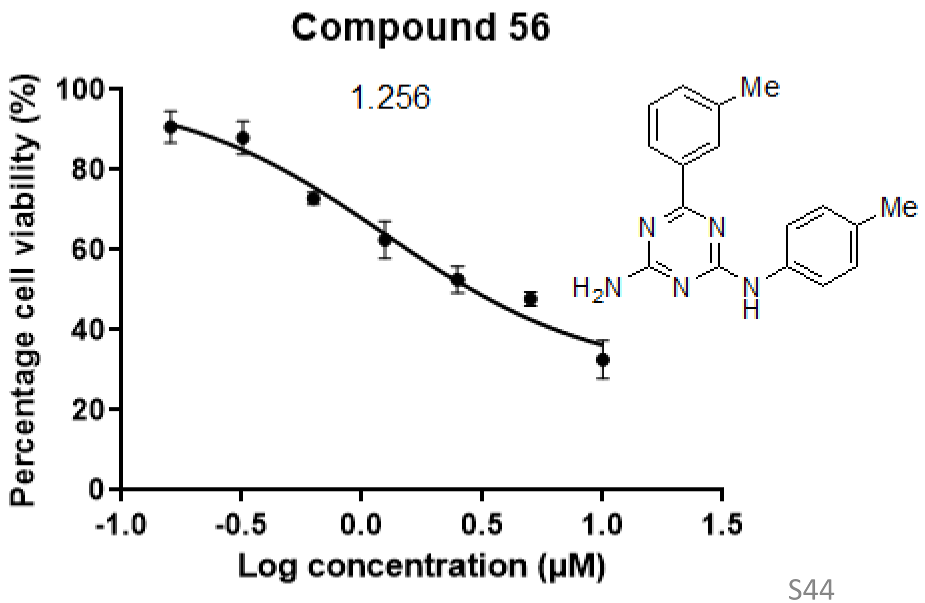
Compound 56
1.256
Percentage cell viability (%)
Log concentration (μM)
Me
Me
N
N
N
H_2N
N
H

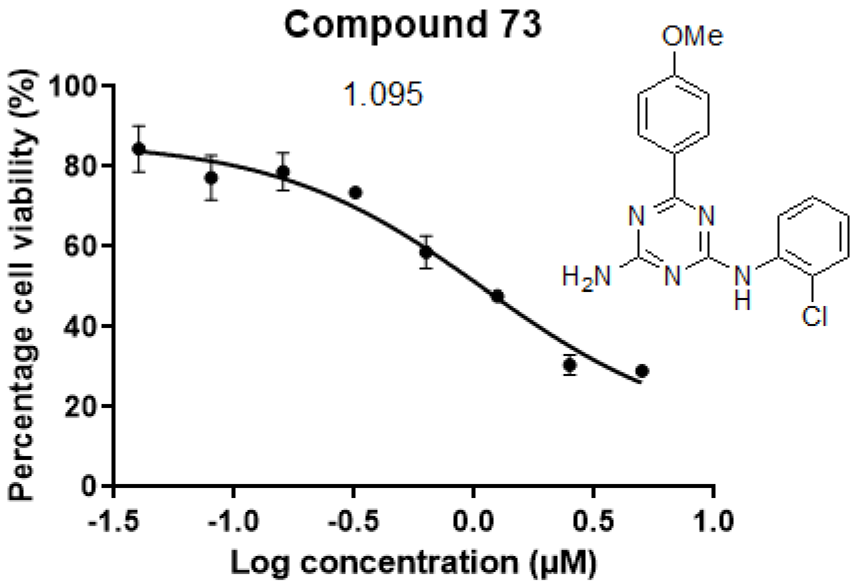
Compound 73
1.095
OMe
N
N
N
H2N
N
H
Cl
Percentage cell viability (%)
Log concentration (μM)
0
20
40
60
80
100
-1.5
-1.0
-0.5
0.0
0.5
1.0

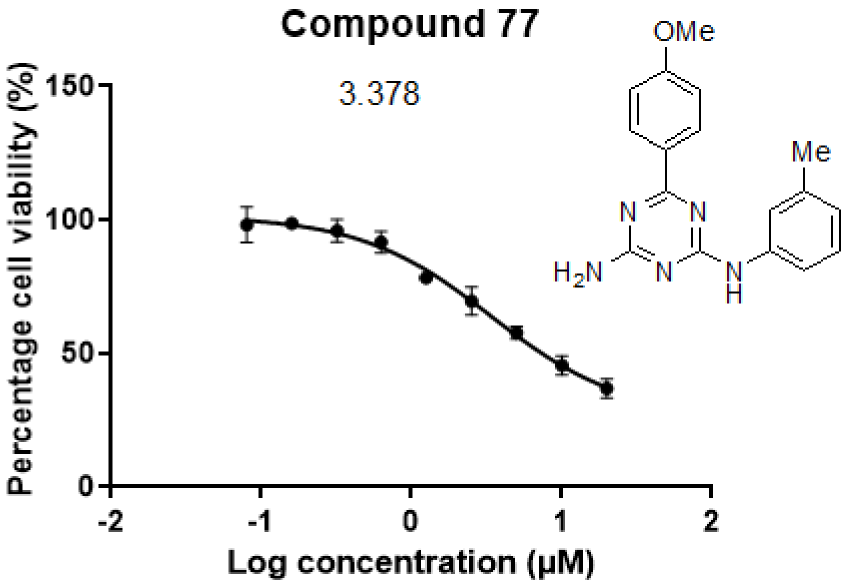
Compound 77
3.378
OMe
Me
N
N
N
H2N
N
H
Percentage cell viability (%)
Log concentration (μM)
0
50
100
150
-2
-1
0
1
2

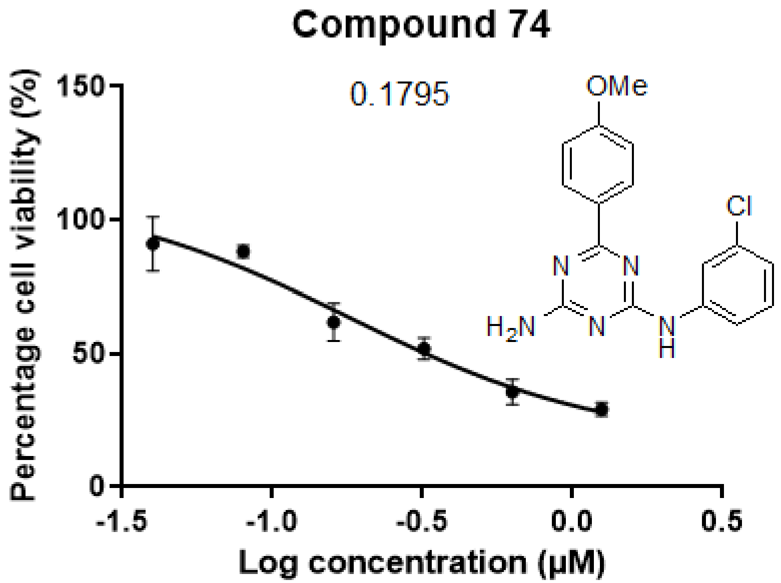
Compound 74
0.1795
OMe
Cl
N
N
N
H2N
N
H
Percentage cell viability (%)
Log concentration (μM)
0
50
100
150
-1.5
-1.0
-0.5
0.0
0.5

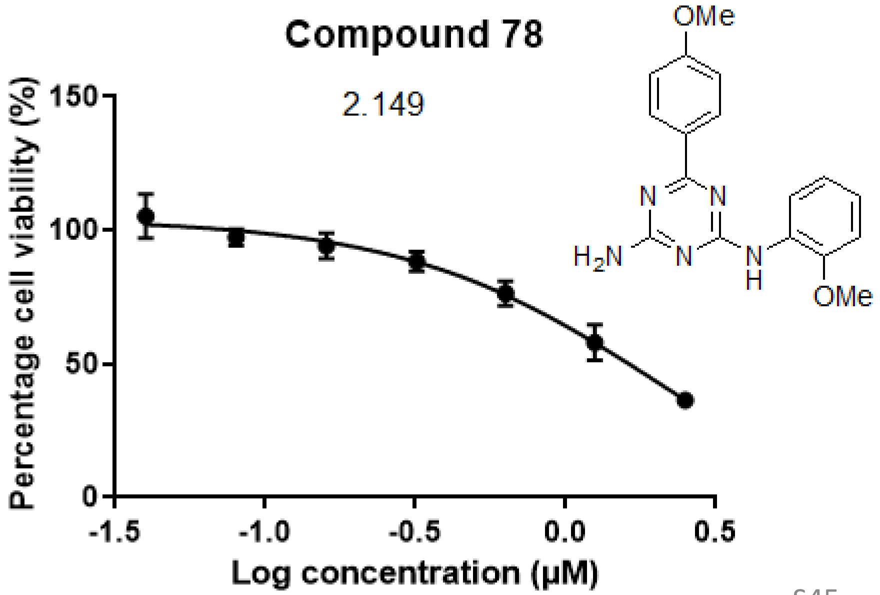
Compound 78
2.149
OMe
N
N
N
H2N
N
H
OMe
Percentage cell viability (%)
Log concentration (μM)
0
50
100
150
-1.5
-1.0
-0.5
0.0
0.5

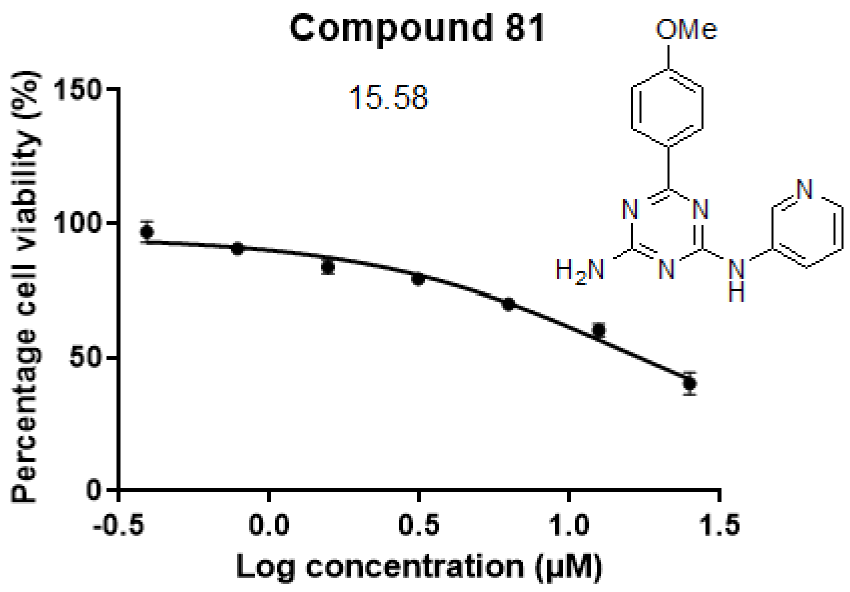
Compound 81
15.58
OMe
N
N
N
N
H_2N
N
H
Percentage cell viability (%)
150
100
50
0
-0.5
0.0
0.5
1.0
1.5
Log concentration (μM)

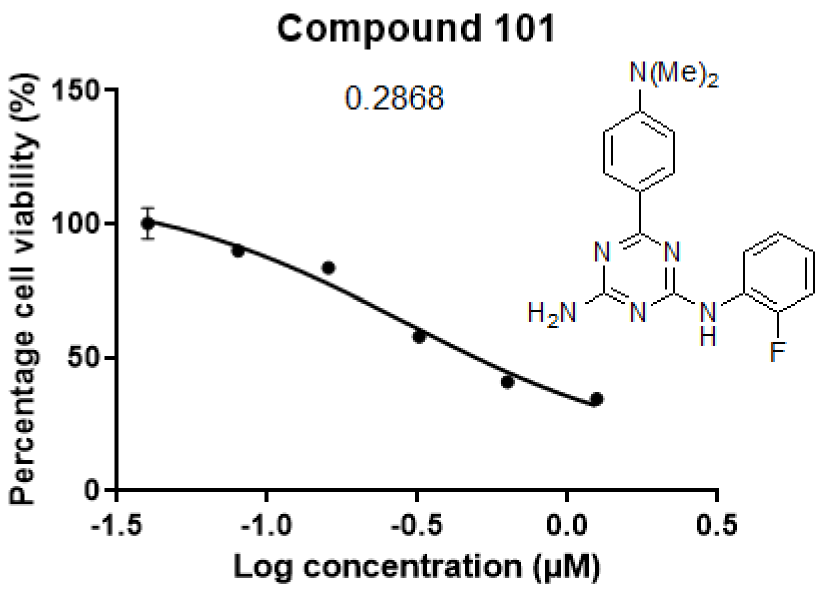
Compound 101
0.2868
N(Me)$_2$
N
N
N
H_2N
N
H
F
Percentage cell viability (%)
150
100
50
0
-1.5
-1.0
-0.5
0.0
0.5
Log concentration (μM)

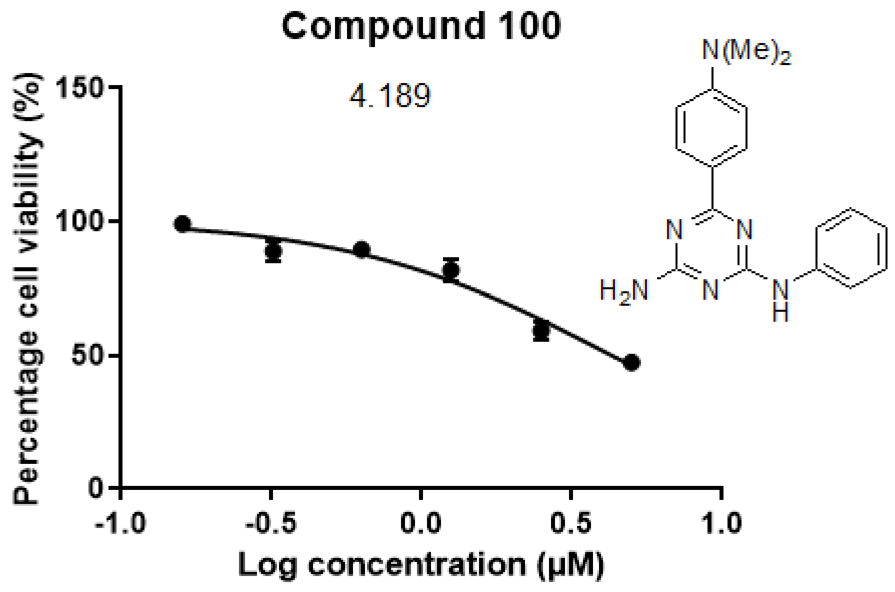
Compound 100
4.189
N(Me)$_2$
N
N
N
H_2N
N
H
Percentage cell viability (%)
150
100
50
0
-1.0
-0.5
0.0
0.5
1.0
Log concentration (μM)

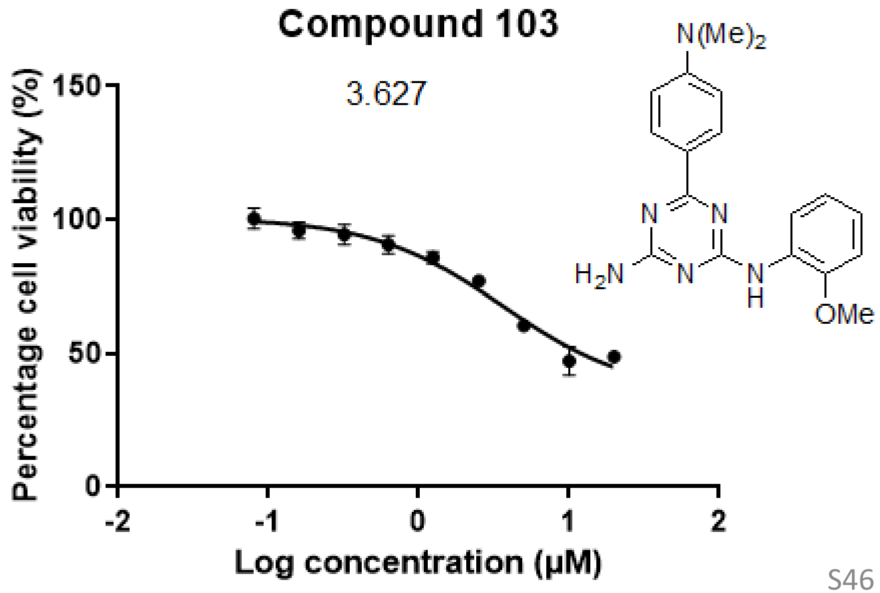
Compound 103
3.627
N(Me)$_2$
N
N
N
H_2N
N
H
OMe
Percentage cell viability (%)
150
100
50
0
-2
-1
0
1
2
Log concentration (μM)

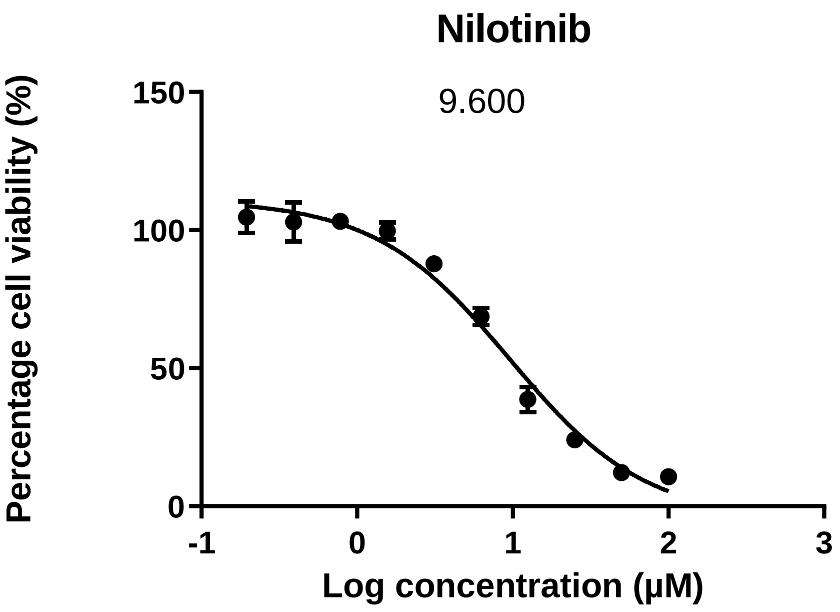
Nilotinib
9.600
Percentage cell viability (%)
150
100
50
0
-1
0
1
2
3
Log concentration (µM)
